# SAPCD2 Drives Bladder Cancer Progression by Stabilizing TANK and Engaging a CREB–PLAGL2 Feedback Loop to Sustain MAPK Signaling

**DOI:** 10.3390/cancers18030535

**Published:** 2026-02-06

**Authors:** Yueqiang Peng, Hai Wang, Hualin Chen, Zhaoheng Jin, Yingjie Li, Lin Ma, Zhigang Ji

**Affiliations:** 1 Department of Urology, Peking Union Medical College Hospital, Chinese Academy of Medical Sciences & Peking Union Medical College, No. 1 Shuaifuyuan, Dongcheng District, Beijing 100730, China; 2 Department of Geriatrics, Tongji Hospital, Tongji Medical College, Huazhong University of Science and Technology, 1095 Jiefang Avenue, Wuhan 430030, China

**Keywords:** SAPCD2, bladder cancer, MAPK signaling, TANK, positive feedback loop

## Abstract

**Background**: Bladder cancer (BCa) is a prevalent and aggressive malignancy characterized by high recurrence and metastasis rates. Despite advances in treatment, the prognosis for patients with advanced BCa remains poor. This study aimed to investigate the role of SAPCD2 in BCa progression and its potential as a therapeutic target. **Methods**: We performed a series of in vitro and in vivo experiments to assess the expression and function of SAPCD2 in BCa. The correlation between SAPCD2 expression and clinicopathological features was analyzed using tissue samples from BCa patients. Functional assays, including cell proliferation, migration, invasion, and metastasis tests, were conducted to evaluate the biological impact of SAPCD2. Mechanistic studies focused on the MAPK signaling pathway, TANK stabilization, and the interaction between SAPCD2 and the PLAGL2–CREB feedback loop. **Results**: Our results showed that SAPCD2 was significantly upregulated in BCa tissues and correlated with advanced clinicopathological features and poor prognosis. Overexpression of SAPCD2 promoted cell proliferation, migration, invasion, and metastasis, while its silencing led to the opposite effects. Mechanistically, SAPCD2 activated the MAPK signaling pathway by stabilizing TANK and preventing its degradation by SYVN1. Furthermore, we identified a positive feedback loop in which SAPCD2 enhanced PLAGL2 expression through CREB phosphorylation, further amplifying SAPCD2 expression and MAPK signaling. **Conclusions**: This study indicated that SAPCD2 could serve as a critical driver of BCa malignancy, emphasizing its role in sustaining oncogenic signaling through the SAPCD2–TANK–MAPK axis and the PLAGL2–SAPCD2–CREB feedback loop. Targeting this pathway may offer novel therapeutic strategies for treating aggressive BCa.

## Simple Summary

Bladder cancer is a common cancer that often relapses and can spread to other organs, making treatment difficult. To improve understanding of how bladder cancer becomes more aggressive, we studied a protein called SAPCD2. We found that SAPCD2 helps bladder cancer cells grow, survive, and spread by keeping cancer-related signaling active inside the cells. SAPCD2 does this by protecting another protein, TANK, from being broken down, which allows growth signals to continue. In addition, SAPCD2 is part of a self-reinforcing loop with other regulatory proteins that further strengthen these cancer-promoting signals. By uncovering how SAPCD2 drives these processes, our study provides new insight into bladder cancer biology and suggests several potential targets for future treatments aimed at slowing or stopping cancer progression.

## 1. Introduction

Bladder cancer (BCa) is among the most frequently diagnosed malignancies worldwide and continues to impose a substantial burden on public health [1]. Globally, more than 500,000 new cases are diagnosed each year, with a pronounced male predominance [2,3]. Despite continuous advances in diagnostic and therapeutic strategies, BCa remains responsible for over 200,000 cancer-related deaths annually [4]. Ongoing population ageing, together with sustained exposure to established risk factors—most notably tobacco smoking—is expected to further exacerbate the global incidence and mortality of this disease [5]. Clinically, BCa exhibits remarkable biological and clinical heterogeneity, resulting in unsatisfactory long-term outcomes for a considerable proportion of patients [6]. Although non-muscle-invasive BCa is generally associated with relatively favorable survival, its high recurrence rate necessitates intensive and lifelong surveillance [6]. In contrast, muscle-invasive and advanced-stage BCa are characterized by aggressive behavior, limited treatment options, and poor prognosis [7]. Current therapeutic approaches, including platinum-based chemotherapy, radical surgery, and immune checkpoint blockade, provide durable benefit to only a part of patients [8]. Moreover, conventional clinicopathological parameters show limited accuracy in predicting disease progression or treatment response [9]. Accordingly, there is an urgent need to identify novel molecular biomarkers that can improve early diagnosis, refine prognostic stratification, and facilitate the development of more effective personalized therapeutic strategies for BCa.

Suppressor anaphase-promoting complex domain containing 2 (SAPCD2), also referred to as p42.3 or C9orf140, is a cell cycle-associated protein encoded on chromosome 9q34.3 [10]. SAPCD2 was originally characterized as a mitosis-related factor with tightly regulated expression during cell cycle progression. Mechanistic studies revealed that SAPCD2 played an essential role in mitotic spindle orientation and symmetric cell division through negative regulation of the Gαi–LGN–NuMA complex, thereby influencing cell fate decisions and tissue organization during development [11]. Beyond its physiological functions, increasing evidence indicates that SAPCD2 was aberrantly upregulated in a wide spectrum of human malignancies and predominantly functioned as an oncogenic driver. Elevated expression of SAPCD2 has been documented in breast cancer [12], neuroblastoma [12], fibrosarcoma [13] and colorectal cancer [14], where it was frequently correlated with enhanced tumor proliferation, migration, invasion, metastatic capacity, and unfavorable clinical outcomes in vitro and in vivo models. Furthermore, accumulating evidence suggested that SAPCD2 participated in oncogenic signaling networks. For example, SAPCD2 has been reported to promote breast cancer cell migration and invasiveness through activation of YAP/TAZ signaling [12], while studies in other tumor contexts have linked SAPCD2 to Wnt/β-catenin-related regulatory circuits [15]. Moreover, MAPK-associated signaling has been implicated downstream of SAPCD2 in melanoma [16]. Notwithstanding these advances, the biological role and clinical relevance of SAPCD2 in BCa have not yet been systematically investigated.

Given its reported involvement in tumor progression in other malignancies, we focused on SAPCD2 as a candidate oncogenic regulator in BCa. Using a combination of gain- and loss-of-function approaches in BCa cell lines and in vivo tumor models, we examined the effects of SAPCD2 on malignant phenotypes. In parallel, we explored the signaling pathways and regulatory networks associated with SAPCD2, with particular emphasis on its potential involvement in MAPK signaling and upstream transcriptional regulation. Therefore, we sought to investigate whether SAPCD2 contributed to the progression of BCa and to elucidate the molecular mechanisms underlying its oncogenic function in this study.

## 2. Materials and Methods

### 2.1. Public Database Data Mining

We retrieved clinical and transcriptomic data for BCa from The Cancer Genome Atlas (TCGA) portal (https://portal.gdc.cancer.gov/, accessed on 20 September 2024). To serve as a control, transcriptomic data from normal bladder tissues were sourced from GTEx via UCSC Xena (https://xena.ucsc.edu/, accessed on 21 September 2024). For normal tissue gene expression profiles, we consulted the Human Protein Atlas (HPA) database (https://www.proteinatlas.org/, accessed on 3 May 2025). Protein–protein interactions prediction was performed by using GeneMANIA (https://genemania.org/, accessed on 11 May 2025) and the BioGRID database (https://thebiogrid.org/, accessed on 11 May 2025). Ubiquitination sites were predicted with the UbiBrowser tool (http://ubibrowser.ncpsb.org.cn, accessed on 7 June 2025). To identify upstream transcriptional regulators, predictions from JASPAR (https://jaspar.elixir.no/, accessed on 1 July 2025), hTFtarget (https://guolab.wchscu.cn/hTFtarget/, accessed on 1 July 2025), and GeneCards (https://www.genecards.org/, accessed on 1 July 2025) were carried out. Single-cell RNA-seq data were downloaded from GEO (GSE130001; https://www.ncbi.nlm.nih.gov/geo/, accessed on 16 January 2025). All analyses were performed using R (version 4.4.1).

### 2.2. Cell Culture

Human BCa cell lines T24, UMUC3, J82, and 5637, together with the normal human urothelial cell line SV-HUC-1, were purchased from Procell (Wuhan, China). Among these, T24, UMUC3, and J82 cells are commonly used as models of highly invasive urothelial carcinoma, whereas 5637 cells are derived from a grade II urothelial carcinoma. T24 cells were cultured in McCoy’s 5A medium, while 5637 cells were maintained in RPMI-1640 medium (Procell, China). UMUC3 and J82 cells were grown MEM (Procell, China), whereas SV-HUC-1 cells were cultured in F-12K medium (Procell, China). All media were supplemented with 10% fetal bovine serum (Servicebio, Wuhan, China) and 1% penicillin–streptomycin solution (Servicebio, China). Cells were maintained at 37 °C in a humidified atmosphere containing 5% CO_2_.

### 2.3. Quantitative Real-Time PCR (qRT-PCR)

Total RNA was isolated with TRIzol (Invitrogen, Carlsbad, CA, USA). cDNA was generated using a reverse transcription kit (Servicebio, China). qRT–PCR was carried out with SYBR Green Mix (Servicebio), and relative expression was calculated by the 2^−ΔΔCt^ method with GAPDH as the internal control. Primer sequences are listed in Supplementary Table S1.

### 2.4. Western Blot (WB)

Cells were lysed in RIPA buffer containing protease/phosphatase inhibitors (Servicebio, China). Protein concentration was measured by BCA (Servicebio). Equal amounts of protein were separated by SDS–PAGE, transferred to PVDF membranes, blocked with 5% milk, and incubated with primary antibodies overnight at 4 °C, followed by HRP-conjugated secondary antibodies. Bands were visualized using ECL (NCM Biotech, Suzhou, China) and quantified in ImageJ (version 1.5.1), normalized to the indicated loading controls. Antibody details are listed in Supplementary Table S2.

### 2.5. siRNA and Plasmid Transfection

siNC and siRNAs targeting SAPCD2 (two sequences, si-1 and si-2), PLAGL2, and CREB were purchased from GenePharma (Shanghai, China) (sequences in Table S3). Cells were transfected with siRNAs using Lipofectamine 3000 (Invitrogen, USA). Knockdown was confirmed by qRT–PCR and/or WB. For overexpression, full-length SAPCD2 and TANK were cloned into pcDNA3.1(+) (Thermo Fisher Scientific, Waltham, MA, USA). Plasmids were introduced with Lipofectamine 3000, and expression was validated by qRT–PCR and WB.

### 2.6. Lentiviral Plasmid Construction and Transduction

Lentiviruses carrying PLAGL2 or TANK overexpression constructs, as well as lentiviruses encoding specific shRNAs targeting PLAGL2 or TANK, were designed and packaged by GeneChem (Shanghai, China). Corresponding negative control lentiviruses containing empty vectors or non-targeting shRNAs were used as controls. The shRNA target sequences are provided in Table S4. Cells were seeded in 24-well plates and transduced according to the manufacturer’s instructions. After transduction, stable cell populations were selected and expanded for subsequent experiments.

### 2.7. Immunohistochemistry (IHC)

Paraffin-embedded tissue sections were deparaffinized in xylene, rehydrated through graded ethanol, and subjected to heat-induced antigen retrieval. After blocking endogenous peroxidase activity and nonspecific binding, sections were incubated overnight at 4 °C with the indicated primary antibodies, including anti-SAPCD2 antibody (Bioss, Beijing, China; BS-15314R; 1:200 dilution) and anti-Ki67 antibody (Proteintech, Wuhan, China; 27309-1-AP; 1:10,000 dilution). Following incubation with appropriate HRP-conjugated secondary antibodies, immunoreactive signals were visualized using diaminobenzidine (DAB) and counterstained with hematoxylin.

Immunostaining was independently evaluated by two experienced pathologists who were blinded to the clinical information. Staining scores were determined by combining the staining intensity and the percentage of positively stained tumor cells. Staining intensity was graded as 0 (negative), 1 (weak), 2 (moderate), or 3 (strong), and the proportion of positive cells was scored as 0 (<5%), 1 (5–25%), 2 (26–50%), 3 (51–75%), or 4 (>75%). The final immunoreactivity score was calculated by multiplying the intensity score by the percentage score. In cases of discrepant evaluations, a consensus score was reached after joint review.

BCa tissue samples were obtained from patients who underwent surgical resection at our institution with written informed consent. Detailed clinicopathological characteristics are summarized in Table 1 and Supplementary Table S5.

### 2.8. Colony Formation Assay

Cells were seeded into six-well plates at a density of 500 cells per well and cultured under standard conditions for approximately 10 days to allow colony formation. After incubation, colonies were fixed with paraformaldehyde and stained with crystal violet. Visible colonies were photographed and counted.

### 2.9. Cell Counting Kit-8 Assay

Cells were seeded into 96-well plates at a density of 2 × 10^3^ cells per well and cultured under standard conditions. At the indicated time points, CCK-8 reagent (Beyotime, Shanghai, China) was added to each well according to the manufacturer’s instructions, followed by incubation at 37 °C. Absorbance was measured at 450 nm using a microplate reader.

### 2.10. Wound Healing Assay

Cells were seeded into six-well plates and grown to approximately 90–100% confluence. A linear scratch was created using a sterile 200 µL pipette tip, and detached cells were removed by gentle washing with phosphate-buffered saline (PBS). Cells were then cultured in serum-free medium. Wounds were photographed at 0 h and 24 h, and closure was quantified in ImageJ.

### 2.11. Transwell Assay

Cell invasion was assessed using Transwell chambers with 8 µm pore size inserts (Corning, Corning, NY, USA), precoated with (Invasion) or without (Migration) Matrigel (Beyotime, China). Cells were serum-starved, resuspended in serum-free medium, and seeded into the upper chamber at a density of 1.5 × 10^5^ cells per insert. Medium containing 10% fetal bovine serum was added to the lower chamber as a chemoattractant. After incubation for 24 h at 37 °C, non-invading cells on the upper surface were removed with a cotton swab, whereas invaded cells on the lower surface were fixed, stained with crystal violet, photographed, and counted under a microscope. For quantification, cells were counted in four randomly selected microscopic fields per insert, and the average number of cells was calculated.

### 2.12. Cell Cycle and Apoptosis

Apoptosis was assessed using an Annexin V/propidium iodide (PI) double-staining assay. Cells were harvested with EDTA-free trypsin, washed with cold PBS and stained with Annexin V and propidium iodide (PI) according to the manufacturer’s instructions (Beyotime, China). Apoptotic cells were analyzed by flow cytometry. For cell cycle analysis, cells were collected after treatment, washed with PBS, and fixed in 70% ethanol at 4 °C overnight. After fixation, cells were incubated with PI and RNase-containing staining solution and subjected to flow cytometric analysis to determine cell cycle distribution.

### 2.13. RNA Sequencing and Analysis

Total RNA was extracted from T24 cells following siRNA-mediated knockdown and subjected to high-throughput RNA sequencing. Differentially expressed genes (DEGs) were identified based on the criteria of an |log2(FoldChange)| ≥ 1.0 and a *p*-value < 0.05. All downstream bioinformatic analyses were conducted using R software (Version 4.4.1). Gene Ontology (GO), Kyoto Encyclopedia of Genes and Genomes (KEGG) and Gene Set Enrichment Analysis (GSEA) enrichment analysis was performed using the clusterProfiler R package (version 4.8.1).

### 2.14. Co-Immunoprecipitation (Co-IP)

Co-IP was performed with the Pierce Classic Magnetic IP/Co-IP Kit (Thermo Fisher Scientific, Waltham, MA, USA). Cells were lysed on ice in IP lysis/wash buffer supplemented with protease inhibitors, and clarified lysates were obtained by centrifugation. For each reaction, 500–1000 µg total protein was combined with 2–10 µg specific antibody (or IgG), topped up to 500 µL with IP lysis/wash buffer, and left to allow immune-complex formation (1 h at room temperature or overnight at 4 °C). Meanwhile, 25 µL protein A/G magnetic beads were washed as recommended, then added to the mixture and rotated for 1 h at room temperature. After binding, the beads were rinsed three times with 500 µL IP lysis/wash buffer and once briefly with water to reduce background. Complexes were released with 100 µL elution buffer for 10 min and immediately neutralized (10 µL neutralization buffer per 100 µL eluate); when needed, elution was instead performed directly in 1× sample buffer for SDS–PAGE. The eluates were finally subjected to immunoblotting with the indicated antibodies

### 2.15. Molecular Docking

Rigid protein–protein docking was performed using the HDOCK platform. Three-dimensional structures of the target proteins were retrieved from the UniProtKB database based on their corresponding gene names. The protein structures were submitted to the HDOCK server, and docking calculations were carried out using default protein–protein docking parameters. The top 10 predicted docking models were collected according to the HDOCK scoring system, with the top-ranked model considered the most favorable binding conformation. Binding free energy was used as a primary evaluation criterion, with values lower than −30 kcal/mol indicating stable interactions. Following docking, binding free energy was further estimated using HawkDock, and molecular interaction features, including interaction interface, hydrogen bonding, and key amino acid residues, were analyzed. Docking results were visualized using PyMOL (version 3.1). It should be noted that molecular docking was applied as a supportive in silico approach to provide structural insight into the potential protein interaction and does not represent experimental structural validation.

### 2.16. Dual-Luciferase Reporter Assay

Promoter regions of SAPCD2 and PLAGL2 were amplified and inserted upstream of the luciferase coding sequence in the pGL3-Basic vector (Promega, Madison, WI, USA) to generate the corresponding wild-type reporter constructs (WT-SAPCD2 and WT-PLAGL2). To assess sequence-specific regulatory effects, matched mutant promoters carrying the intended binding-site substitutions were produced and cloned into the same vector to obtain MUT-SAPCD2 and MUT-PLAGL2 reporters. Cells were plated in advance and co-transfected with the indicated pGL3 reporter plasmids together with the relevant expression plasmid (or control vector) using standard transfection procedures. After 48 h, luciferase signals were quantified with the Dual-Luciferase^®^ Reporter Assay System (Promega) according to the manufacturer’s guidelines.

### 2.17. Chromatin Immunoprecipitation (ChIP)

ChIP was conducted with the BeyoChIP™ ChIP Assay Kit (Beyotime, China). Cells were fixed with formaldehyde, and the reaction was stopped with glycine. After lysis, chromatin was incubated with an anti-PLAGL2 antibody; IgG (Proteintech, China) served as the negative control. Protein–DNA complexes were collected on protein A/G magnetic beads, washed stringently, and then eluted. Crosslinks were reversed, proteins were digested, and DNA was purified for qPCR. qPCR targeted three predicted PLAGL2-binding regions in the SAPCD2 promoter using the following primers: site 1 (F: 5′-GCTGACCGCACTACATCCTT-3′; R: 5′-CCTAGAAGGAGCCCTGTCCT-3′), site 2 (F: 5′-ACCCCCTCCAAAGATCCCAC-3′; R: 5′-AGCCTACGATCCTCTTGGGG-3′), and site 3 (F: 5′-ACGGCGACAATAGCGACTAC-3′; R: 5′-GCGGCGCATGTTAATGAGG-3′). Enrichment was normalized to input DNA, with the IgG signal treated as background.

### 2.18. In Vivo Experiment

Stable BCa cell lines were used to establish xenograft and metastasis models in 4-week-old female BALB/c nude mice. The mice were obtained from SPF Biotechnology (Beijing, China) and housed in a barrier facility under specific pathogen-free conditions with a 12 h light/dark cycle. The animals had not undergone any previous experimental procedures prior to enrollment in this study. The experimental unit was defined as a single mouse.

For subcutaneous growth, 5 × 10^6^ cells were injected into the flank. Tumor size and body weight were recorded every 3 days, and volume was calculated as (length × width^2^)/2.

For experimental metastasis, 1 × 10^6^ luciferase-labeled cells were delivered via tail vein injection. Metastatic burden was evaluated by bioluminescence imaging, followed by sacrifice and collection of lungs for fixation in 4% paraformaldehyde and H&E staining to assess metastatic nodules.

The animal experiments were conducted with 6 mice in each group, and a total of 72 mice were used. Sample sizes were determined based on prior experience with similar xenograft models and on commonly used group sizes reported in the literature, which are sufficient to detect biologically meaningful differences in tumor growth and metastatic burden. All animals injected with viable tumor cells and surviving until the predefined experimental endpoint were included in the analyses. No animals, experimental units, or data points were excluded. Randomization was performed using a simple random number generator. Animals were age- and sex-matched and housed under identical conditions. Tumor cell injections and measurements were performed using standardized procedures at comparable time points across groups. No additional measures were taken to control for the order of measurements or cage location. Group allocation was performed by one investigator, who was not involved in outcome assessment or data analysis. Tumor measurements and data analysis were conducted by independent investigators who were unaware of group allocation.

This experiment was approved by the Animal Welfare Ethics Committee of Beijing MDKN Biotechnology Co., Ltd. (Approval Number: MDKN-2025-063), and was conducted in strict accordance with the experimental animal care and use guidelines of the Beijing Animal Control Committee.

### 2.19. Statistical Analysis

All quantitative data are presented as the mean ± SD. Data distribution was assessed prior to statistical analysis. Differences between the two groups were assessed using Student’s *t*-test when data met normality assumptions, while comparisons among multiple groups were evaluated by one-way ANOVA. When assumptions for parametric tests were not met, appropriate non-parametric methods were applied. Differences between categorical variables were analyzed using the chi-square test or Fisher’s exact test, as appropriate. Survival analyses were performed using the Kaplan–Meier method. Correlations between variables were assessed using Pearson or Spearman correlation analysis, depending on data distribution. Statistical significance was defined as a two-sided *p* value < 0.05. Data visualization and statistical analyses were conducted using GraphPad Prism software (version 9.0; La Jolla, CA, USA).

## 3. Results

### 3.1. SAPCD2 Is Upregulated in BCa and Correlates with Aggressive Clinicopathological Features

**Figure 1:**
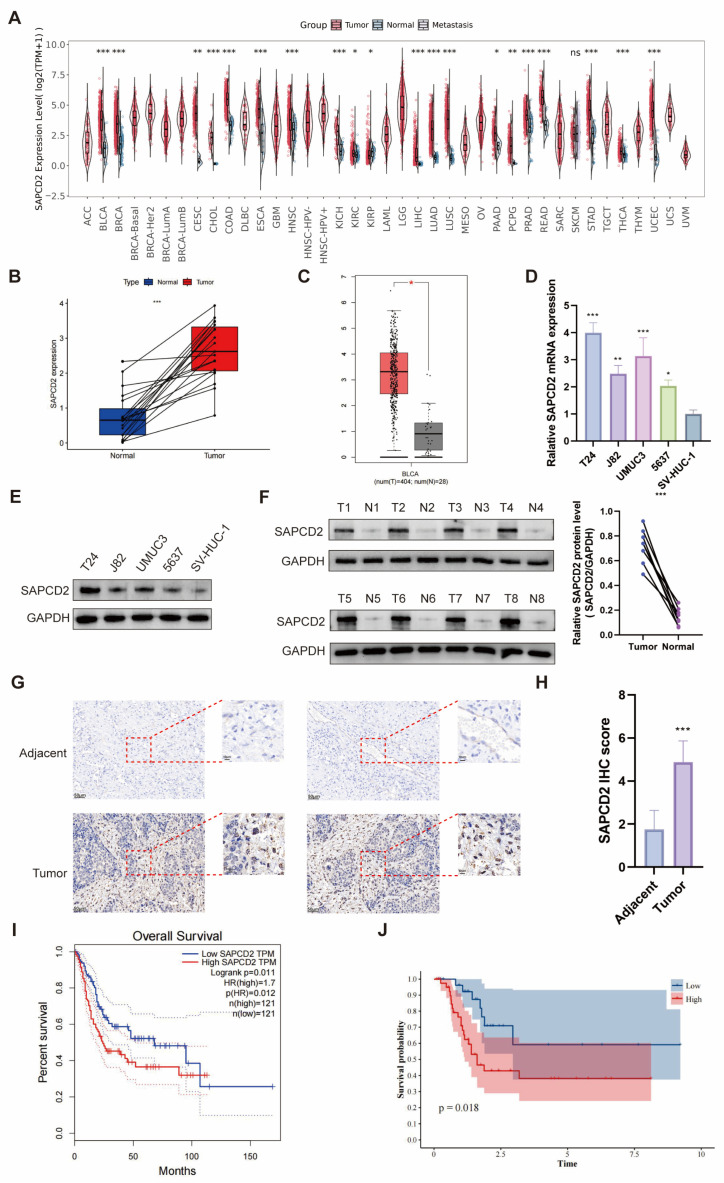
SAPCD2 Expression in Bladder Cancer. (**A**) Pan-cancer analysis of SAPCD2 expression in 21 tumor types, comparing tumor tissues to normal controls. (**B**) Comparison of SAPCD2 expression between tumor and adjacent normal tissues in TCGA paired bladder cancer samples. (**C**) SAPCD2 expression in bladder cancer (Red) compared to normal bladder tissue (Grey) using TCGA combined with GTEx data. (**D**) SAPCD2 mRNA expression in bladder cancer cell lines (T24, UMUC3, J82, 5637) and normal urothelial cell line (SV-HUC-1). (**E**) SAPCD2 protein expression in bladder cancer cell lines (T24, UMUC3, J82, 5637) and normal urothelial cell line (SV-HUC-1). (**F**) Protein level of SAPCD2 in tumor and adjacent tissues from 8 patients. (**G**) IHC staining of SAPCD2 in tumor and adjacent tissues. (**H**) Quantification of SAPCD2 IHC scores from tumor and adjacent tissues (Original magnification, ×20; scale bar = 50 μm). (**I**) Kaplan–Meier survival analysis showing SAPCD2 expression and overall survival in bladder cancer patients. (**J**) Survival analysis based on SAPCD2 expression in a cohort of 71 bladder cancer patients in our institution. Not significant (ns), *p* < 0.05 (*), *p* < 0.01 (**) and *p* < 0.001 (***). The uncropped blots are shown in File S1.

Pan-cancer analysis showed that SAPCD2 expression was significantly higher in tumor tissues than in corresponding normal controls in 19 of 21 cancer types with available paired normal samples, including BCa (Figure 1A). Consistently, paired analysis of TCGA BCa samples indicated higher SAPCD2 expression in tumor tissues compared with adjacent non-tumor tissues in all 19 matched cases (Figure 1B). When normal bladder tissue expression data from the GTEx database were included, SAPCD2 expression remained elevated in BCa samples (Figure 1C). Analysis of SAPCD2 expression across normal human tissues showed that SAPCD2 was predominantly expressed in the cerebral cortex and small intestine, whereas relatively low expression was observed in normal bladder tissue (Figure S1A).

SAPCD2 mRNA and protein levels were higher in four BCa cell lines compared with the normal human urothelial cell line (Figure 1D,E). In addition, analysis of protein extracted from tumor tissues and paired adjacent tissues from eight patients who underwent radical cystectomy revealed increased SAPCD2 expression in tumor samples (Figure 1F). Immunohistochemical staining of corresponding pathological sections showed stronger SAPCD2 staining in cancer tissues than in adjacent non-tumor tissues (Figure 1G,H).

To further examine SAPCD2 expression at the single-cell level, the GSE130001 single-cell RNA sequencing dataset was analyzed. SAPCD2 expression was enriched in cell clusters annotated as tumor cells, whereas lower expression was observed in non-malignant cell populations (Figure S1B–D). Consistent with these findings, higher SAPCD2 expression was associated with advanced metastatic stage (Figure S1E) and higher tumor grade (Figure S1F) in BCa patients from the TCGA cohort.

Pan-cancer survival analyses suggested that SAPCD2 expression was associated with overall survival (OS) and disease-free survival (DFS) across multiple tumor types, including BCa (Figure 1I and Figure S2A–R). To further evaluate its prognostic relevance in BCa, a retrospective cohort of 71 patients from our institution was analyzed. Patients were stratified into high- and low-SAPCD2 expression groups based on immunohistochemical staining intensity, and higher SAPCD2 expression was associated with poorer OS (Figure 1J). The clinicopathological characteristics of these patients are summarized in Table 1. Higher SAPCD2 expression was also associated with higher tumor grade and advanced T stage in this cohort.

### 3.2. SAPCD2 Knockdown Suppresses Malignant Behaviors of BCa Cells

**Figure 2:**
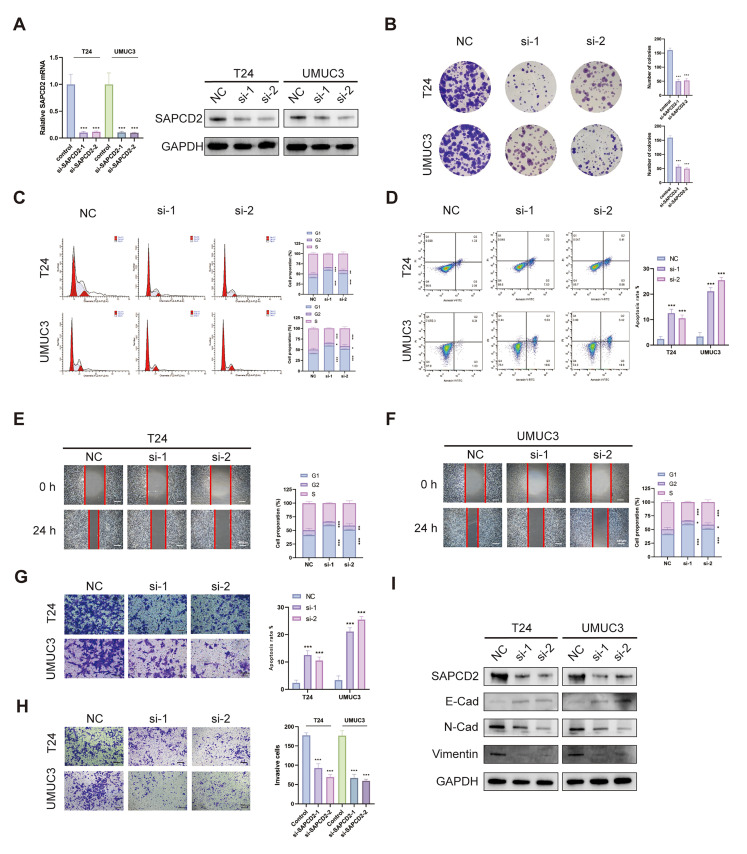
SAPCD2 knockdown suppresses Malignant Characteristics of Bladder Cancer Cells. (**A**) Confirmation of SAPCD2 knockdown (si-1, si-2) in T24 and UMUC3 cell lines, verified by qPCR and Western blot analysis. (**B**) Colony formation assay showing the number of colonies formed by SAPCD2-knockdown cells in comparison to control (NC). (**C**) Flow cytometry assays illustrating changes in the cell cycle phases upon SAPCD2 knockdown. (**D**) Apoptosis analysis performed by flow cytometry, comparing apoptotic rates between SAPCD2 knockdown and control cells. (**E**,**F**) Wound healing assay to assess migration capacity of SAPCD2-knockdown UMUC3 and T24 cells at 0 and 24 h. The red lines represent the edge of the cell scratch (Original magnification, ×4; scale bar = 400 μm). (**G**) Transwell migration assay quantifying migration ability of SAPCD2-knockdown cells compared to control (Original magnification, ×10; scale bar = 200 μm). (**H**) Transwell invasion assay showing the invasive potential of SAPCD2-knockdown cells (Original magnification, ×10; scale bar = 200 μm). (**I**) Protein level of EMT-related markers (E-cadherin, N-cadherin, vimentin) in T24 and UMUC3 cells after SAPCD2 knockdown. *p* < 0.05 (*), *p* < 0.01 (**) and *p* < 0.001 (***). The uncropped blots are shown in File S1.

We first noted that SAPCD2 expression was relatively higher in T24 and UMUC3 cells compared with other BCa cell lines. These two cell lines were, therefore, selected for subsequent loss-of-function studies. SAPCD2 expression was efficiently silenced in T24 and UMUC3 cells using siRNA-mediated knockdown, as confirmed by quantitative real-time PCR and WB (Figure 2A).

Depletion of SAPCD2 was associated with reduced proliferative capacity in BCa cells. Colony formation assays showed a decrease in the number of colonies formed by SAPCD2-silenced T24 and UMUC3 cells (Figure 2B). Consistent with this observation, EdU incorporation assays showed a reduced proportion of EdU-positive cells following SAPCD2 knockdown (Figure S3A,B). In addition, CCK-8 assays indicated a decrease in cell proliferation rates in SAPCD2-depleted T24 and UMUC3 cells (Figure S3C,D).

Cell cycle distribution analysis showed an increased proportion of cells in the G1 phase and a corresponding reduction in S-phase populations following SAPCD2 knockdown (Figure 2C). In parallel, flow cytometric analysis using Annexin V/PI staining showed an increase in apoptotic cell populations in both T24 and UMUC3 cells after SAPCD2 silencing (Figure 2D).

The effects of SAPCD2 depletion on cell motility were further examined. Wound healing assays showed delayed wound closure in SAPCD2-silenced cells compared with control cells (Figure 2E,F). Similarly, Transwell migration assays showed a reduction in migratory capacity following SAPCD2 knockdown (Figure 2G). Transwell invasion assays also showed decreased invasive capacity in SAPCD2-depleted BCa cells (Figure 2H). Analysis of epithelial–mesenchymal transition (EMT)-related markers showed decreased expression of mesenchymal markers and increased expression of epithelial markers following SAPCD2 knockdown (Figure 2I).

### 3.3. SAPCD2 Overexpression Enhances Malignant Characteristics of BCa Cells

**Figure 3:**
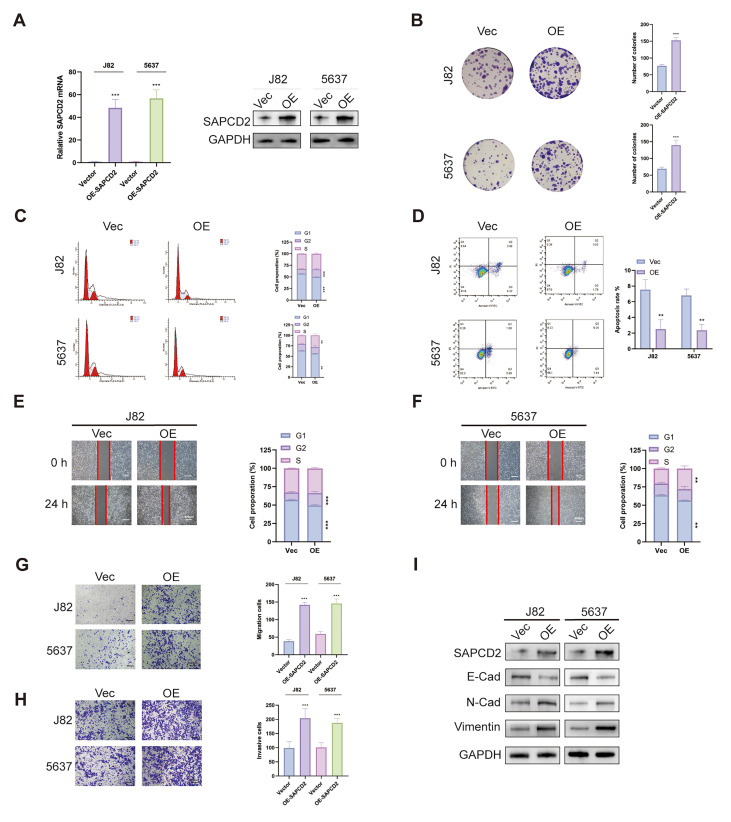
SAPCD2 Overexpression Enhances Malignant Characteristics of Bladder Cancer Cells. (**A**) Confirmation of SAPCD2 overexpression (OE) in J82 and 5637 bladder cancer cell lines following stable transfection, verified by qPCR and Western blot. (**B**) The number of colonies formed by SAPCD2-knockdown cells in comparison to control (Vec). (**C**) Flow cytometry assays indicating changes in the cell cycle phases upon SAPCD2 overexpression. (**D**) Flow cytometry assays showing a reduction in the percentage of apoptotic cells upon SAPCD2 overexpression. (**E**,**F**) Wound healing assay showed the migration capacity of SAPCD2-overexpressing J82 and 5637 cells at 0 and 24 h. The red lines represent the edge of the cell scratch (Original magnification, ×4; scale bar = 400 μm). (**G**) Migration assay using Transwell chambers (Original magnification, ×10; scale bar = 200 μm). (**H**) Invasion assay showing invasive potential of cells overexpressing SAPCD2 (Original magnification, ×10; scale bar = 200 μm). (**I**) EMT marker expression illustrated by Western blot. *p* < 0.01 (**) and *p* < 0.001 (***). The uncropped blots are shown in File S1.

SAPCD2 expression was relatively lower in J82 and 5637 BCa cell lines compared with other BCa cell lines, and these cells were selected for gain-of-function analyses. Stable overexpression of SAPCD2 in J82 and 5637 cells was confirmed at both the mRNA and protein levels by quantitative real-time PCR and Western blotting (Figure 3A).

SAPCD2 overexpression was associated with increased proliferative capacity in BCa cells. Clonogenic assays revealed an increased number of colonies formed by SAPCD2-overexpressing J82 and 5637 cells compared with control cells (Figure 3B). Consistent with this observation, EdU incorporation assays indicated a higher proportion of EdU-positive cells in SAPCD2-overexpressing cells (Figure S3E,F), and CCK-8 assays further supported increased cell proliferation rates (Figure S3G,H).

Cell cycle distribution analysis indicated an increased proportion of cells in the S and G2/M phases and a corresponding reduction in the G1-phase population following SAPCD2 overexpression (Figure 3C). In parallel, apoptosis assays revealed a reduced proportion of apoptotic cells in SAPCD2-overexpressing cells compared with controls (Figure 3D).

The effects of SAPCD2 overexpression on cell motility were further examined. Wound healing assays demonstrated accelerated wound closure in SAPCD2-overexpressing cells compared with control cells (Figure 3E,F). These findings were consistent with Transwell migration assays, in which increased migratory capacity was observed following SAPCD2 overexpression (Figure 3G). Transwell invasion assays further indicated increased invasive capacity in SAPCD2-overexpressing BCa cells (Figure 3H). Analysis of EMT-related markers revealed increased expression of mesenchymal markers and decreased expression of epithelial markers in SAPCD2-overexpressing cells (Figure 3I).

### 3.4. SAPCD2 Modulates BCa Growth and Metastatic Potential In Vivo

**Figure 4:**
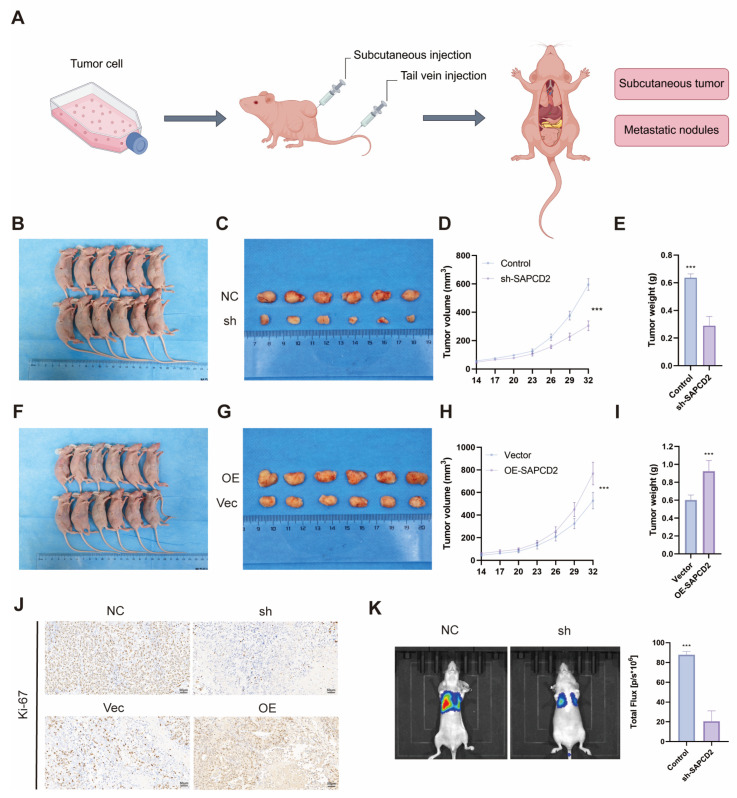
SAPCD2 Overexpression and Knockdown in Bladder Cancer Xenograft Models. (**A**) Schematic overview of the subcutaneous xenograft model with bladder cancer cells, illustrating the injection of stable SAPCD2 knockdown (sh-SAPCD2, T24) or overexpression (OE-SAPCD2, 5637) cells into mice. (**B**) Images of mice bearing tumors derived from T24 cells with stable SAPCD2 knockdown or control cells (n = 6). (**C**) Tumor from the SAPCD2 knockdown group (sh) and corresponding control (NC). (**D**) Tumor volume measurements over time in the SAPCD2 knockdown and control groups. (**E**) Tumor weight comparison between the SAPCD2 knockdown and control groups at the experiment endpoint. (**F**) Images of mice bearing tumors from 5637 cells stably overexpressing SAPCD2 or vector control (n = 6). (**G**) Tumor images from the SAPCD2 overexpression group (OE) and control (Vec). (**H**) Tumor volume measurements in the SAPCD2 overexpression and control groups. (**I**) Tumor weight comparison between the SAPCD2 overexpression and control groups. (**J**) IHC staining for Ki-67 expression in tumors from the SAPCD2 knockdown group and corresponding control, overexpression group and corresponding control (Original magnification, ×20; scale bar = 50 μm). (**K**) In vivo imaging of metastatic burden in SAPCD2 knockdown and control mice (n = 6). *p* < 0.001 (***).

Then, the xenograft and metastasis models were used to assess the effects of SAPCD2 modulation on tumor growth and metastatic behavior in vivo. Subcutaneous xenograft models were established using BCa cells with stable SAPCD2 knockdown or overexpression, as outlined schematically (Figure 4A). A T24 cell line with stable SAPCD2 knockdown was generated using LV-shSAPCD2 (Figure S3I).

Tumors derived from SAPCD2-silenced T24 cells exhibited a reduced growth rate compared with control tumors following subcutaneous implantation (Figure 4B,C). Consistently, tumor volume and tumor weight were lower in the SAPCD2 knockdown group at the experimental endpoint (Figure 4D,E).

In contrast, tumors generated from SAPCD2-overexpressing 5637 cells displayed increased growth compared with their corresponding control tumors (Figure 4F,G). Quantitative analysis indicated higher tumor volume and tumor weight in the SAPCD2 overexpression group (Figure 4H,I). Histological analysis further supported these observations. Immunohistochemical staining indicated reduced Ki-67 positivity in tumors derived from SAPCD2-depleted cells, whereas higher Ki-67 staining was observed in SAPCD2-overexpressing tumors (Figure 4J).

In addition, the effects of SAPCD2 modulation on metastatic behavior were examined using in vivo metastasis models. SAPCD2 knockdown was associated with a reduced metastatic burden compared with control groups, as assessed by bioluminescence imaging and histological analysis (Figure 4K).

### 3.5. SAPCD2 Regulates Malignant Behaviors of BCa Cells Through MAPK Signaling

**Figure 5:**
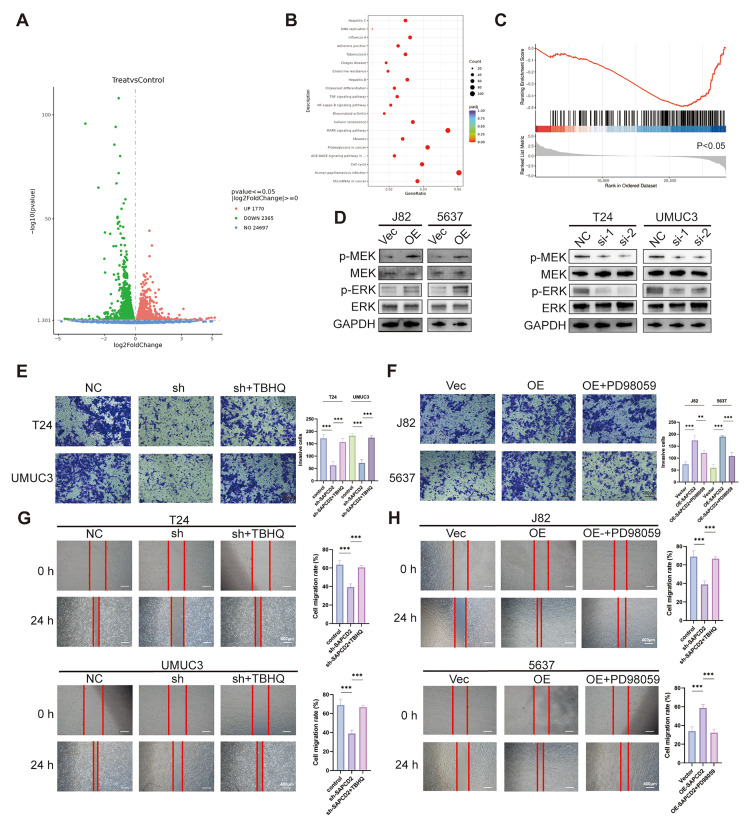
SAPCD2 Regulates Malignant Behaviors of Bladder Cancer Cells Through MAPK Signaling. (**A**) Volcano plot from transcriptomic profiling of T24 cells with SAPCD2 knockdown (si-SAPCD2) compared to control cells (si-NC). Differentially expressed genes were highlighted. (**B**) Gene Ontology (GO) analysis showing biological processes enriched in the SAPCD2 knockdown group, including cell cycle progression, wound healing, and cell adhesion. (**C**) Gene Set Enrichment Analysis (GSEA) result. (**D**) Level of phosphorylated MEK and ERK (p-MEK, p-ERK) and total MEK and ERK in bladder cancer cells. (**E**) Transwell invasion assay showing the effect of TBHQ on cell migration in SAPCD2 knockdown cells (Original magnification, ×10; scale bar = 200 μm). (**F**) Transwell invasion assay examining the effect of PD98059 on migration in SAPCD2-overexpressing cells (Original magnification, ×10; scale bar = 200 μm). (**G**) Wound healing assay assessing cell migration at 0 and 24 h in T24 and UMUC3 cells treated with TBHQ. (**H**) Wound healing assay assessing cell migration at 0 and 24 h in J82 and 5637 cells overexpressing SAPCD2, with and without PD98059 treatment. The red lines represent the edge of the cell scratch. (Original magnification, ×4; scale bar = 400 μm). *p* < 0.01 (**), *p* < 0.001 (***). The uncropped blots are shown in File S1.

Transcriptomic profiling was performed following siRNA-mediated knockdown of SAPCD2 in T24 cells. A distinct set of differentially expressed genes (DEGs) was identified after SAPCD2 silencing (Figure 5A). GO enrichment analysis revealed that these DEGs were enriched in biological processes related to wound healing, cell cycle regulation, and cell adhesion (Figure S4A). DisGeNET enrichment analysis further highlighted links between SAPCD2-related gene signatures and tumor-associated terms, including recurrent tumor and tumor angiogenesis (Figure S4B).

KEGG enrichment analysis identified MAPK, NF-κB, and TNF signaling pathways as prominently enriched among SAPCD2-associated DEGs (Figure 5B). GSEA further revealed differential enrichment of genes involved in MAPK signaling following SAPCD2 knockdown (Figure 5C). In line with these transcriptomic findings, reduced phosphorylation of MEK and ERK was observed after SAPCD2 knockdown, whereas increased MEK and ERK phosphorylation was detected in cells overexpressing SAPCD2, while total MEK and ERK protein levels remained largely unchanged (Figure 5D).

To further examine the functional relevance of MAPK signaling in SAPCD2-modified BCa cells, pharmacological modulation of the MAPK pathway was performed. Treatment with the MAPK pathway agonist tert-butylhydroquinone (TBHQ) coincided with increased migratory and invasive capacities in SAPCD2-depleted cells, as assessed by Transwell invasion assays (Figure 5E) and wound healing assays (Figure 5G). In parallel, alterations in EMT-related marker expression were detected following TBHQ treatment in SAPCD2-silenced cells (Figure S4C).

Conversely, treatment with the MAPK pathway inhibitor PD98059 corresponded to reduced migratory and invasive capacities in SAPCD2-overexpressing cells, as evaluated by Transwell invasion assays (Figure 5F) and wound healing assays (Figure 5H). PD98059 treatment was also accompanied by corresponding changes in EMT-related marker expression in these cells (Figure S4D).

### 3.6. Identification of TANK as a SAPCD2-Interacting Protein

**Figure 6:**
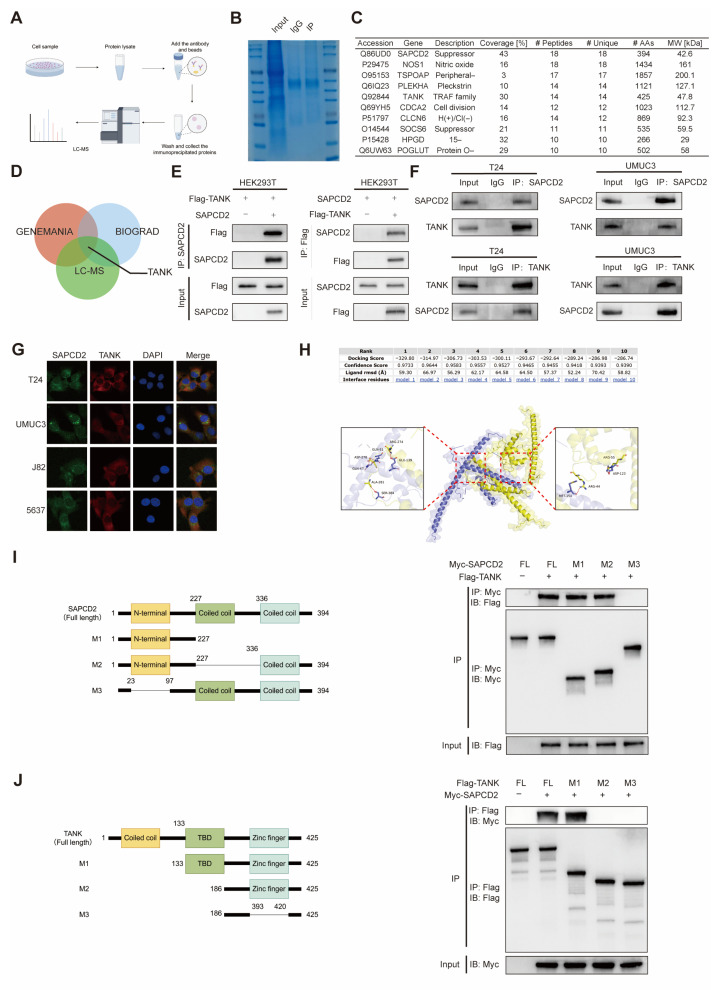
Identification of TANK as a SAPCD2-Interacting Protein. (**A**) Schematic of the Co-IP/MS workflow used to identify SAPCD2-associated proteins. (**B**) Coomassie brilliant blue staining of proteins enriched by Co-IP with SAPCD2. (**C**) Top 10 SAPCD2-interacting proteins based on unique peptide counts identified through mass spectrometry. (**D**) Protein–protein interaction network of SAPCD2-derived candidates, analyzed using BioGRID and GeneMANIA databases. (**E**) Exogenous Co-IP assays validating the interaction between SAPCD2 and TANK. (**F**) Endogenous Co-IP assays confirming the interaction between SAPCD2 and TANK in bladder cancer cells. (**G**) Confocal immunofluorescence showing co-localization of SAPCD2 and TANK in four bladder cancer cell lines. SAPCD2 was labeled with FITC (Green), TANK was labeled with Cy3 (Red), and cell nuclei were counterstained with DAPI (Blue). (**H**) Molecular docking analysis of SAPCD2 (Yellow) and TANK (Purple). (**I**) The regions essential for their interaction of SAPCD2. (**J**) The regions essential for their interaction of TANK. The uncropped blots are shown in File S1.

To identify proteins potentially involved in SAPCD2-related MAPK regulation, a co-immunoprecipitation (Co-IP) coupled with mass spectrometry approach was performed, as schematically illustrated (Figure 6A). Proteins enriched in SAPCD2 immunoprecipitates were visualized by Coomassie brilliant blue staining (Figure 6B) and subsequently identified by mass spectrometry (Table S6). Among the identified candidates, the top 10 proteins ranked by unique peptide counts are summarized in Figure 6C.

To further refine potential interaction partners, the MS-derived candidates were compared with protein–protein interaction predictions from the BioGRID and GeneMANIA databases. TANK was the only protein present in all three datasets (Figure 6D). Given that TANK has been previously implicated in MAPK pathway regulation, this convergence strongly suggested that TANK might function as a critical mediator of SAPCD2-driven MAPK signaling [17,18]. The interaction between SAPCD2 and TANK was subsequently examined by exogenous co-immunoprecipitation assays (Figure 6E) and further evaluated under endogenous conditions (Figure 6F and Figure S5A). In addition, confocal immunofluorescence microscopy revealed co-localization of SAPCD2 and TANK in four BCa cell lines (Figure 6G).

To explore the structural features of this interaction, molecular docking analyses were performed. Multiple potential binding conformations between SAPCD2 and TANK were identified, with the top-ranked docking model yielding a docking score of −329.8 (Figure 6H). Furthermore, co-immunoprecipitation assays using purified recombinant proteins were conducted to map the interaction domains. These analyses identified the N-terminal region of SAPCD2 (amino acids 23–97) and the TANK-binding domain (TBD; amino acids 133–186) of TANK as regions involved in mediating their association (Figure 6I,J).

### 3.7. SAPCD2 Stabilizes TANK by Inhibiting SYVN1-Mediated Ubiquitination and Proteasomal Degradation

**Figure 7:**
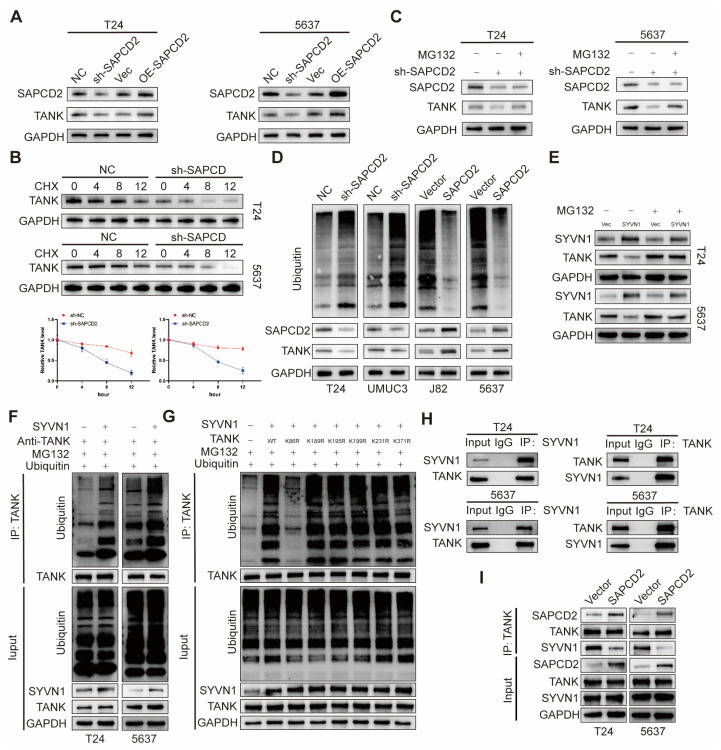
SAPCD2 Regulates TANK Stability Through Proteasome-Dependent Degradation. (**A**) SAPCD2 and TANK expression were positively correlated in bladder cancer cells. (**B**) TANK degradation was accelerated in SAPCD2-depleted T24 and 5637 cells following CHX treatment. (**C**) Restoration of TANK levels in SAPCD2-depleted cells after MG132 treatment, indicating proteasome-dependent degradation. (**D**) Increased polyubiquitination of TANK in SAPCD2-silenced cells, while overexpression of SAPCD2 reduced TANK polyubiquitination. (**E**) SYVN1 overexpression reduced TANK protein levels, with MG132 treatment preventing this effect. (**F**) SYVN1 induced polyubiquitination of TANK in MG132-treated cells. (**G**) Mutagenesis of TANK showing that the K86R mutation impairs SYVN1-mediated ubiquitination of TANK. (**H**) Interaction between SYVN1 and TANK in T24 and 5637 cells, confirming their physical association. (**I**) Overexpression of SAPCD2 reduced the interaction between SYVN1 and TANK and stabilized TANK protein levels. The uncropped blots are shown in File S1.

The relationship between SAPCD2 and TANK expression was next examined. A positive correlation between SAPCD2 expression and TANK protein levels was observed in BCa cells (Figure 7A). In contrast, modulation of SAPCD2 expression did not alter TANK mRNA levels (Figure S5B). To further evaluate whether SAPCD2 affects TANK protein stability, protein synthesis was inhibited using cycloheximide (CHX), and TANK protein levels were monitored over time. Following CHX treatment, TANK protein levels declined more rapidly in SAPCD2-depleted T24 and 5637 cells than in control cells (Figure 7B). Quantitative analysis demonstrated a reduced half-life of TANK protein in the absence of SAPCD2.

To assess the involvement of proteasomal degradation, cells were treated with the proteasome inhibitor MG132. MG132 treatment restored TANK protein levels in SAPCD2-depleted cells (Figure 7C). In parallel, ubiquitination assays were performed to examine changes in TANK ubiquitination. Increased accumulation of polyubiquitinated TANK was detected following SAPCD2 knockdown, whereas reduced TANK polyubiquitination was observed in SAPCD2-overexpressing cells across multiple BCa cell lines (Figure 7D).

To identify E3 ubiquitin ligases potentially involved in TANK ubiquitination, in silico prediction was performed using the UbiBrowser database, which identified SYVN1 as a candidate. Overexpression of SYVN1 resulted in reduced TANK protein levels, and this effect was attenuated by MG132 treatment (Figure 7E). In addition, SYVN1 overexpression led to increased polyubiquitination of TANK in MG132-treated cells (Figure 7F).

To further map ubiquitination sites on TANK, several lysine residues predicted by UbiBrowser (K86, K189, K195, K199, K231, and K371) were individually mutated to arginine. Among these mutants, the K86R substitution markedly reduced SYVN1-mediated ubiquitination of TANK (Figure 7G). Co-immunoprecipitation assays further detected an interaction between SYVN1 and TANK in T24 and 5637 BCa cells (Figure 7H). Notably, SAPCD2 overexpression reduced the extent of the SYVN1–TANK interaction and was accompanied by increased TANK protein levels (Figure 7I).

### 3.8. SAPCD2-Induced Malignant Behaviors Are Mediated by the TANK–MAPK Axis

**Figure 8:**
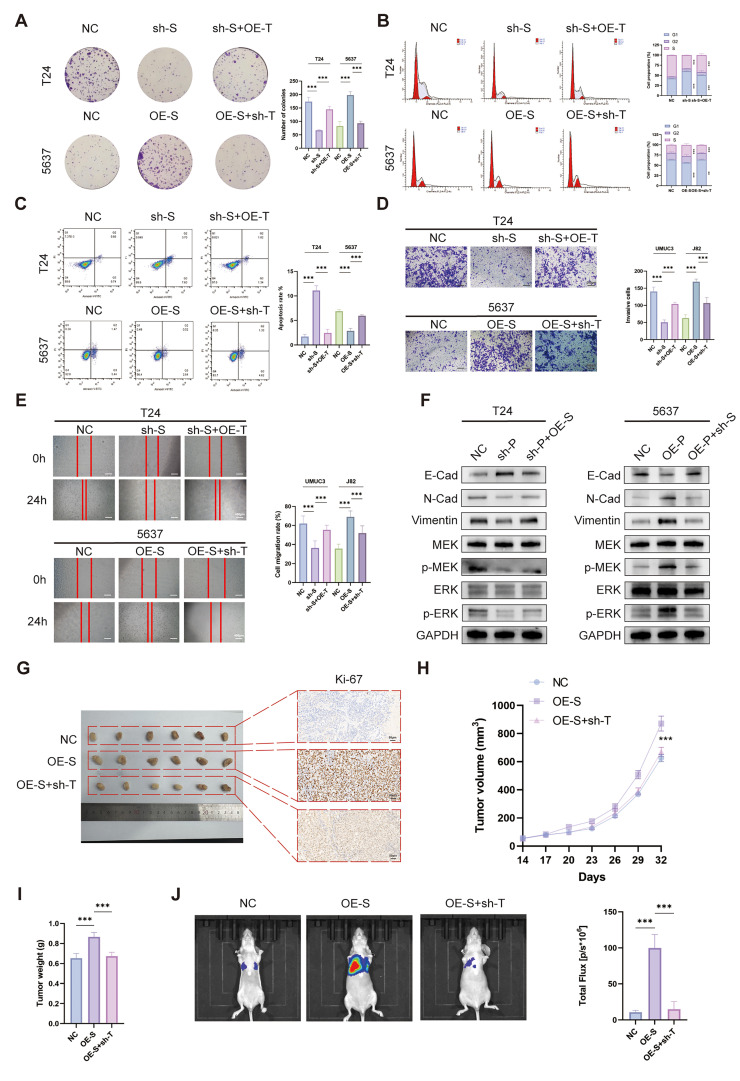
SAPCD2-Induced Malignant Behaviors Are Mediated by the TANK–MAPK Axis. (**A**) Re-expression of TANK rescued the reduced colony formation caused by SAPCD2 knockdown, while TANK depletion diminished the clonogenic growth induced by SAPCD2 overexpression. (**B**) Cell cycle progression was reversed upon TANK depletion in SAPCD2-overexpressing cells, and TANK re-expression alleviated G1-phase accumulation in SAPCD2-depleted cells. (**C**) Apoptosis analysis showed that TANK re-expression mitigated apoptosis in SAPCD2-depleted cells, whereas TANK depletion promoted apoptosis in SAPCD2-overexpressing cells. (**D**) TANK re-expression restores the invasive capacity of SAPCD2-depleted cells, and TANK silencing weakened the invasiveness induced by SAPCD2 overexpression (Original magnification, ×10; scale bar = 200 μm). (**E**) Changes in migration were reversed by modulation of TANK expression, with TANK depletion reducing migration in SAPCD2-overexpressing cells. The red lines represent the edge of the cell scratch (Original magnification, ×4; scale bar = 400 μm). (**F**) MAPK signaling makers were reduced upon TANK depletion in SAPCD2-silenced cells and restored with TANK re-expression in SAPCD2 overexpression cells. (**G**) Tumor growth and ki67 staining in xenograft models were significantly reversed by TANK depletion in SAPCD2-overexpressing tumors (n = 6; Original magnification, ×20; scale bar = 50 μm). (**H**) Tumor volume and (**I**) tumor weight were both reduced in the TANK-depleted group compared to the SAPCD2-overexpressing group. (**J**) TANK knockdown prevented lung metastasis of tumors driven by SAPCD2 overexpression (n = 6). *p* < 0.01 (**) and *p* < 0.001 (***). The uncropped blots are shown in File S1.

The effects of TANK modulation were next examined in the context of SAPCD2 gain- or loss-of-function. Re-expression of TANK increased colony formation in SAPCD2-depleted cells, whereas TANK knockdown reduced clonogenic growth in SAPCD2-overexpressing cells (Figure 8A). Similar patterns were observed in analyses of cell cycle distribution and apoptosis. Reduction in TANK expression was accompanied by altered cell cycle profiles and increased apoptosis in SAPCD2-overexpressing cells, while enforced TANK expression was accompanied by changes in G1-phase distribution and apoptotic rates in SAPCD2-silenced cells (Figure 8B,C).

Comparable effects were observed in assays evaluating cell migration and invasion. Re-expression of TANK increased the invasive capacity of SAPCD2-depleted BCa cells, whereas TANK silencing reduced invasion in SAPCD2-overexpressing cells (Figure 8D). Changes in migratory behavior following SAPCD2 manipulation were also modulated by corresponding alterations in TANK expression (Figure 8E).

At the molecular level, modulation of TANK expression altered the expression patterns of EMT-related markers in SAPCD2-modified cells (Figure 8F). In addition, changes in TANK expression were accompanied by corresponding alterations in MAPK pathway activity in the context of SAPCD2 gain or loss (Figure 8F).

In xenograft models, modulation of TANK expression influenced tumor growth in SAPCD2-modified cells (Figure 8G). Increased tumor volume and tumor weight observed in SAPCD2-overexpressing tumors were reduced following TANK knockdown (Figure 8H,I). Furthermore, TANK depletion was accompanied by a reduction in lung metastatic burden in models with SAPCD2 overexpression (Figure 8J).

### 3.9. PLAGL2–SAPCD2–TANK–MAPK Signaling Drives Malignant Behaviors in BCa Through CREB Activation and Positive Feedback Loop

To examine transcriptional regulators of SAPCD2, the JASPAR database was used to predict transcription factors with potential binding sites in the SAPCD2 promoter. Among the predicted candidates, PLAGL2 was upregulated in BCa samples and its expression levels varied with overall survival and disease-free survival in public datasets (Figure S6A–C). In addition, a positive correlation between PLAGL2 and SAPCD2 expression was observed in BCa samples (Figure S6D). Analysis of BCa cell lines and tissue samples further demonstrated higher PLAGL2 expression compared with normal urothelial controls (Figure S6E–G).

**Figure 9:**
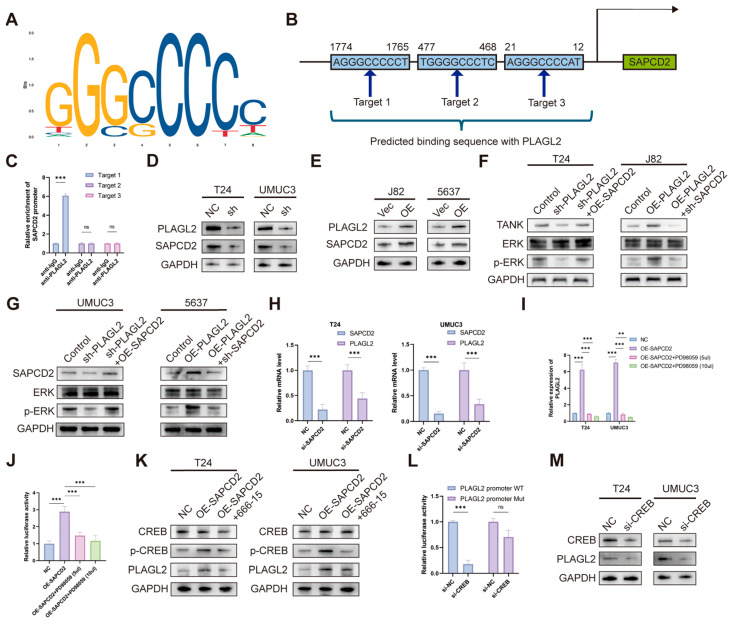
PLAGL2–SAPCD2–TANK–MAPK Signaling Drives Malignant Behaviors in Bladder Cancer Through CREB Activation and Positive Feedback Loop. (**A**) Predicted binding sites for PLAGL2 on the SAPCD2 promoter region, identified using the JASPAR database. (**B**) Schematic of the three highly probable PLAGL2 binding sites. (**C**) ChIP demonstrating PLAGL2 enrichment at the SAPCD2 promoter binding site 1. (**D**,**E**) PLAGL2 alteration changed SAPCD2 protein levels in bladder cancer cells. (**F**,**G**) SAPCD2 rescued the changes in MAPK signaling and TANK expression induced by PLAGL2. (**H**) mRNA levels of PLAGL2 were reduced after SAPCD2 knockdown. (**I**) PLAGL2 expression is inhibited by the MAPK signaling inhibitor PD98059. (**J**) PD98059 reversed the increase in PLAGL2 promoter activity caused by overexpression of SAPCD2. (**K**) PD98059 reversed the increase in CREB phosphorylation caused by overexpression of SAPCD2. (**L**) PLAGL2 promoter activity in cells with CREB silencing. (**M**) Reduced PLAGL2 protein levels upon CREB silencing in T24 and UMUC3 cells. Not significant (ns), *p* < 0.01 (**) and *p* < 0.001 (***). The uncropped blots are shown in File S1.

To assess whether PLAGL2 influences SAPCD2 transcriptional activity, dual-luciferase reporter assays were performed. PLAGL2 depletion reduced luciferase activity driven by the wild-type SAPCD2 promoter, whereas no change was detected with the mutant promoter construct (Figure S6H). JASPAR analysis further identified three putative PLAGL2-binding sites within the SAPCD2 promoter region (Figure 9A,B). Chromatin immunoprecipitation assays detected enrichment of the SAPCD2 promoter at binding site 1 following anti-PLAGL2 immunoprecipitation (Figure 9C).

Modulation of PLAGL2 expression altered SAPCD2 expression at both the mRNA (Figure S6I,J) and protein levels (Figure 9D,E). In parallel, changes in PLAGL2 expression were accompanied by corresponding alterations in TANK protein abundance and ERK phosphorylation, which were attenuated upon SAPCD2 silencing or enhanced upon enforced SAPCD2 expression (Figure 9F,G).

Interestingly, we found that after knockdown of SAPCD2, the mRNA expression of PLAGL2 also decreased (Figure 9H). We boldly speculated that the downstream of SAPCD2 could regulate the upstream of PLAGL2, thereby forming a positive feedback pathway. To explore this further, we used bioinformatic tools such as hTFtarget, GeneCards, and JASPAR to predict upstream transcription factors of PLAGL2, identifying 13 potential upstream transcription factors of PLAGL2 (Figure S7A). Previous studies have established that MAPK activation can induce the phosphorylation of downstream transcription factors such as CREB and FOS, thereby enhancing the transcriptional activity of a variety of genes [19]. Notably, CREB was among the candidate upstream transcription factors of PLAGL2 identified through our integrative database analyses. This prompted us to consider whether MAPK activation might regulate PLAGL2 expression through ERK-dependent CREB phosphorylation.

Consistent with this possibility, inhibition of MAPK signaling with the MEK inhibitor PD98059 effectively reversed the increase in PLAGL2 expression induced by SAPCD2 (Figure 9I). In parallel, PD98059 reversed the increase in PLAGL2 promoter activity and CREB phosphorylation caused by overexpression of SAPCD2 (Figure 9J,K).

To examine whether CREB influenced PLAGL2 transcriptional activity, dual-luciferase reporter assays were conducted. CREB overexpression increased PLAGL2 promoter activity (Figure 9L). Conversely, CREB silencing reduced PLAGL2 expression at both the mRNA (Figure S7B,C) and protein levels (Figure 9M).

## 4. Discussion

In this study, we provided evidence supporting a role for SAPCD2 in BCa progression and proposed a multilayered regulatory network through which SAPCD2 might contribute to sustained malignant signaling. Our data indicated that SAPCD2 was frequently upregulated in BCa, was associated with aggressive clinicopathological features and unfavorable prognosis, and influenced tumor growth and metastatic behavior in experimental models. Beyond its potential clinical relevance, our findings suggested that SAPCD2 participated in the integration of post-translational regulation, oncogenic signaling, and transcriptional feedback mechanisms converging on MAPK pathway activity in BCa.

SAPCD2 upregulation has been reported in multiple malignancies and is commonly associated with enhanced proliferation, invasion, and poor clinical outcomes [20,21]. Previous mechanistic studies have primarily linked SAPCD2 to mitotic regulation and chromosomal stability, as well as to signaling pathways such as Wnt/β-catenin [15]. Our results extended these observations by suggesting that, in BCa, SAPCD2 was closely connected with MAPK signaling. This finding was consistent with a context-dependent model in which SAPCD2 engaged distinct downstream pathways depending on tumor type and cellular background.

Although modulation of SAPCD2 expression produced pronounced effects on cell cycle progression, survival, and metastatic phenotypes, the significance of these observations lay in their convergence on pathway-level dependency. Transcriptomic profiling, pathway enrichment analyses, and pharmacological modulation collectively pointed to MAPK signaling as a major pathway associated with SAPCD2 activity in BCa models. Notably, altering MAPK pathway activity substantially modified the phenotypic consequences of SAPCD2 gain or loss, supporting a model in which SAPCD2 influenced malignant behavior at least in part through MAPK-related signaling.

An important mechanistic aspect of this study was the identification of TANK as an interaction partner linking SAPCD2 to MAPK pathway regulation. TANK has been characterized as a scaffold protein involved in integrating TNF, NF-κB, and MAPK signaling; however, the regulation of its protein stability in cancer contexts has remained incompletely understood [18]. Our data suggested that SAPCD2 associated with TANK and modulates its abundance, and that genetic manipulation of TANK alters MAPK activity and malignant phenotypes in SAPCD2-modified models. Together, these observations supported the notion that TANK might function as an intermediary through which SAPCD2 influenced MAPK signaling.

Further analyses suggested that SAPCD2 affected TANK stability by interfering with SYVN1-dependent ubiquitination. SYVN1 is an ER-resident E3 ubiquitin ligase that has been implicated in the regulation of multiple signaling pathways [22,23,24]. Our findings showed that SYVN1 directly ubiquitinated TANK at lysine 86, promoting its proteasomal degradation, and that SAPCD2 interfered with this process by limiting the interaction between SYVN1 and TANK. As a result, SAPCD2 enabled sustained accumulation of TANK and prolonged MAPK pathway activation. Unlike typical transient signaling, where pathways were tightly regulated, this mode of regulation emphasizes how SAPCD2 reprograms the cellular proteostasis machinery to maintain prolonged signaling. By stabilizing TANK, SAPCD2 ensured continuous MAPK activation, promoting the persistent malignant behaviors seen in BCa cells

Upstream of SAPCD2, we identified PLAGL2 as a direct transcriptional activator that binds the SAPCD2 promoter and enhances its expression. PLAGL2 has recently emerged as an oncogenic transcription factor capable of coordinating tumor-intrinsic programs with microenvironmental remodeling [25,26]. Our findings extended its functional repertoire by establishing SAPCD2 as a critical PLAGL2 target in BCa. Notably, SAPCD2 did not merely act downstream of PLAGL2 but fed back to reinforce PLAGL2 expression through MAPK-dependent CREB phosphorylation. In this circuit, SAPCD2 enhanced MAPK signaling, leading to CREB activation, which in turn drives PLAGL2 transcription; elevated PLAGL2 then further promoted SAPCD2 expression. This closed-loop architecture provides a mechanistic explanation for the sustained and self-amplifying signaling observed in SAPCD2-high tumors and illustrates how transcriptional and post-translational regulation converge to stabilize oncogenic states.

Such positive feedback loops have been implicated in maintaining sustained oncogenic signaling and promoting phenotypic stability in cancer cells, often buffering against perturbations from upstream inhibitors and contributing to aggressive tumor behavior [27,28]. the multi-layered feedback circuitry identified here suggests that targeting a single pathway component may be insufficient for durable therapeutic benefit. Future studies could explore combination strategies aimed at disrupting this network at multiple nodes, such as concurrently modulating MAPK signaling, transcriptional regulators (e.g., PLAGL2 or CREB), and protein stability mechanisms affecting TANK. Such approaches may help overcome pathway redundancy and adaptive resistance.

Several limitations of this study warranted careful consideration. First, much of the mechanistic work was performed using established BCa cell lines, which might not fully capture the molecular heterogeneity of primary tumors. Second, the number of human BCa tissue samples analyzed was relatively limited, which might restrict the generalizability of the clinical correlations observed. Third, the in vivo experiments relied primarily on xenograft models in immunodeficient mice, which did not recapitulate the complexity of tumor–immune interactions. Given the known roles of MAPK signaling and PLAGL2 in immunomodulation, future studies using immunocompetent or patient-derived models will be important to more fully define the biological relevance of this signaling axis.

## 5. Conclusions

In summary, our findings suggested a role for SAPCD2 in BCa progression and indicated that SAPCD2 could contributed to malignant phenotypes through a regulatory network involving TANK stabilization, MAPK pathway activity, and PLAGL2–CREB–mediated transcriptional feedback. This signaling axis may represent a mechanism by which post-translational regulation, signal amplification, and transcriptional control are coordinated in BCa. Although these results provided insight into the potential biological significance of SAPCD2, further studies using larger and more comprehensively stratified tumor cohorts will be required to confirm and extend these observations and to better define their clinical relevance.

## Figures and Tables

**Table 1:** Clinical information of patients in our center.

Characteristics	Number	Expression of SAPCD2		*p* Value
	71	High (n = 42)	Low (n = 29)	
Age				0.489
≥60	45	28	17	
<60	28	14	12	
Gender				0.823
Male	50	30	20	
Female	21	12	9	
Tumor grade				0.021
Low	19	7	12	
High	52	35	17	
T stage				0.047
Ta+T1	34	16	18	
T2-4	37	26	11	
N stage				0.558
N0	63	36	27	
N1	8	6	2	
M stage				0.122
M0	59	32	27	
M1	12	10	2	

## Data Availability

Public data from TCGA (https://portal.gdc.cancer.gov/) and GEO (https://www.ncbi.nlm.nih.gov/geo/) databases were analyzed in this research. The experimental data for this study are available from the corresponding author upon reasonable request.

## Supplementary Materials

The following supporting information can be downloaded at: https://www.mdpi.com/article/10.3390/cancers18030535/s1, Figure S1: SAPCD2 Expression in Normal Human Tissues and Bladder Cancer; Figure S2: SAPCD2 Expression and Its Impact on Survival in Multiple Cancer Types; Figure S3: SAPCD2 Regulates Cell Proliferation in Bladder Cancer; Figure S4: Gene Enrichment Analysis and EMT Regulation by SAPCD2 in Bladder Cancer Cells; Figure S5: Interaction Between SAPCD2 and TANK, and Impact on TANK mRNA Expression; Figure S6: Upstream Regulation of SAPCD2 by PLAGL2 in Bladder Cancer; Figure S7: Bioinformatic Prediction and CREB Regulation of PLAGL2 Expression; Table S1: PCR primers sequence; Table S2: Antibody catalog numbers and dilutions for WB; Table S3: The sequences of the siRNAs; Table S4: The clinical information of the patients The sequences of the shRNAs; Table S5: The clinical information of the patients; Table S6: LC/MS results. File S1: Western blots original images.

## Abbreviations

The following abbreviations are used in this manuscript:

BCa	Bladder cancer
SAPCD2	Suppressor anaphase-promoting complex domain containing 2
TANK	TRAF associated NF-kB activator
SYVN1	Synoviolin
PLAGL2	Pleomorphic adenoma gene-like 2
qRT-PCR	Quantitative reverse transcription polymerase chain reaction
TCGA	The Cancer Genome Atlas
GEO	Gene Expression Omnibus
GO	Gene Ontology
KEGG	Kyoto Encyclopedia of Genes and Genomes
OS	Overall Survival
DFS	Disease-Free Survival
CCK-8	Cell Counting Kit-8
ChIP	Chromatin Immunoprecipitation
CO-IP	Co-Immunoprecipitation
HPA	Human Protein Atlas
WB	Western blot
IHC	Immunohistochemistry
PI	Propidium iodide
DEGs	Differentially expressed genes
TBHQ	tert-butylhydroquinone

## References

[1] Jubber I., Ong S., Bukavina L., Black P.C., Compérat E., Kamat A.M., Kiemeney L., Lawrentschuk N., Lerner S.P., Meeks J.J. (2023). Epidemiology of Bladder Cancer in 2023: A Systematic Review of Risk Factors. Eur. Urol..

[2] Siegel R.L., Miller K.D., Jemal A. (2018). Cancer statistics, 2018. CA Cancer J. Clin..

[3] Rubin J.B., Lagas J.S., Broestl L., Sponagel J., Rockwell N., Rhee G., Rosen S.F., Chen S., Klein R.S., Imoukhuede P. (2020). Sex differences in cancer mechanisms. Biol. Sex Differ..

[4] Lenis A.T., Lec P.M., Chamie K., Mshs M.D. (2020). Bladder Cancer: A Review. JAMA.

[5] Cumberbatch M.G.K., Jubber I., Black P.C., Esperto F., Figueroa J.D., Kamat A.M., Kiemeney L., Lotan Y., Pang K., Silverman D.T. (2018). Epidemiology of Bladder Cancer: A Systematic Review and Contemporary Update of Risk Factors in 2018. Eur. Urol..

[6] Xu Y., Luo C., Wang J., Chen L., Chen J., Chen T., Zeng Q. (2021). Application of nanotechnology in the diagnosis and treatment of bladder cancer. J. Nanobiotechnol..

[7] Patel V.G., Oh W.K., Galsky M.D. (2020). Treatment of muscle-invasive and advanced bladder cancer in 2020. CA Cancer J. Clin..

[8] Seidl C. (2020). Targets for Therapy of Bladder Cancer. Semin. Nucl. Med..

[9] Heard J.R., Ahdoot M., Theodorescu D., Mitra A.P. (2024). Biomarkers of treatment response in bladder cancer. Expert Rev. Mol. Diagn..

[10] Baker A.L., Du L. (2022). The Function and Regulation of SAPCD2 in Physiological and Oncogenic Processes. J. Cancer.

[11] Chiu C.W.N., Monat C., Robitaille M., Lacomme M., Daulat A.M., Macleod G., McNeill H., Cayouette M., Angers S. (2016). SAPCD2 Controls Spindle Orientation and Asymmetric Divisions by Negatively Regulating the Gαi-LGN-NuMA Ternary Complex. Dev. Cell.

[12] Zhang Y., Liu J.L., Wang J. (2020). SAPCD2 promotes invasiveness and migration ability of breast cancer cells via YAP/TAZ. Eur. Rev. Med. Pharmacol. Sci..

[13] Zhu B., Wu Y., Niu L., Yao W., Xue M., Wang H., Yang J., Li J., Fan W. (2020). Silencing SAPCD2 Represses Proliferation and Lung Metastasis of Fibrosarcoma by Activating Hippo Signaling Pathway. Front. Oncol..

[14] Luo Y., Wang L., Ran W., Li G., Xiao Y., Wang X., Zhao H., Xing X. (2020). Overexpression of SAPCD2 correlates with proliferation and invasion of colorectal carcinoma cells. Cancer Cell Int..

[15] Jiang J., Tang S., Xia J., Wen J., Chen S., Shu X., Huen M.S.Y., Deng Y. (2018). C9orf140, a novel Axin1-interacting protein, mediates the negative feedback loop of Wnt/β-catenin signaling. Oncogene.

[16] Liu H., Zhu M., Li Z., Wang Y., Xing R., Lu Y., Xue W. (2017). Depletion of p42.3 gene inhibits proliferation and invasion in melanoma cells. J. Cancer Res. Clin. Oncol..

[17] Stellzig J., Chariot A., Shostak K., Ismail Göktuna S., Renner F., Acker T., Pagenstecher A., Schmitz M.L. (2013). Deregulated expression of TANK in glioblastomas triggers pro-tumorigenic ERK1/2 and AKT signaling pathways. Oncogenesis.

[18] Shi J.H., Sun S.C. (2018). Tumor Necrosis Factor Receptor-Associated Factor Regulation of Nuclear Factor κB and Mitogen-Activated Protein Kinase Pathways. Front. Immunol..

[19] De Luca A., Maiello M.R., D’Alessio A., Pergameno M., Normanno N. (2012). The RAS/RAF/MEK/ERK and the PI3K/AKT signalling pathways: Role in cancer pathogenesis and implications for therapeutic approaches. Expert Opin. Ther. Targets.

[20] Liu Y., Li B., Ke L., Luo T., Wu H., Lin J., Deng Y., Huang X., Xu L., Liu Y. (2025). Comprehensive Bioinformatics Analyses and Experimental Validation of the Cell Cycle Related Protein SAPCD2 as a New Biomarker and Potential Therapeutic Target in Pancreatic Cancer. J. Inflamm. Res..

[21] Wei D. (2022). MiR-486-5p specifically suppresses SAPCD2 expression, which attenuates the aggressive phenotypes of lung adenocarcinoma cells. Histol. Histopathol..

[22] Zhu L., Liu Y.P., Yuan W., Sun B.X., Huang Y.T., Zhao J.K., Liu J.F., Yu L.M., Wang H.S. (2025). E3 ubiquitin ligase SYVN1 as a promising therapeutic target for diverse human diseases. Pharmacol. Res..

[23] Chen S., Zhang J., Sun D., Wu Y., Fang J., Wan X., Li S., Zhang S., Gu Q., Shao Q. (2023). SYVN1 Promotes STAT3 Protein Ubiquitination and Exerts Antiangiogenesis Effects in Retinopathy of Prematurity Development. Investig. Ophthalmol. Vis. Sci..

[24] Xie X., Tong W., Xie Y., Jiang H., Jiang A., Huang J., Liu Z., Yu J. (2025). Targeting the SYVN1-EGFR axis: A breakthrough strategy for TKI-resistant NSCLC. Cell Death Dis..

[25] Wu L., Zhao N., Zhou Z., Chen J., Han S., Zhang X., Bao H., Yuan W., Shu X. (2021). PLAGL2 promotes the proliferation and migration of gastric cancer cells via USP37-mediated deubiquitination of Snail1. Theranostics.

[26] Shen Y., Zhai D., Zhao W., Chen Y., Ni J., Li J., Wu Z., Xu Y., Li B., Zhuang S. (2025). PLAGL2 promotes HCC progression by recruiting tumour-associated macrophages via CCL2/CCR2 signalling. Br. J. Pharmacol..

[27] Brandman O., Meyer T. (2008). Feedback loops shape cellular signals in space and time. Science.

[28] Harris S.L., Levine A.J. (2005). The p53 pathway: Positive and negative feedback loops. Oncogene.

