# Supplementary material for: SAPCD2 Drives Bladder Cancer Progression by Stabilizing TANK and Engaging a CREB–PLAGL2 Feedback Loop to Sustain MAPK Signaling
Source: Cancers (Basel). 2026 Feb 6;18(3):535. doi: 10.3390/cancers18030535 (PMC12896413; doi:10.3390/cancers18030535)

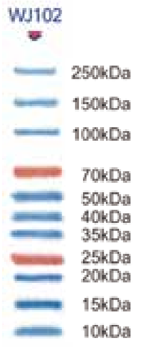

This study utilized the WJ102 product from Epizyme Biotech，China as the western blot marker.

Figure 1E

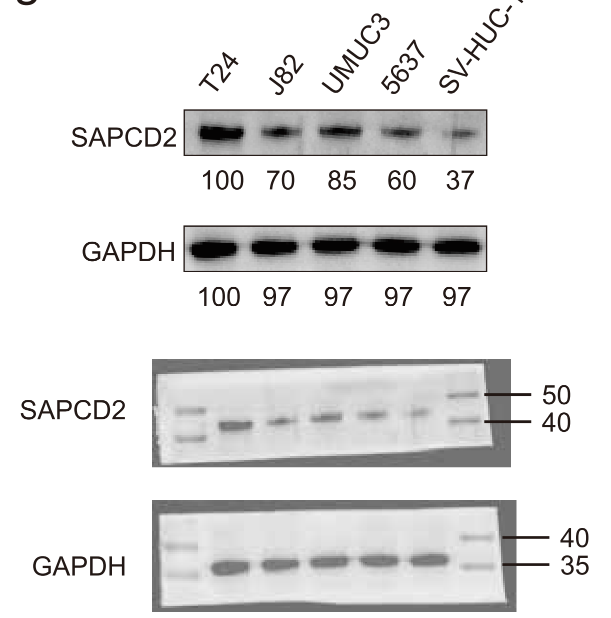

Figure 1F

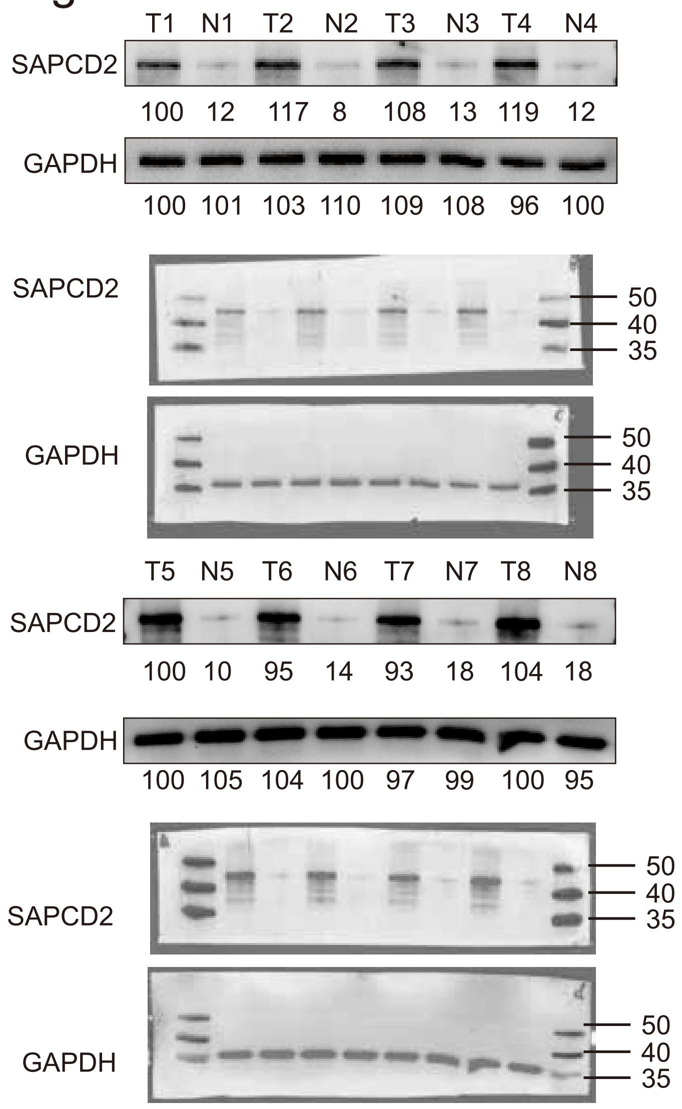

Figure 2A

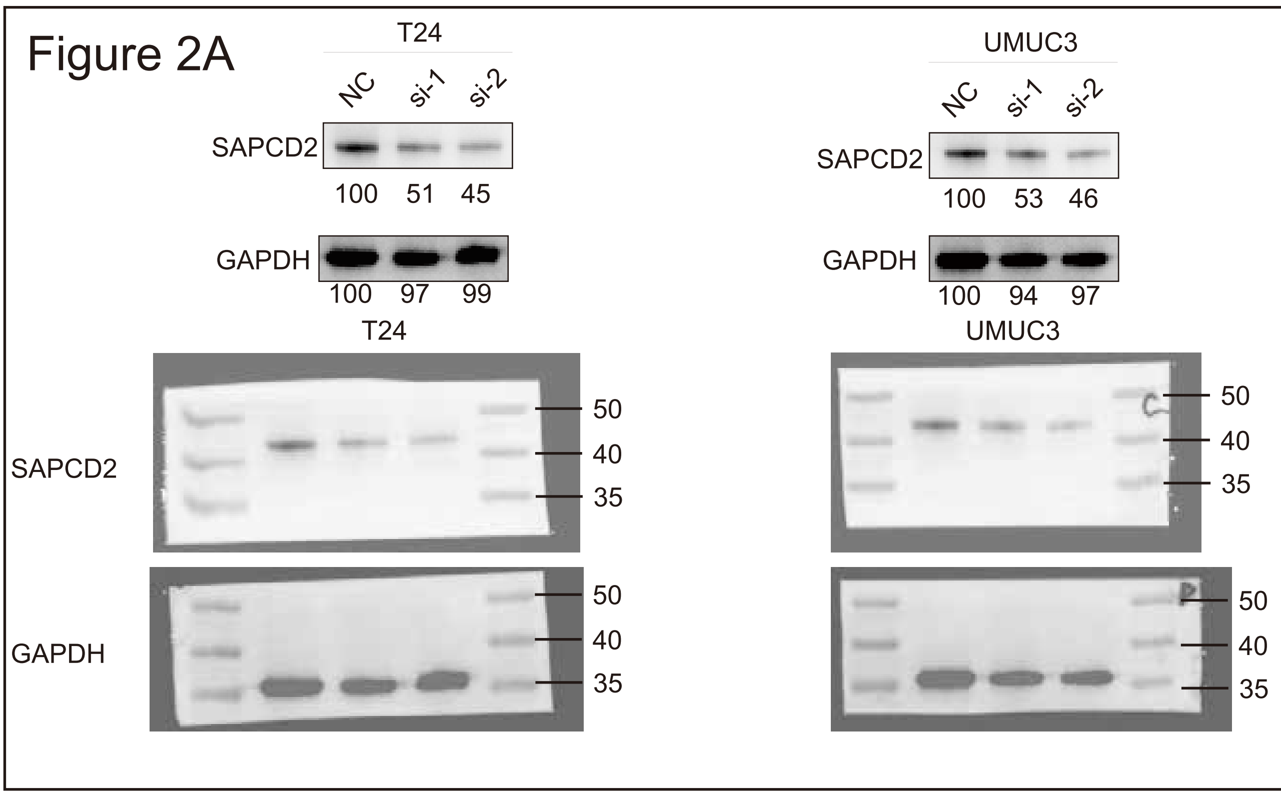

Figure 2I

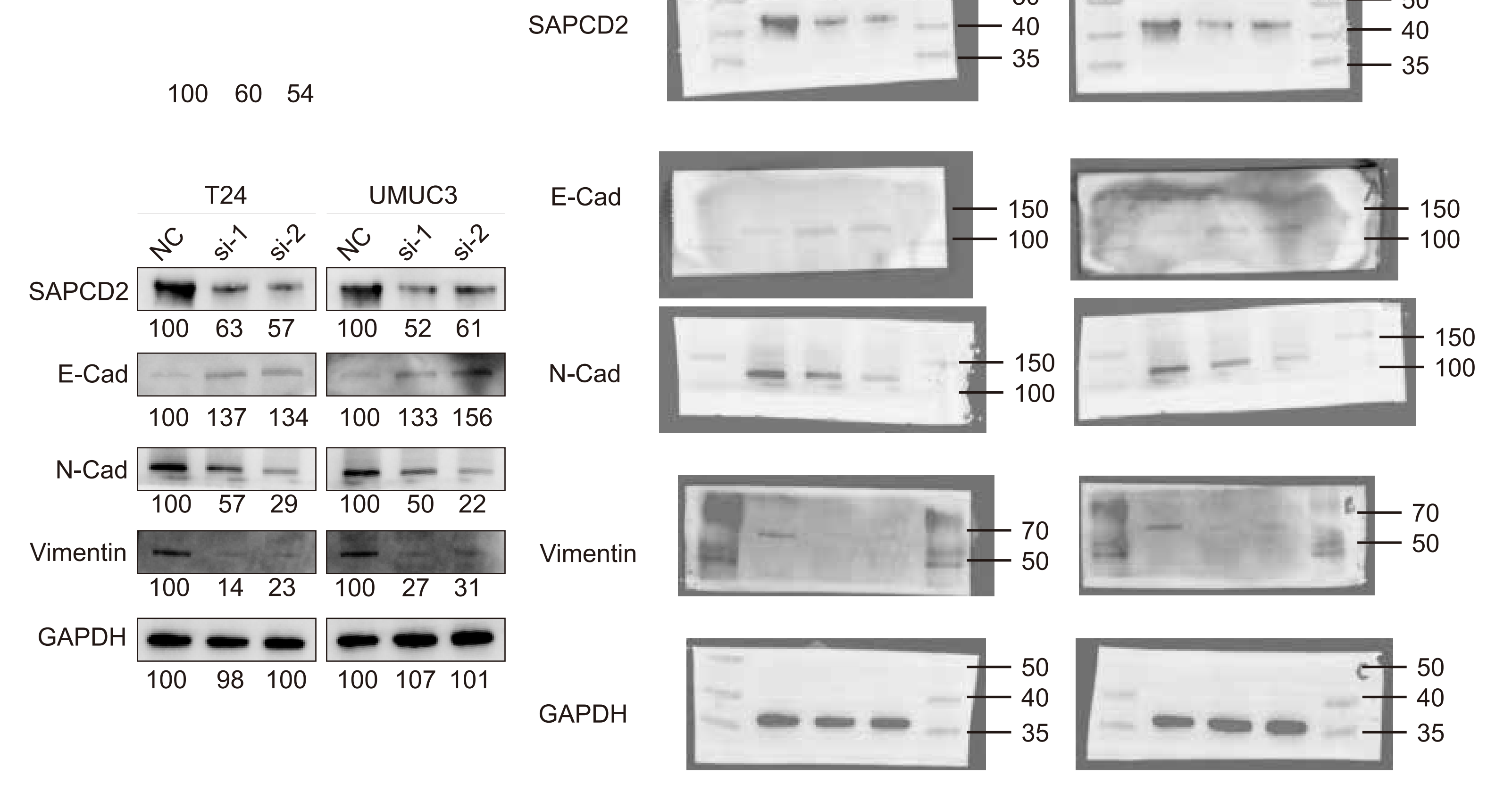

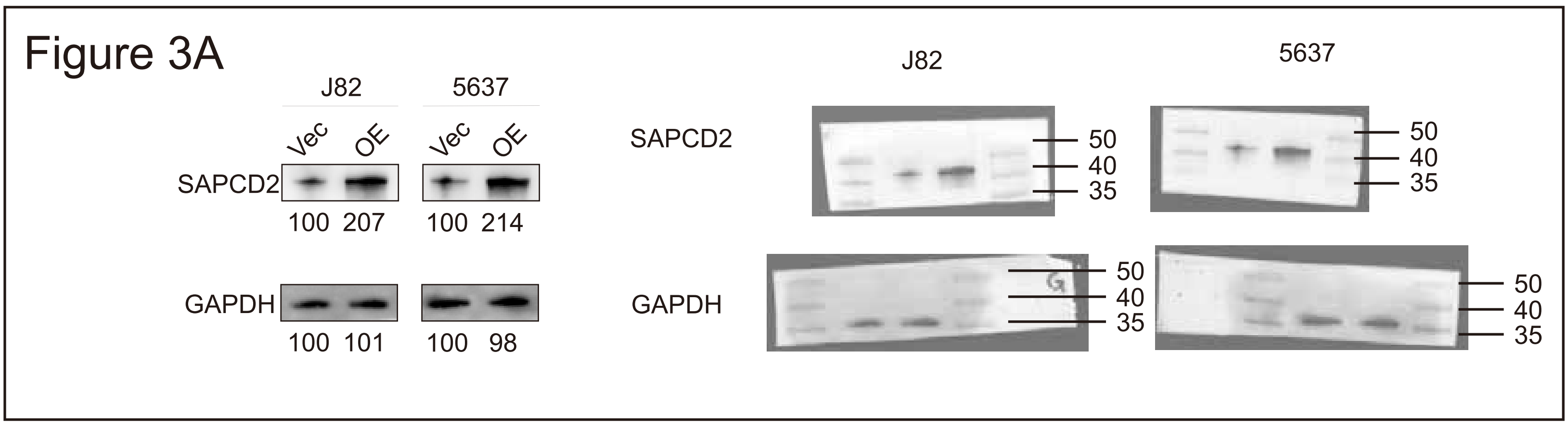
Figure 3A
J82
5637
Vec
OE
SAPCD2
100 207
100 214
GAPDH
100 101
100 98
50
40
35

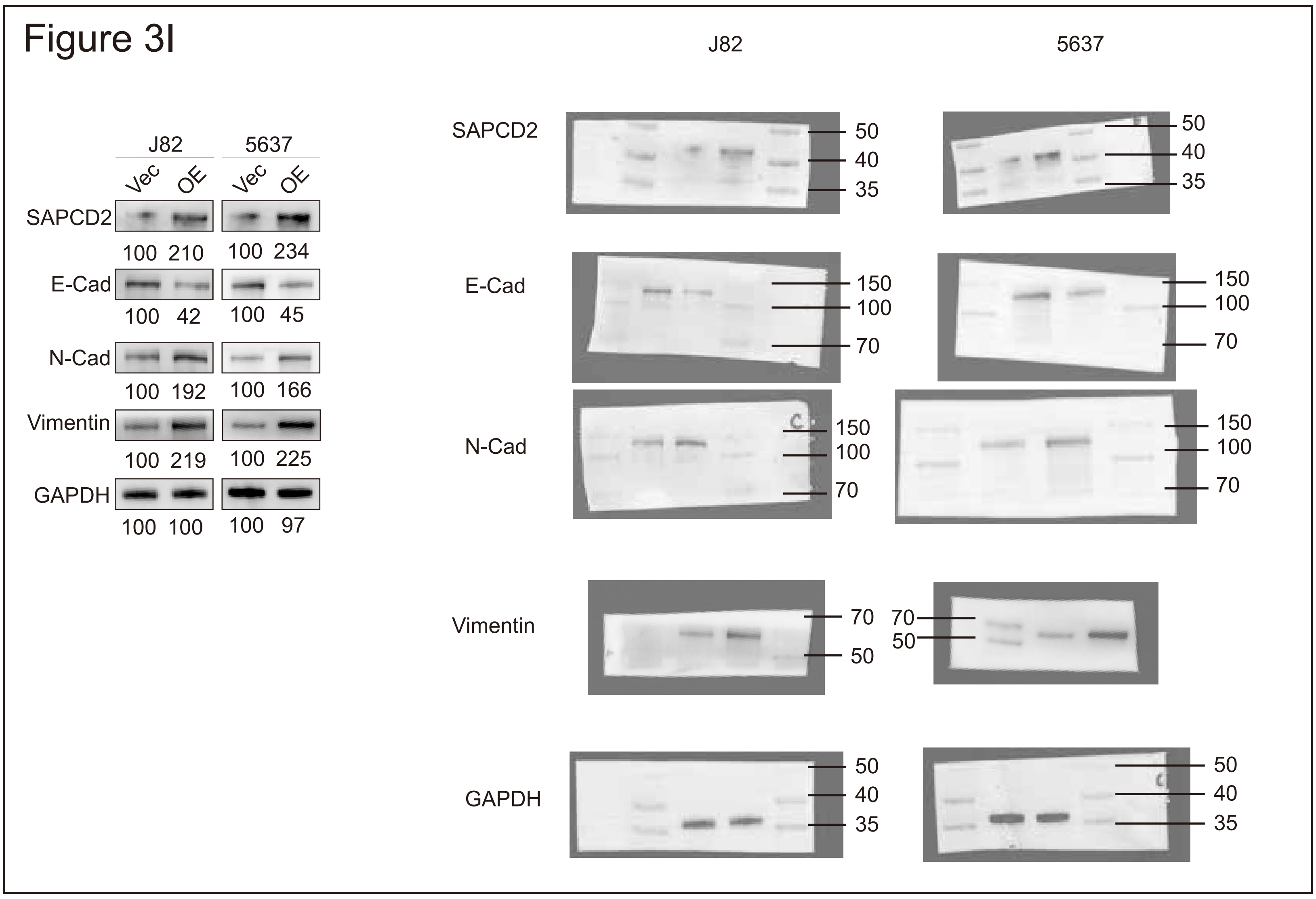
Figure 3I
J82
5637
Vec
OE
SAPCD2
100 210
100 234
E-Cad
100 42
100 45
N-Cad
100 192
100 166
Vimentin
100 219
100 225
GAPDH
100 100
100 97
150
100
70
50
40
35

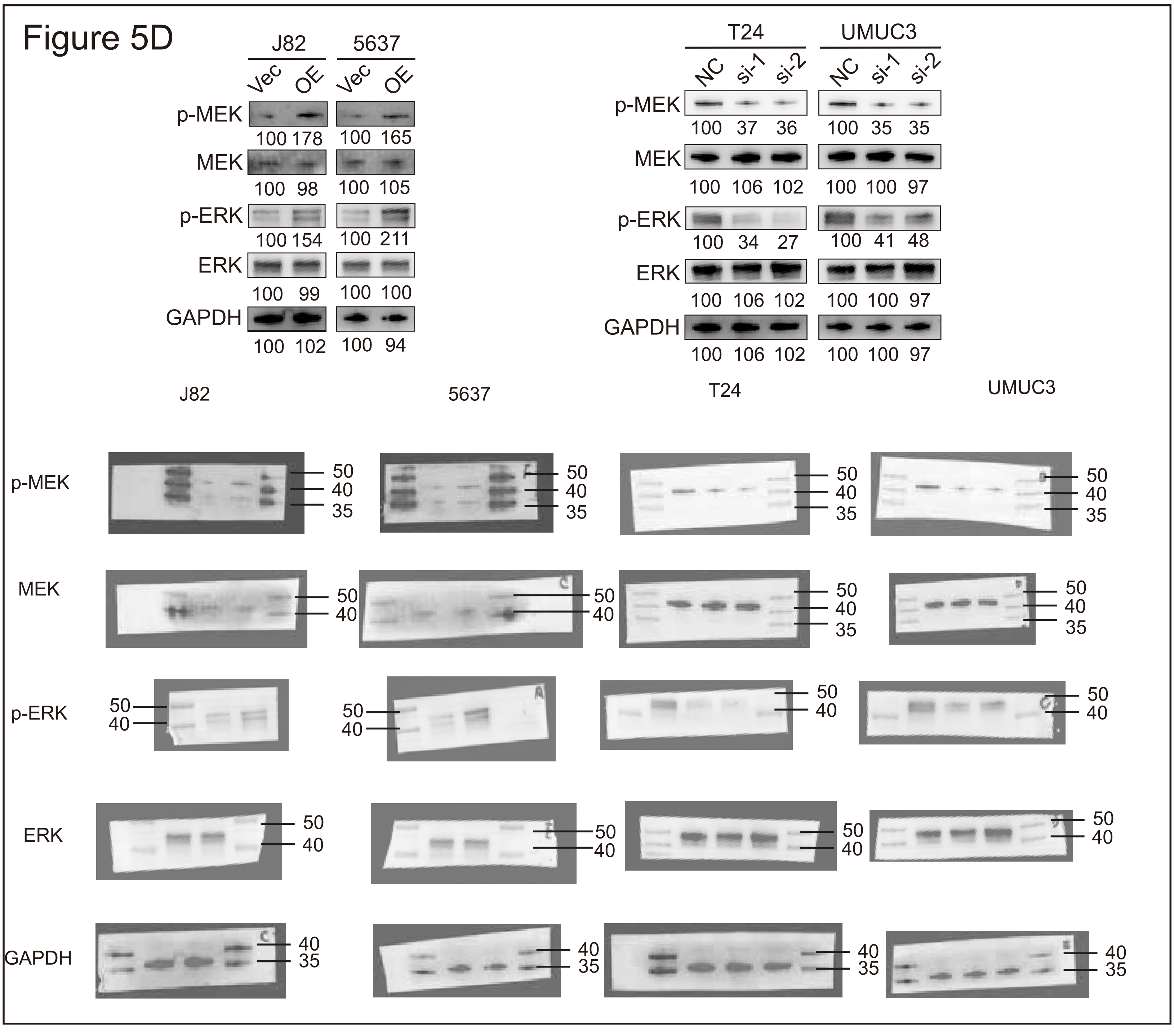
Figure 5D
J82
5637
Vec
OE
p-MEK
100 178
100 165
MEK
100 98
100 105
p-ERK
100 154
100 211
ERK
100 99
100 100
GAPDH
100 102
100 94
T24
UMUC3
NC
si-1
si-2
p-MEK
100 37 36
100 35 35
MEK
100 106 102
100 100 97
p-ERK
100 34 27
100 41 48
ERK
100 106 102
100 100 97
GAPDH
100 106 102
100 100 97
50
40
35

Figure 6E

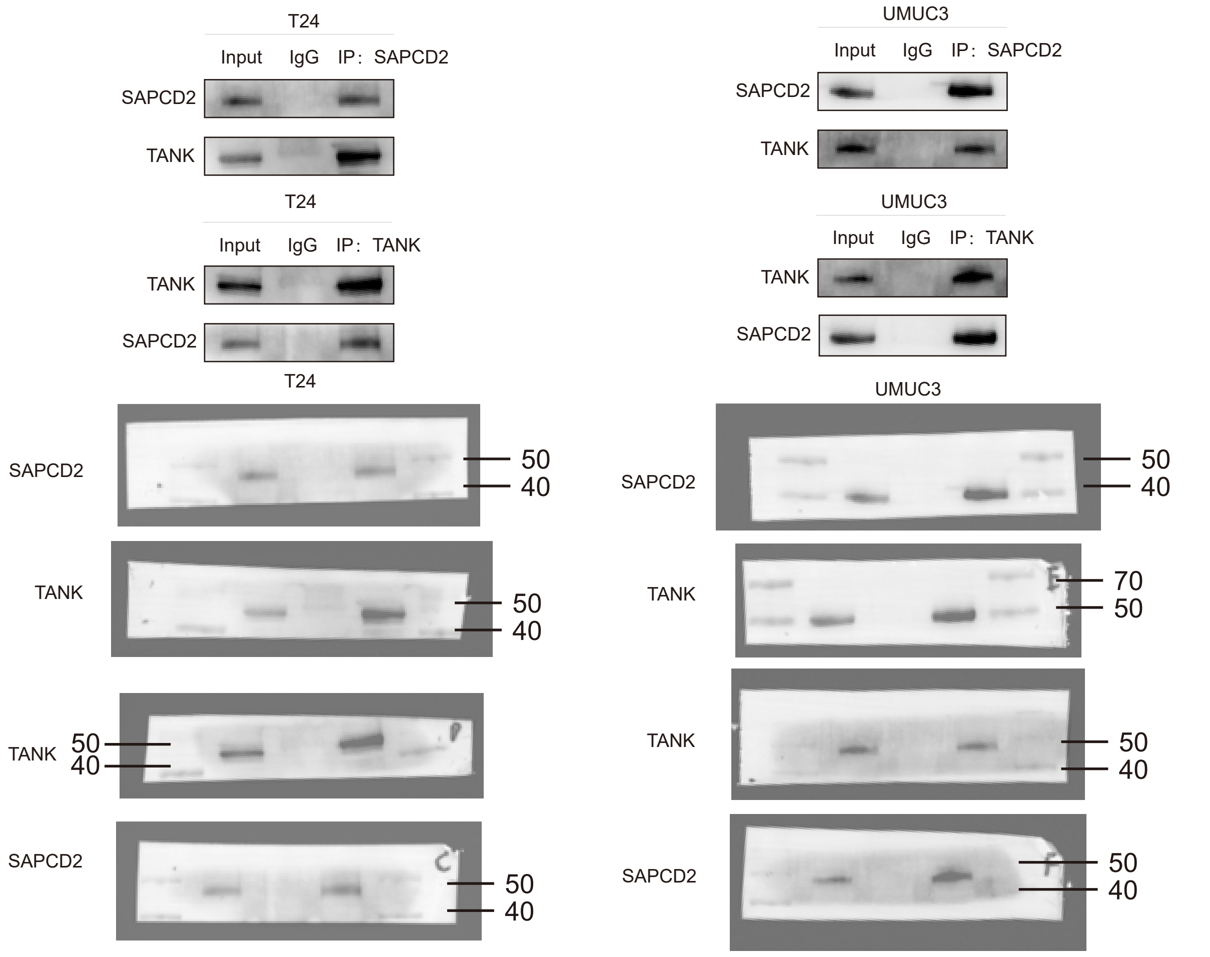

Figure 6F

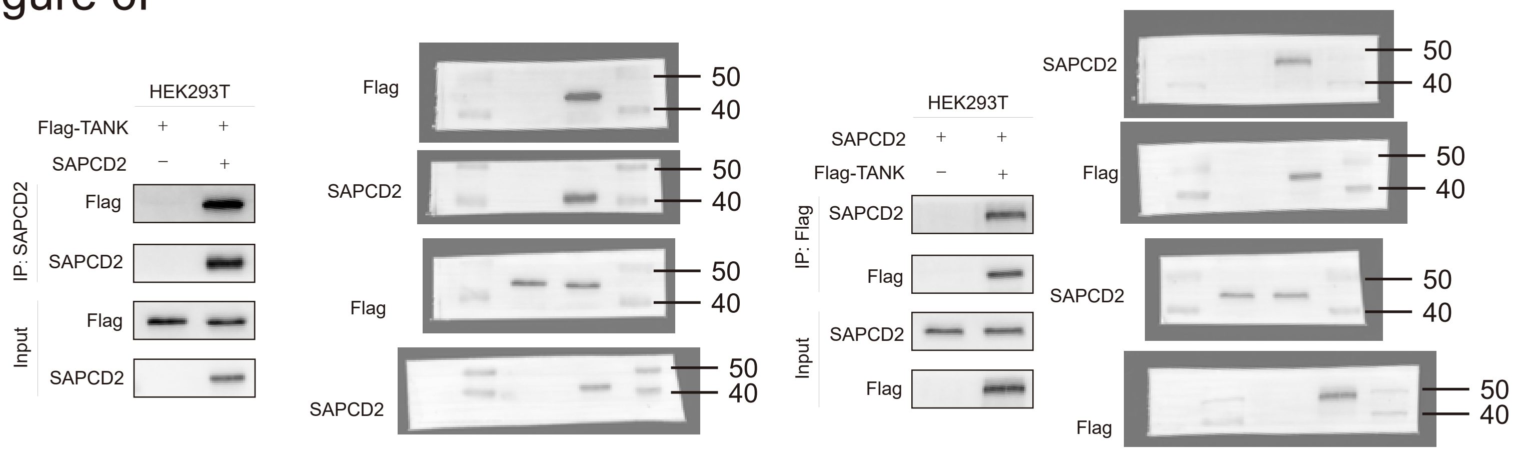

Figure 6I

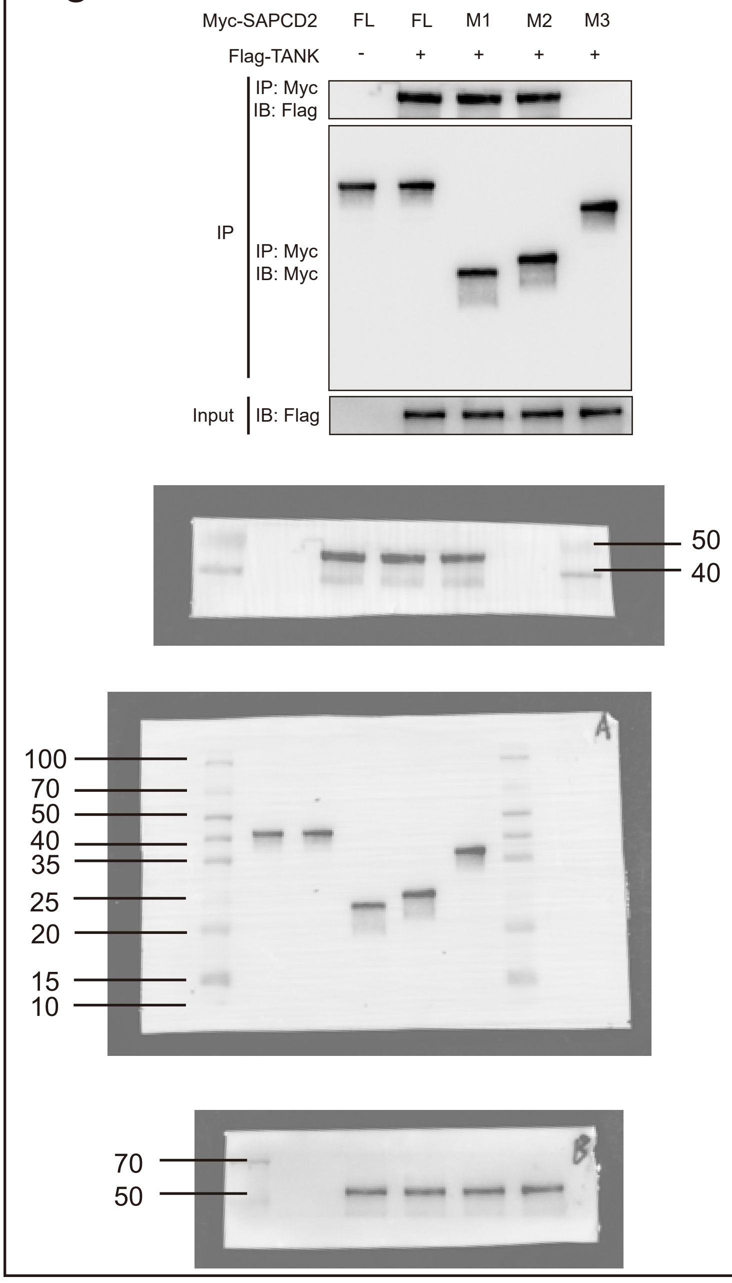

Figure 6J

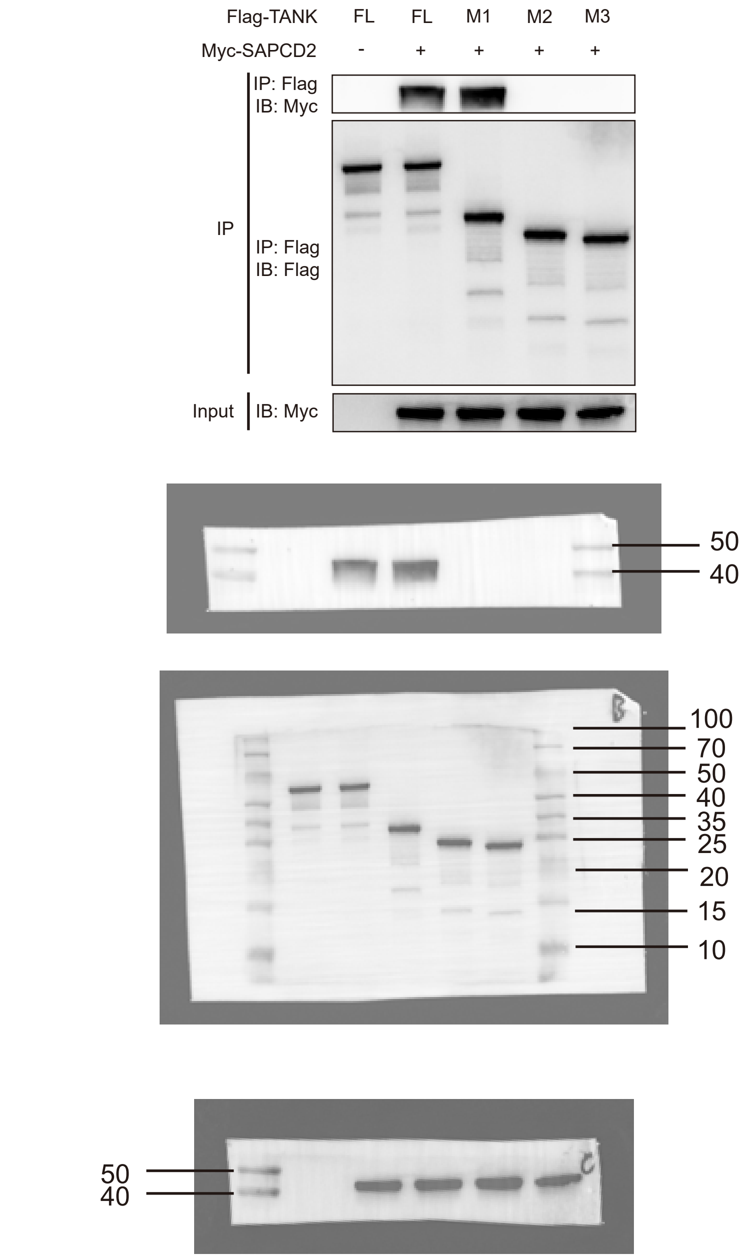

Figure 7A

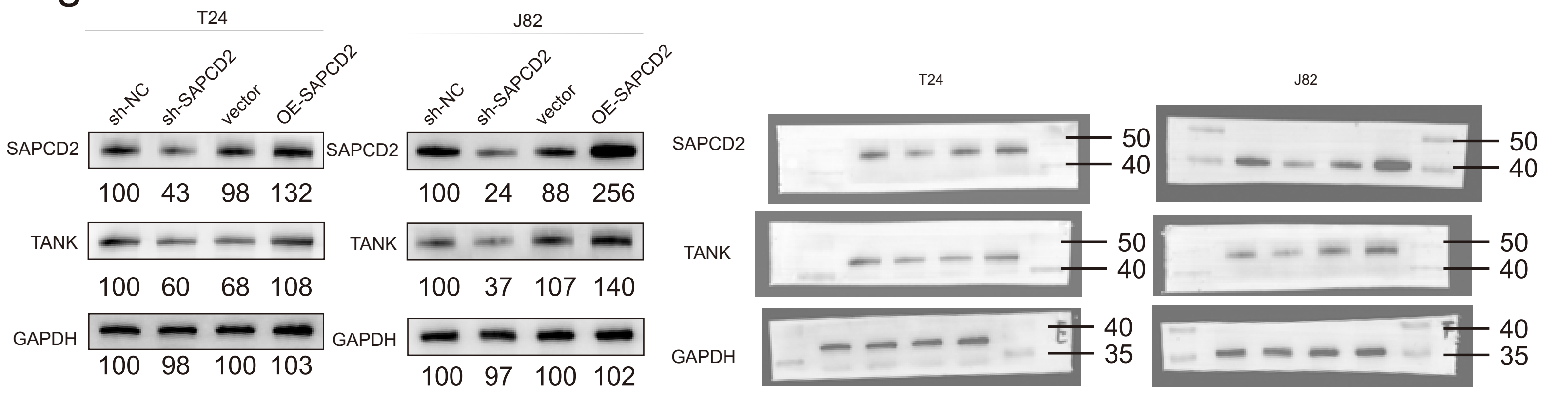

Figure 7B

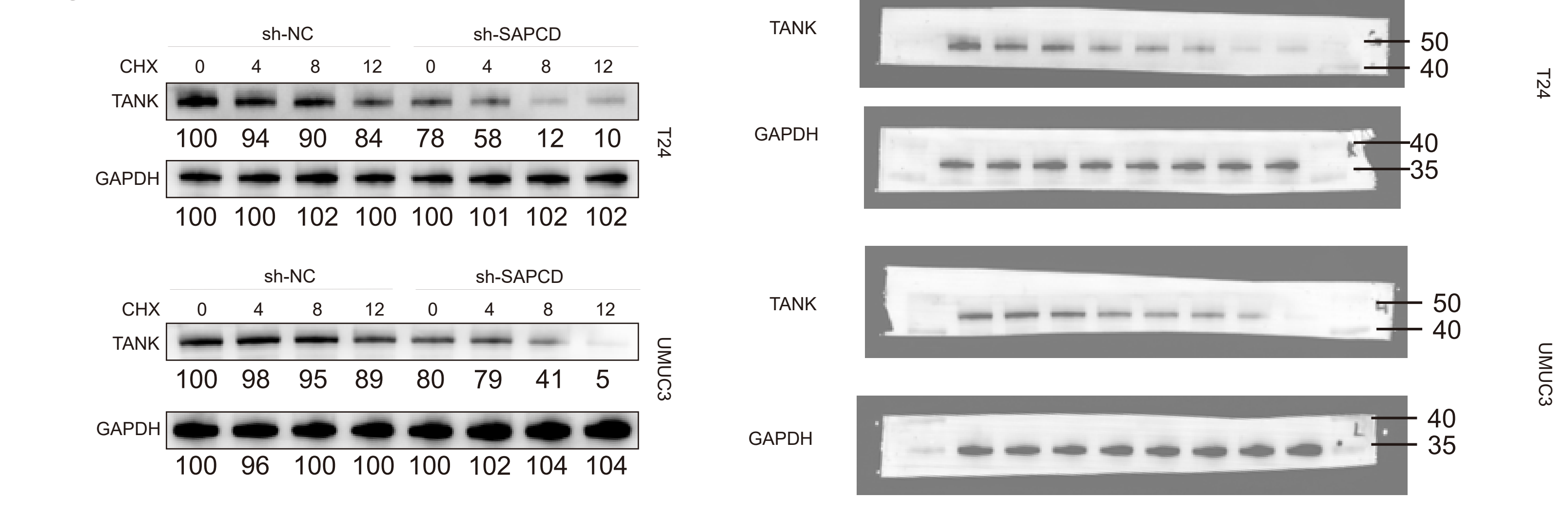

Figure 7C

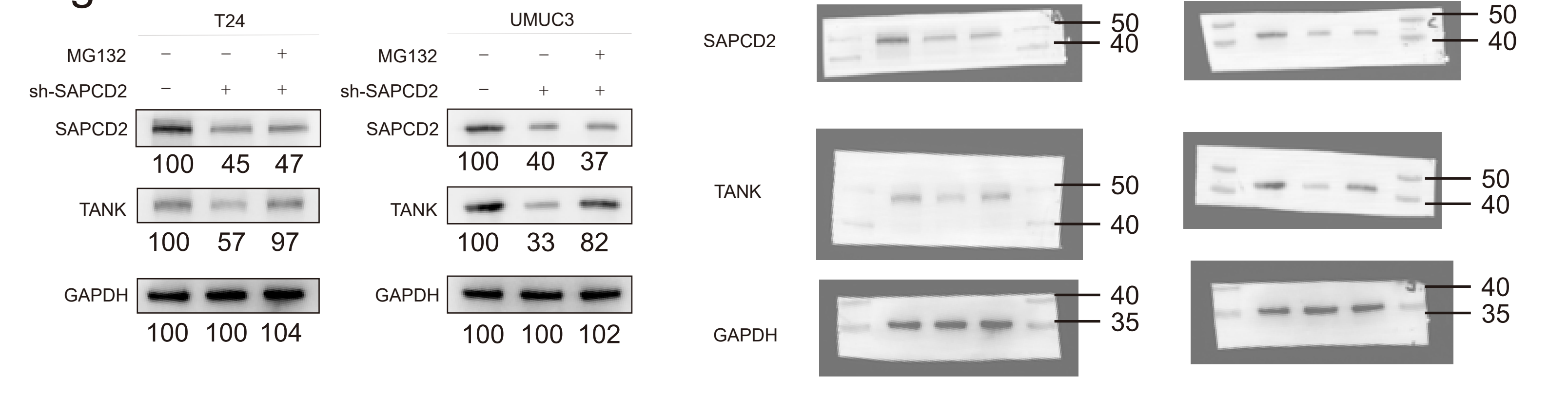

Figure 7D

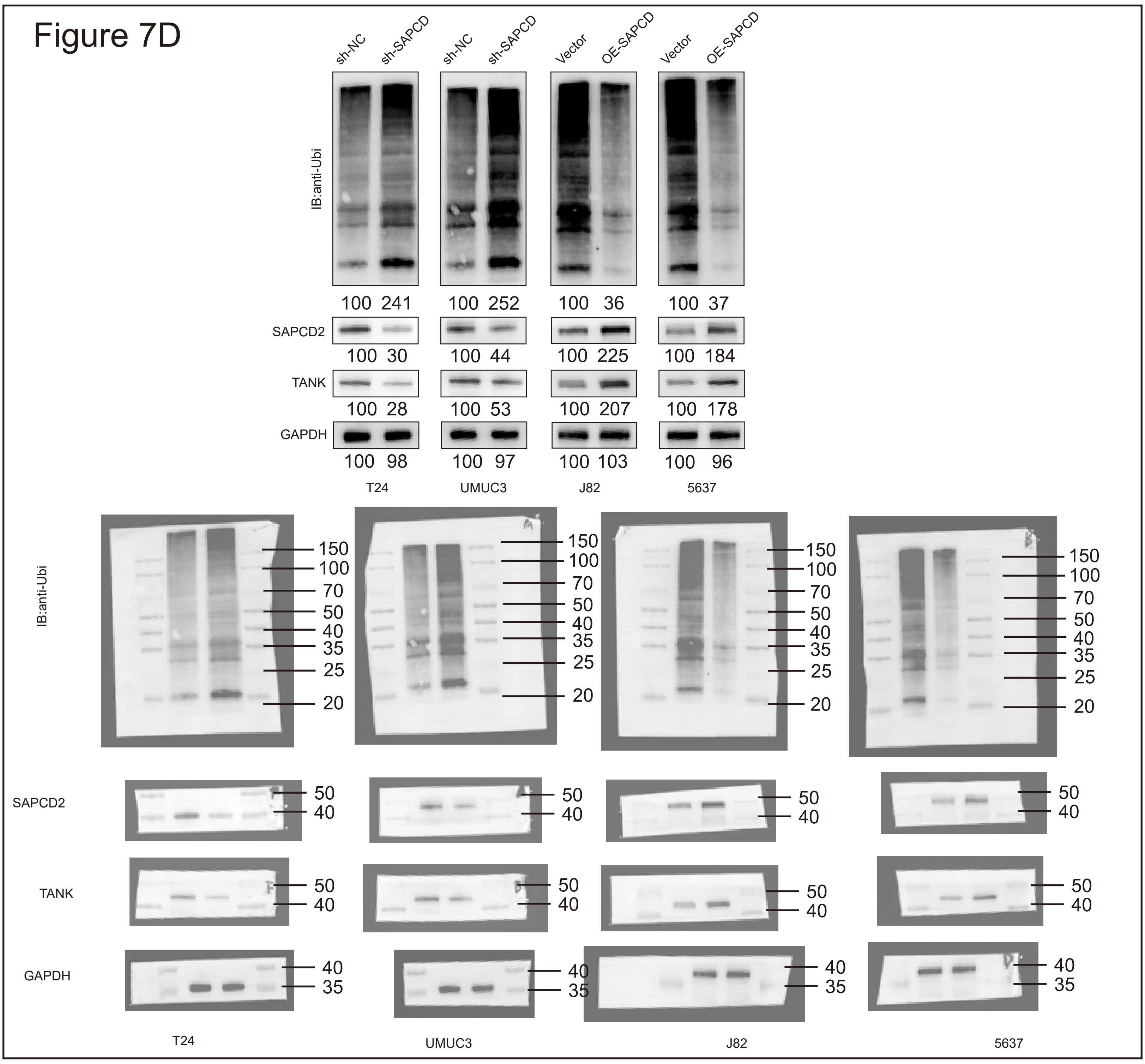

Figure 7E

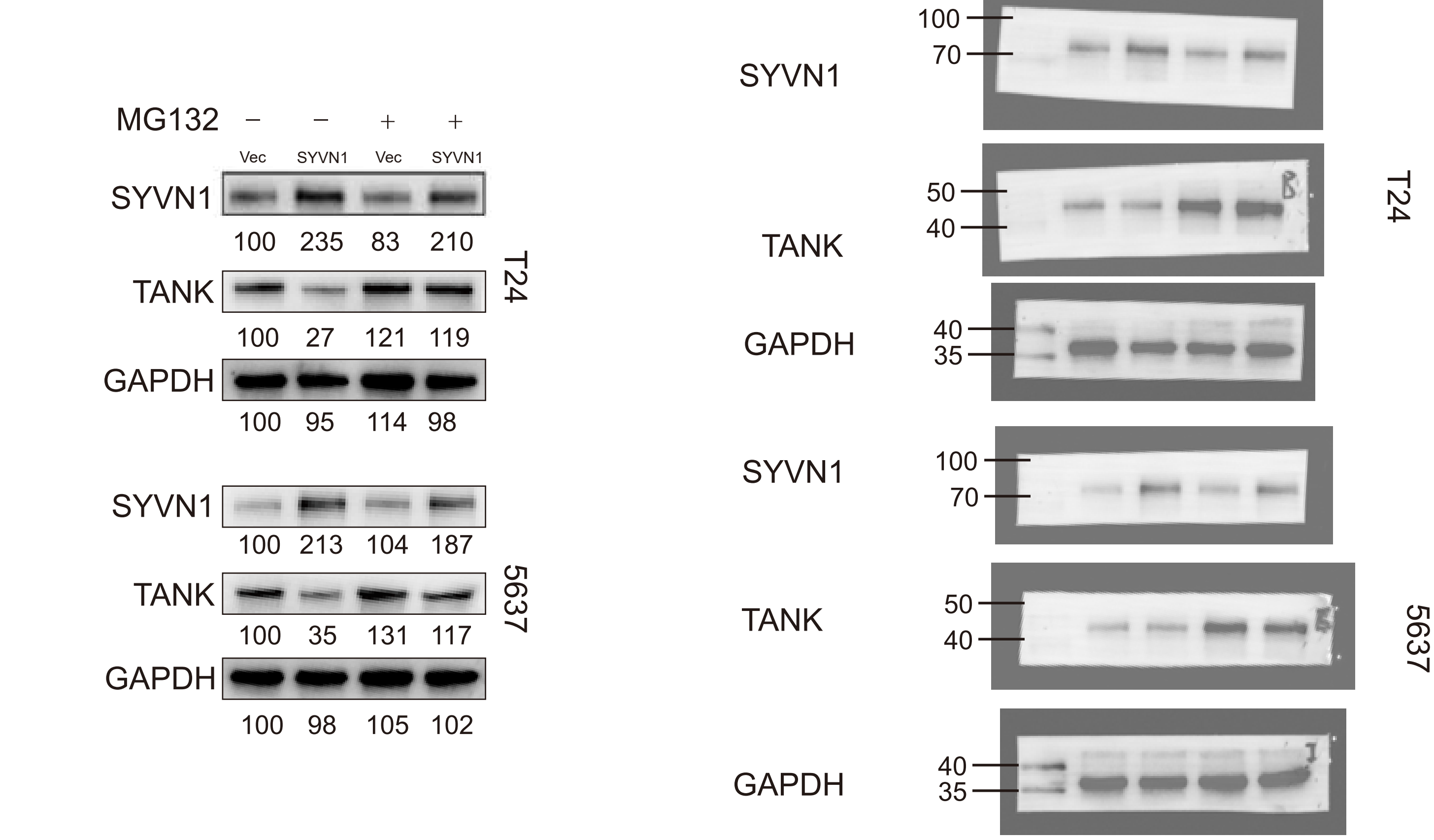

Figure 7F

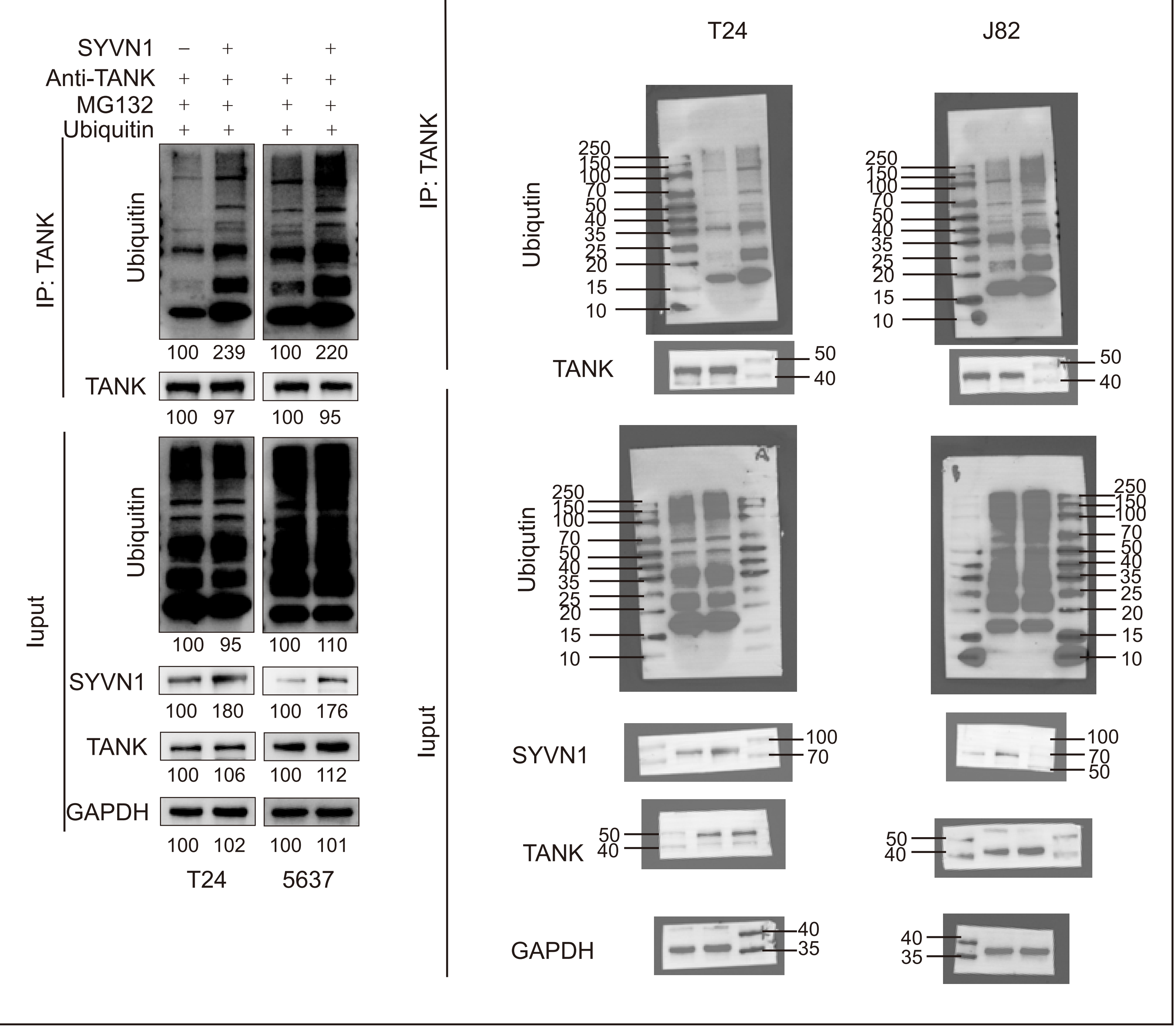

Figure 7G

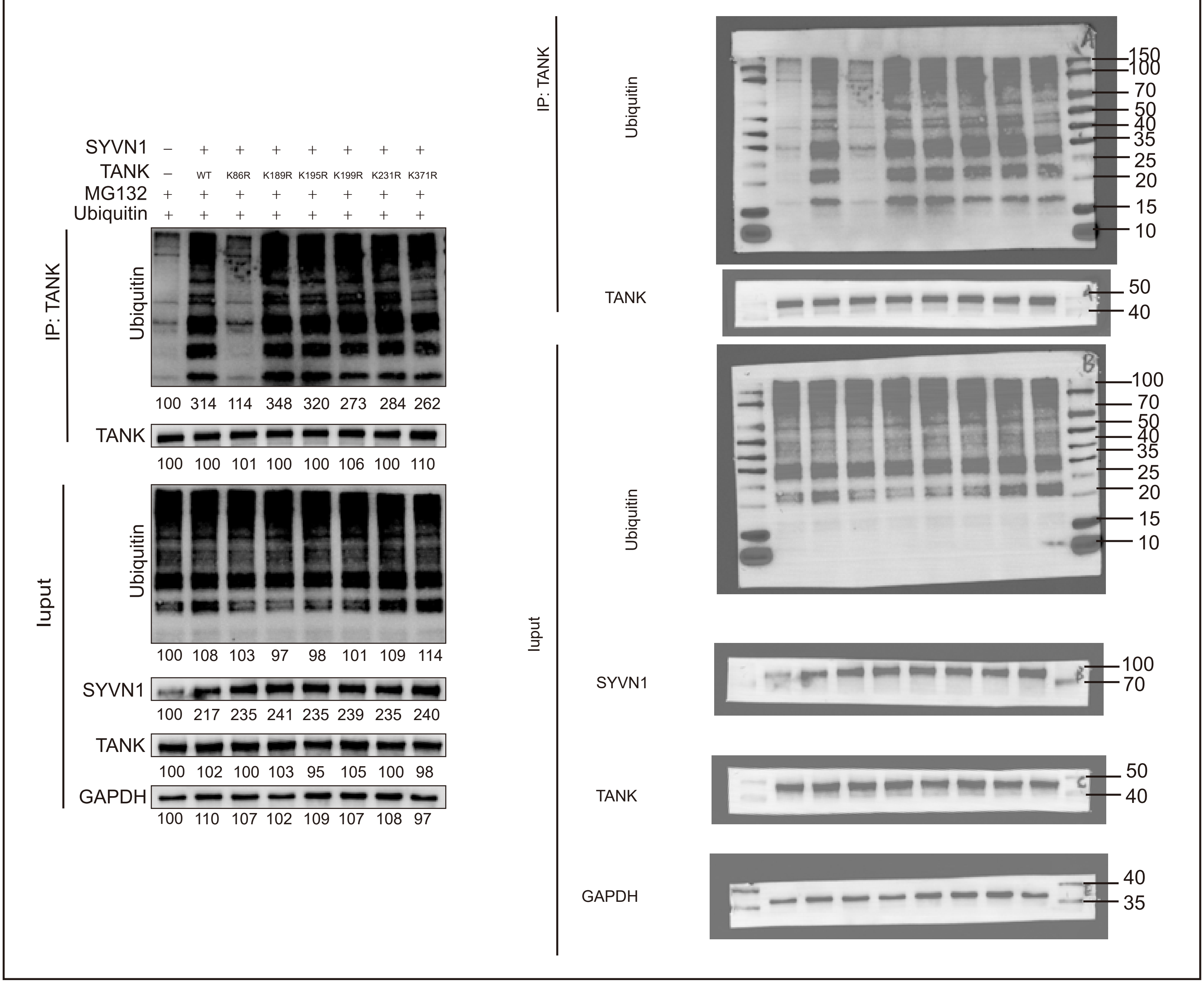

Figure 7H

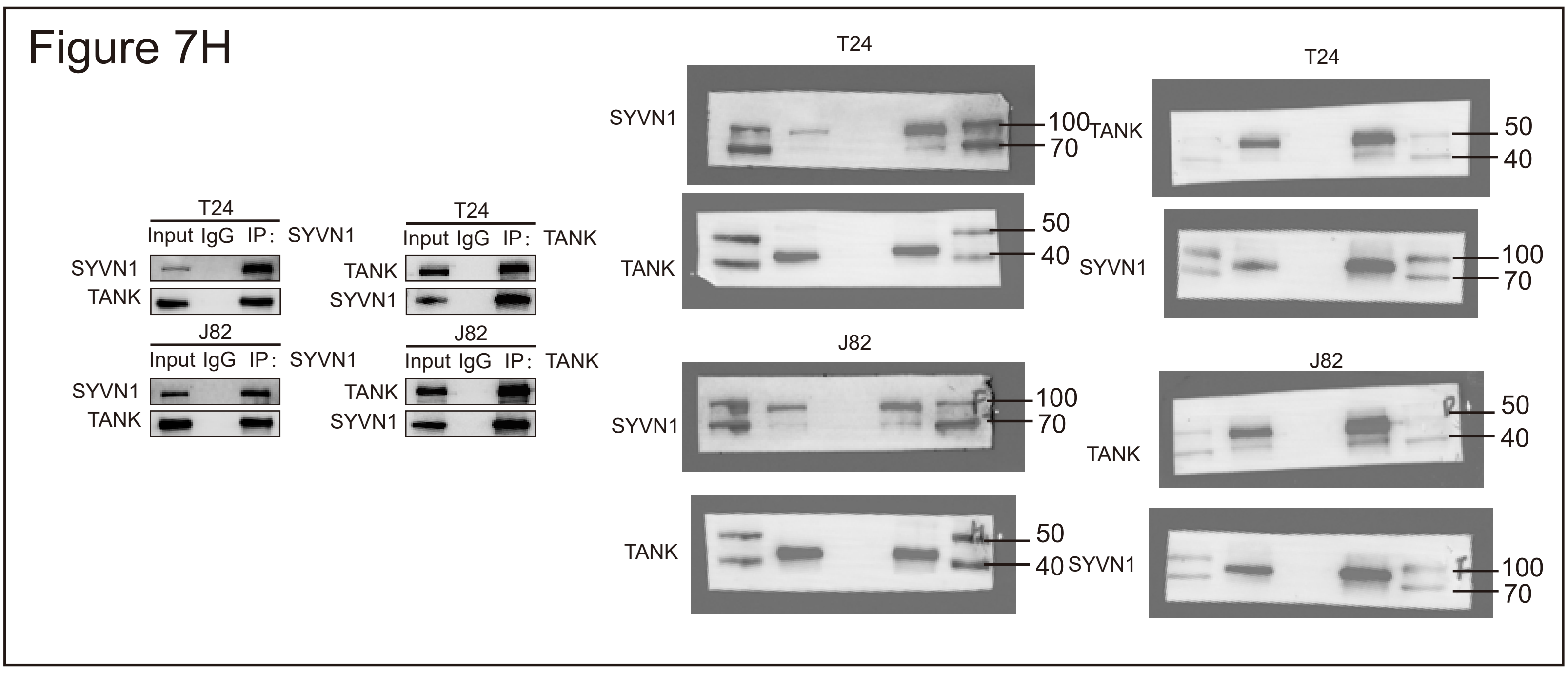

Figure 7I

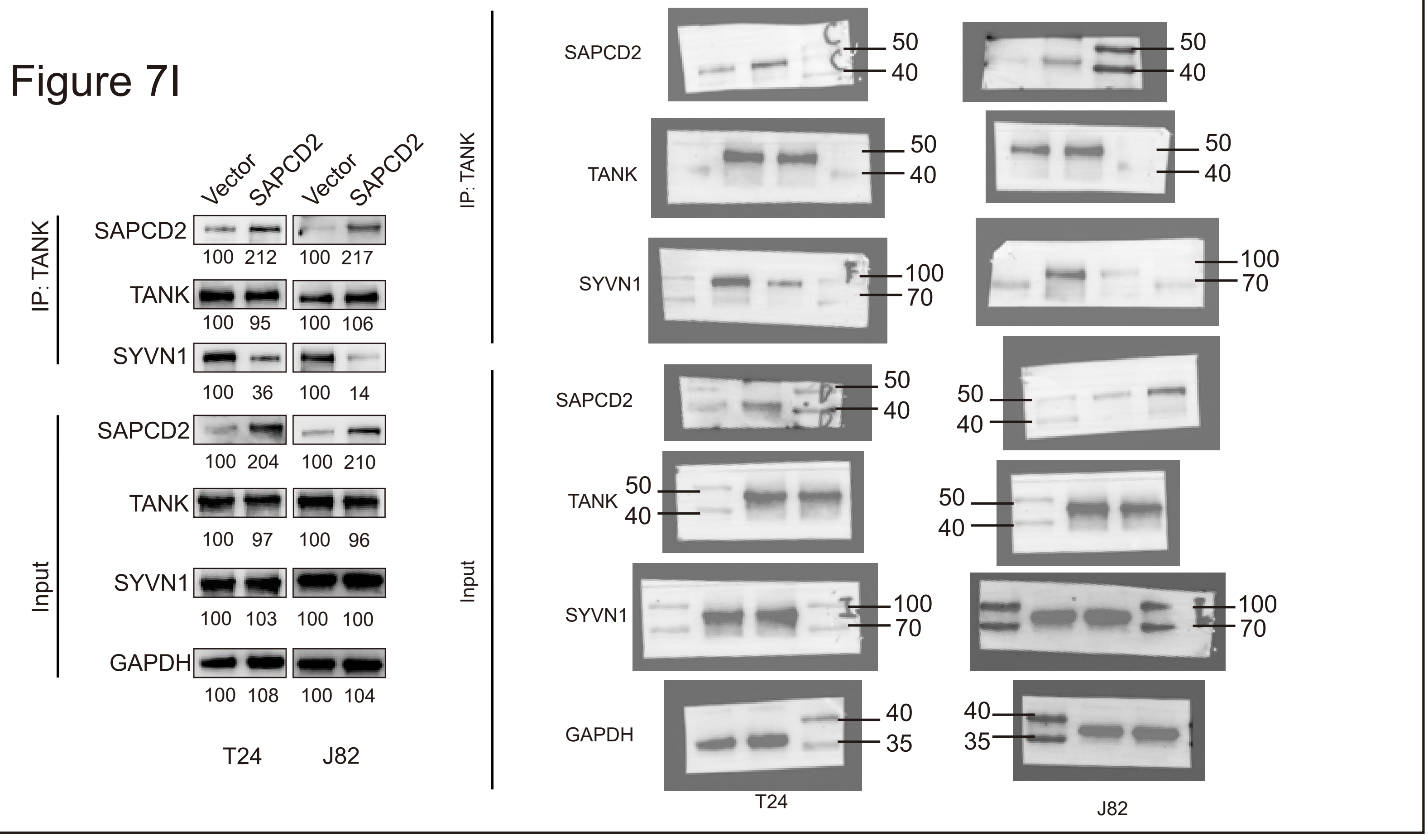

Figure 8F

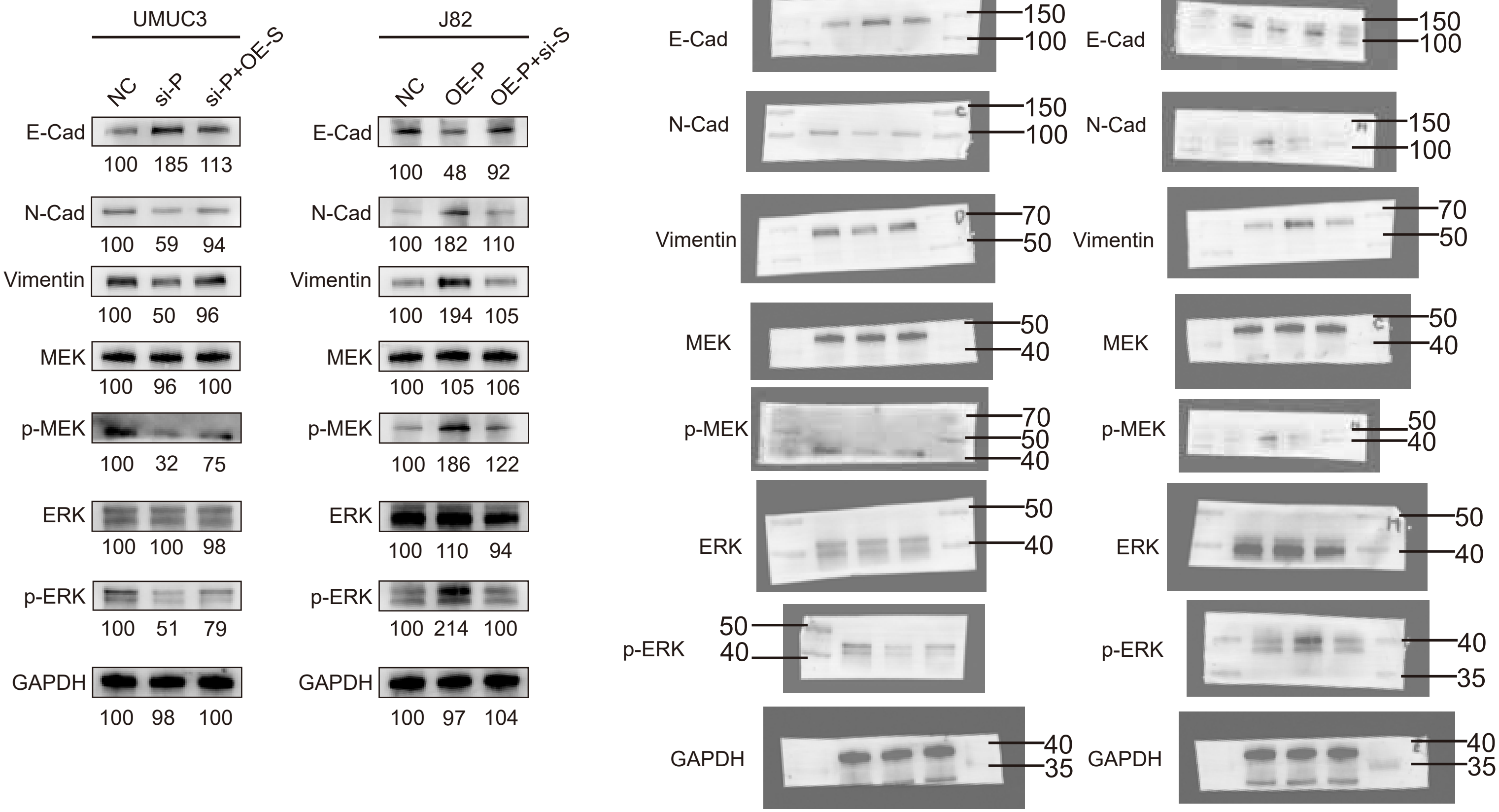
UMUC3
NC
si-P
si-P+OE-S
E-Cad
100 185 113
N-Cad
100 59 94
Vimentin
100 50 96
MEK
100 96 100
p-MEK
100 32 75
ERK
100 100 98
p-ERK
100 51 79
GAPDH
100 98 100
J82
NC
OE-P
OE-P+si-S
E-Cad
100 48 92
N-Cad
100 182 110
Vimentin
100 194 105
MEK
100 105 106
p-MEK
100 186 122
ERK
100 110 94
p-ERK
100 214 100
GAPDH
100 97 104
UMUC3
E-Cad 150 100
N-Cad 150 100
Vimentin 70 50
MEK 50 40
p-MEK 70 50 40
ERK 50 40
p-ERK 50 40
GAPDH 40 35
J82
E-Cad 150 100
N-Cad 150 100
Vimentin 70 50
MEK 50 40
p-MEK 50 40
ERK 50 40
p-ERK 40 35
GAPDH 40 35

Figure 9D

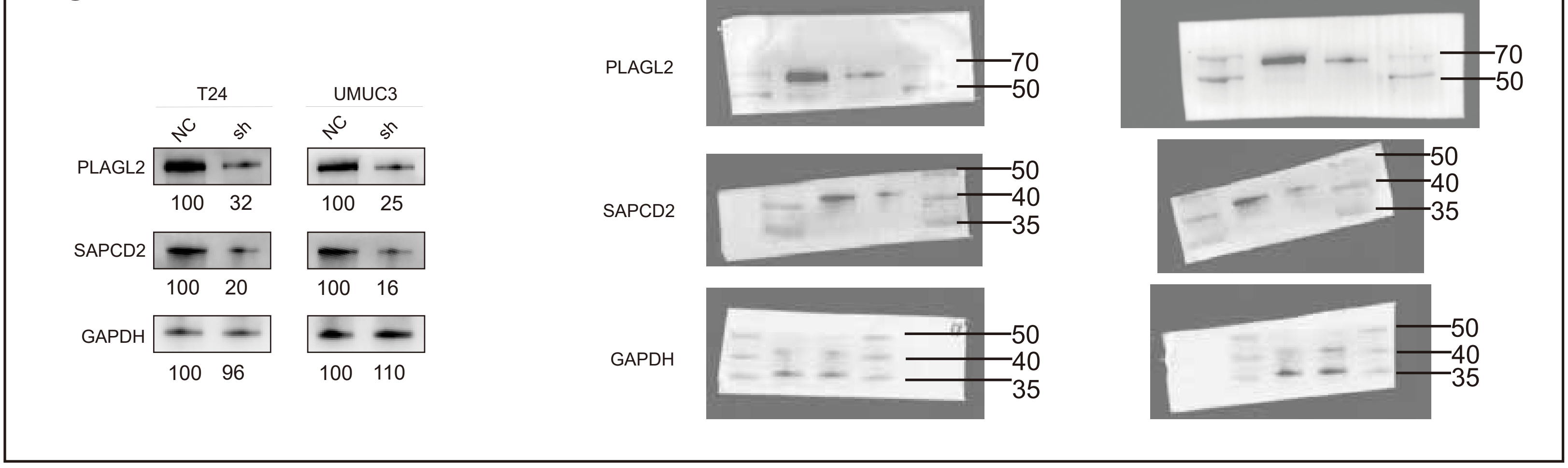

Figure 9E

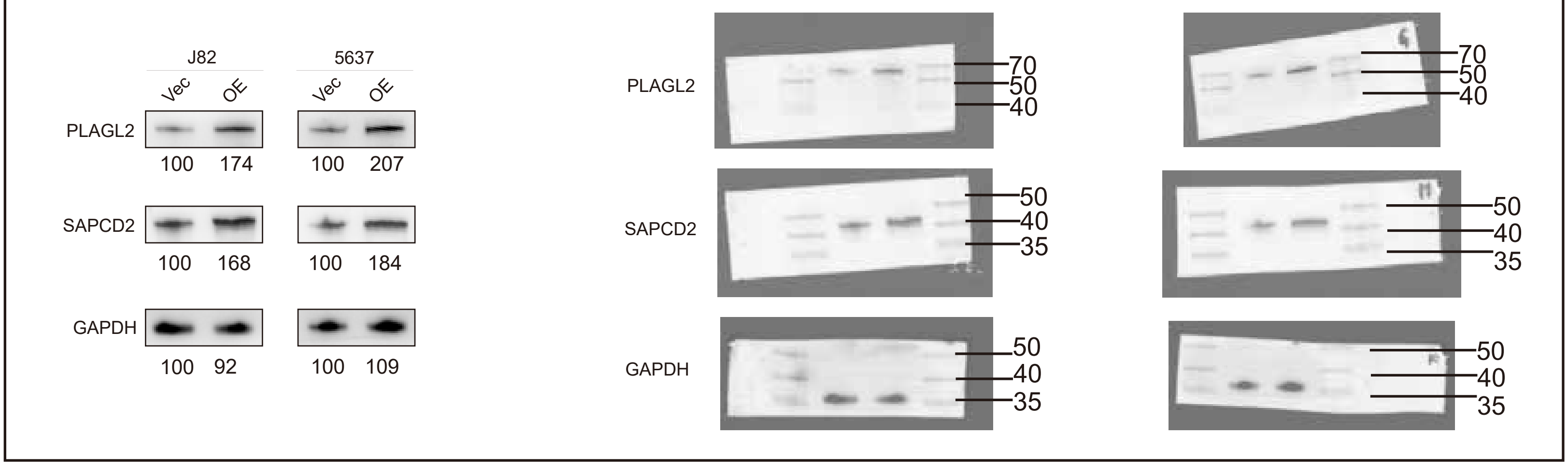

Figure 9F

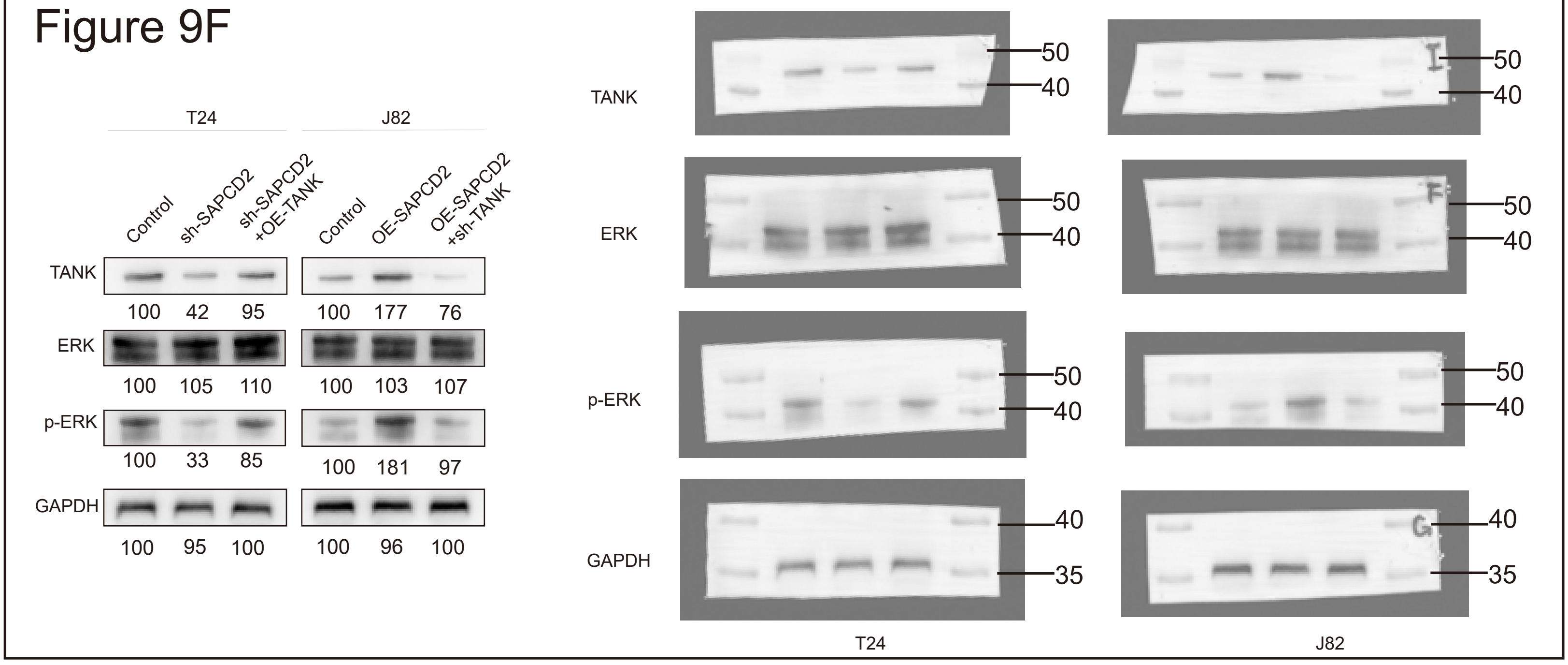

Figure 9G

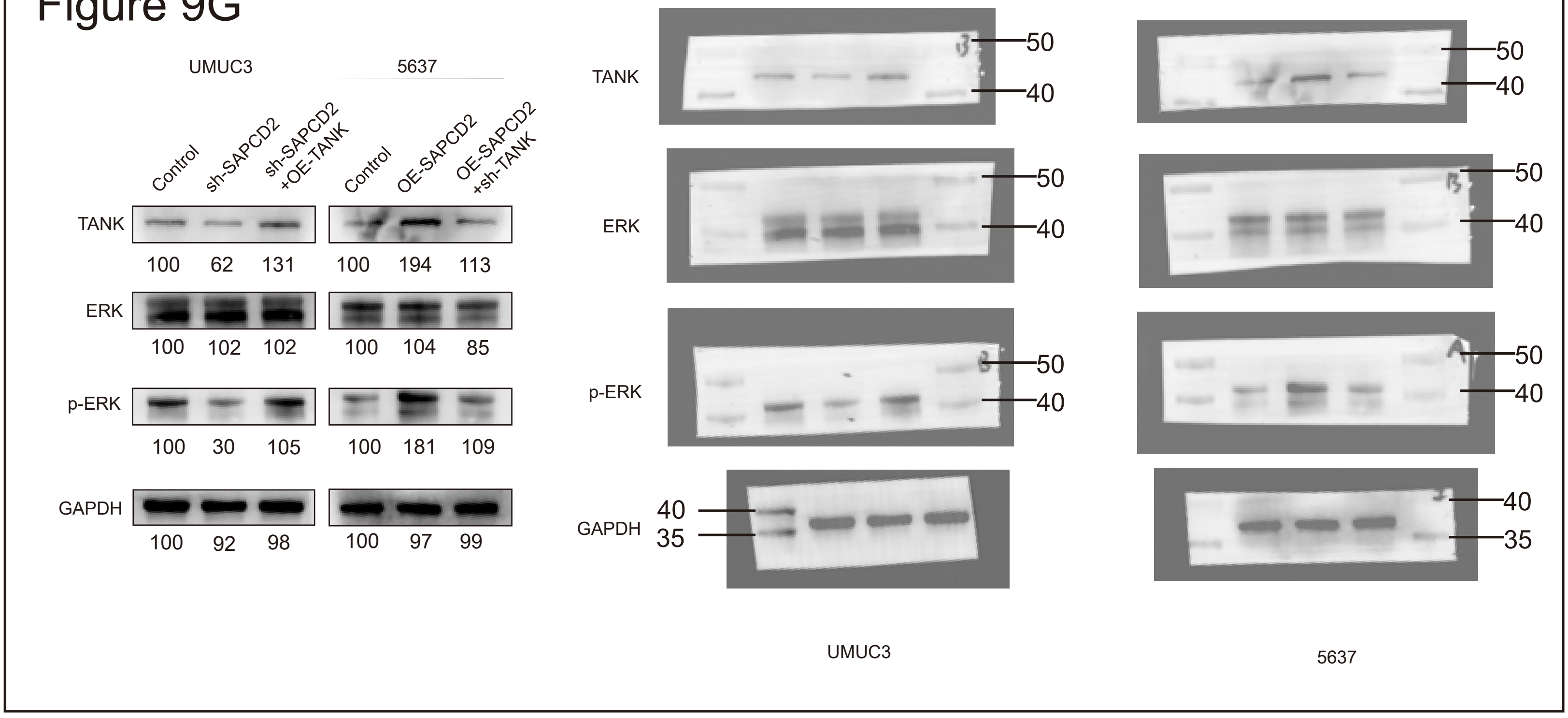

Figure 9K

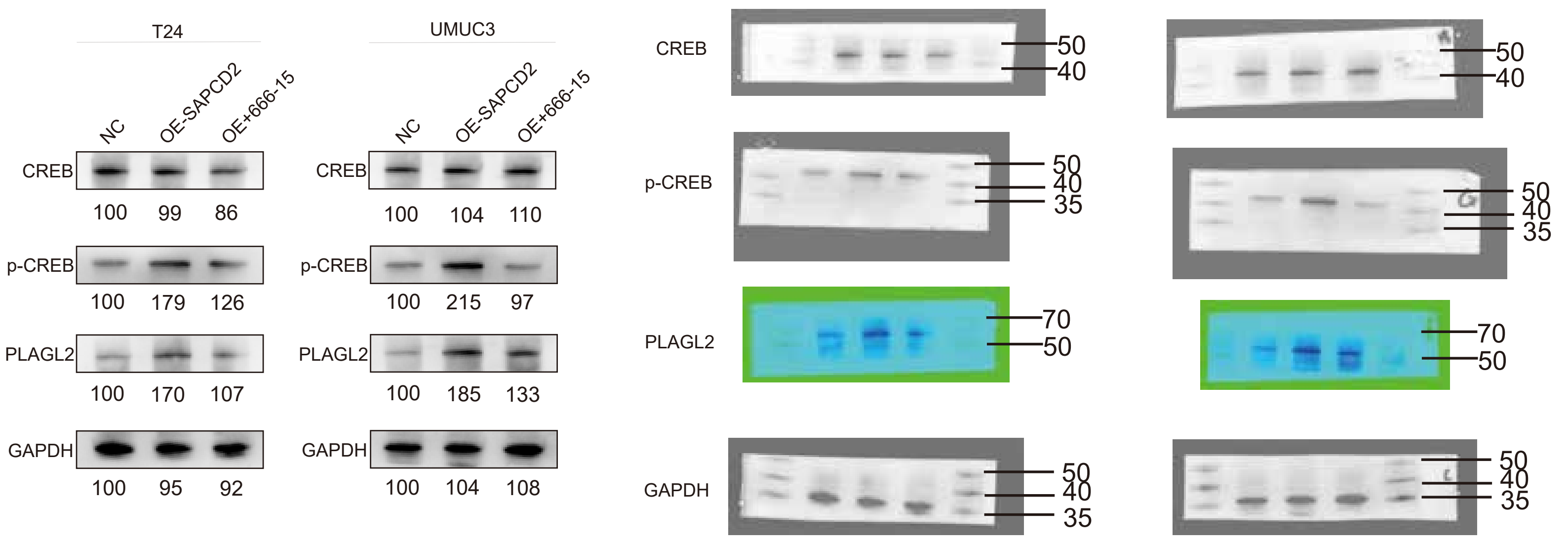

Figure 9M

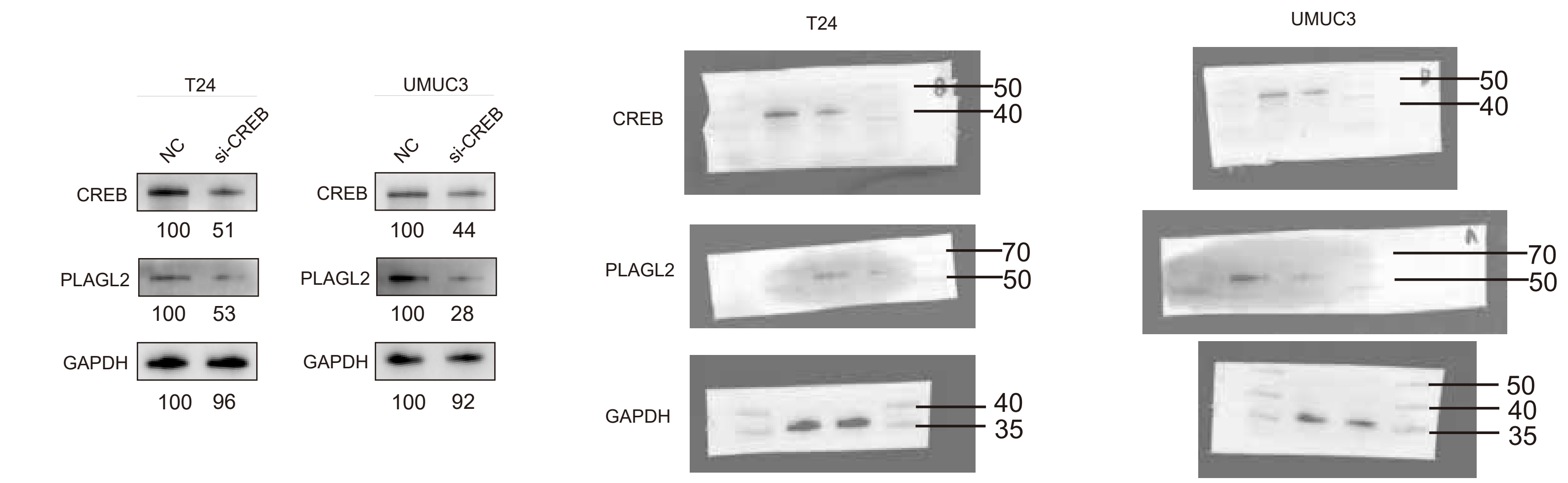

Figure S4C

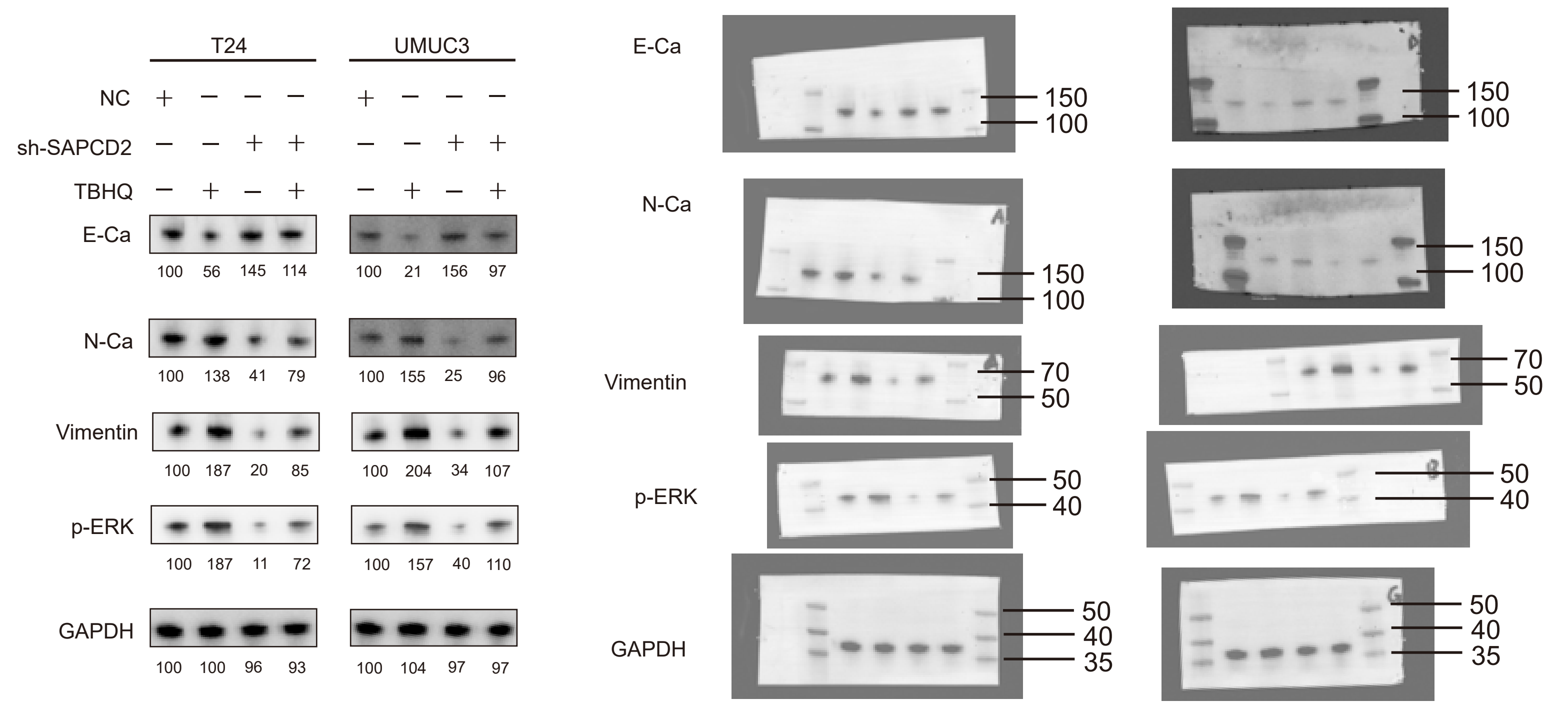

Figure S4D

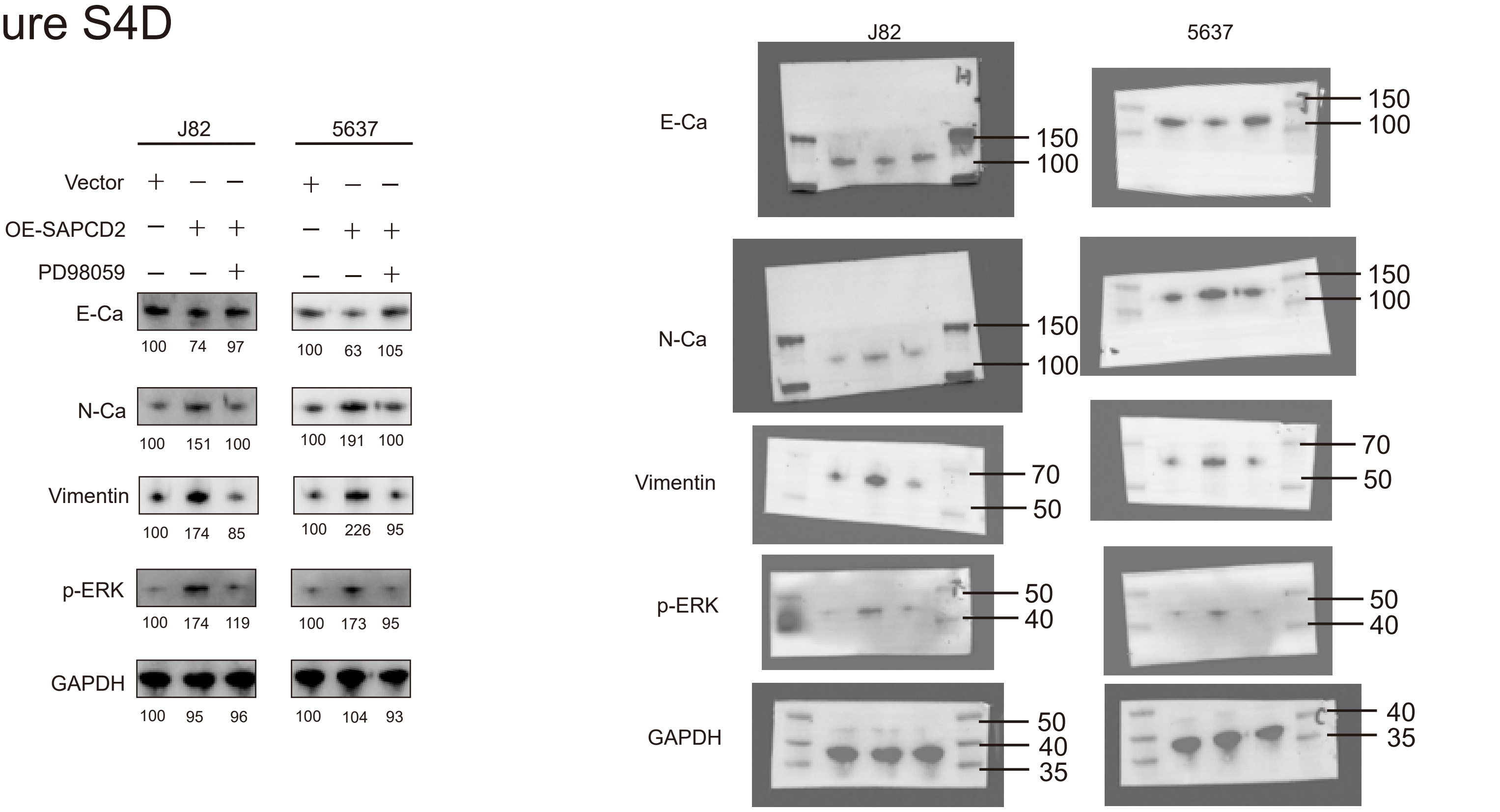

Figure S5A

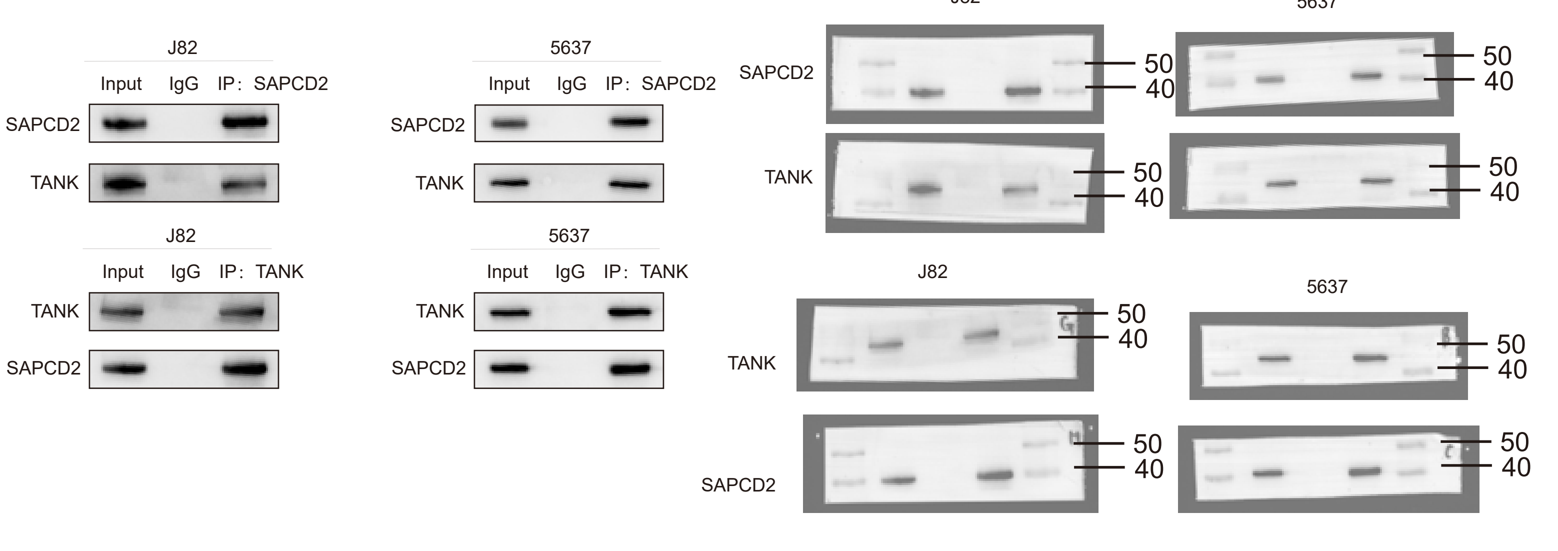

Figure S6F

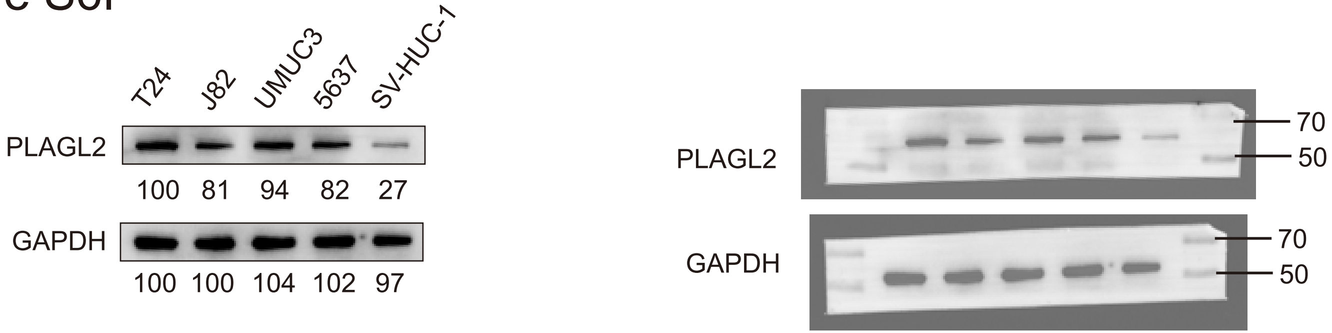

Figure S6G

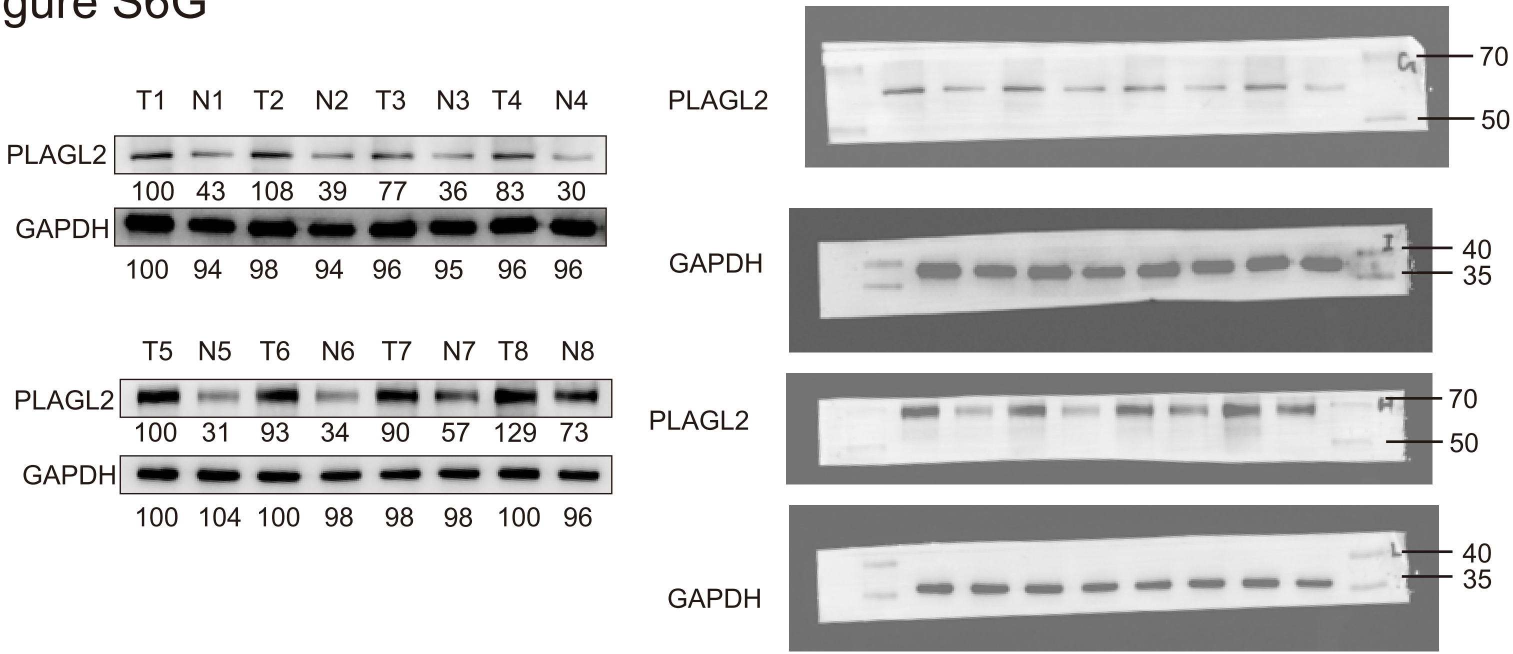

Supplement: Supplementary file 1 [file cancers-18-00535-s001.zip › File S1.pdf]
