# Supplementary material for: SAPCD2 Drives Bladder Cancer Progression by Stabilizing TANK and Engaging a CREB–PLAGL2 Feedback Loop to Sustain MAPK Signaling
Source: Cancers (Basel). 2026 Feb 6;18(3):535. doi: 10.3390/cancers18030535 (PMC12896413; doi:10.3390/cancers18030535)
Supplement: Supplementary file 1 [file cancers-18-00535-s001.zip › Supplementary file.pdf]

Supplementary figures

1. Figure S1

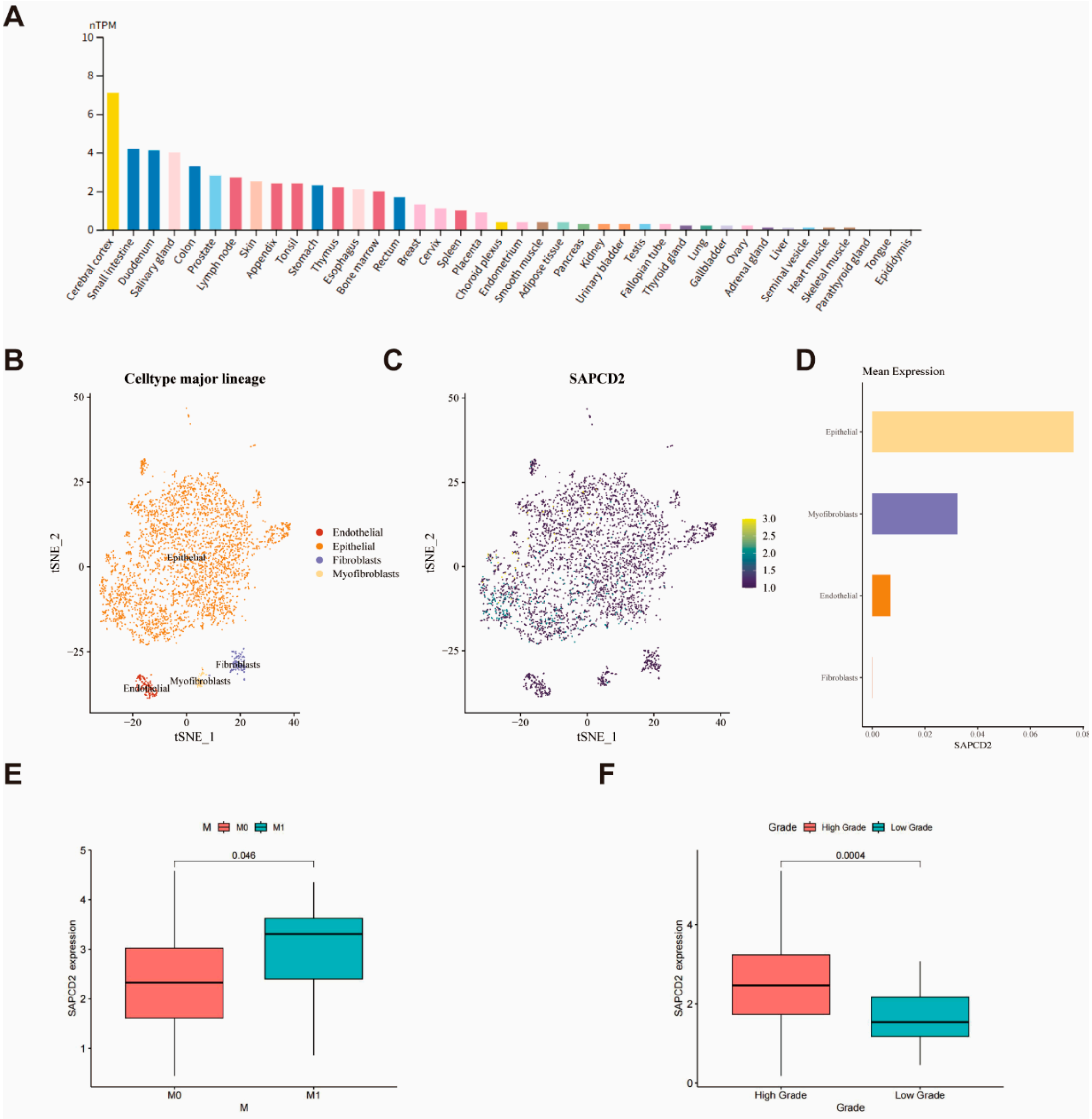

Figure S1. SAPCD2 Expression in Normal Human Tissues and Bladder Cancer.

(A) SAPCD2 expression across normal human tissues. (B) Single-cell RNA-seq t-SNE plot from the GSE130001 dataset, illustrating cell clusters with distinct lineages (endothelial, epithelial, fibroblasts, myofibroblasts) in bladder cancer. (C) t-SNE plot of SAPCD2 expression in the single-cell RNA-seq dataset, with color intensity indicating gene expression levels. (D) The mean SAPCD2 expression levels across different cell types in the bladder cancer single-cell dataset. (E) Correlation of SAPCD2 expression with metastatic stage in bladder cancer patients from TCGA. (F) Correlation of SAPCD2 expression with tumor grade in bladder

cancer patients from TCGA.

2. Figure S2

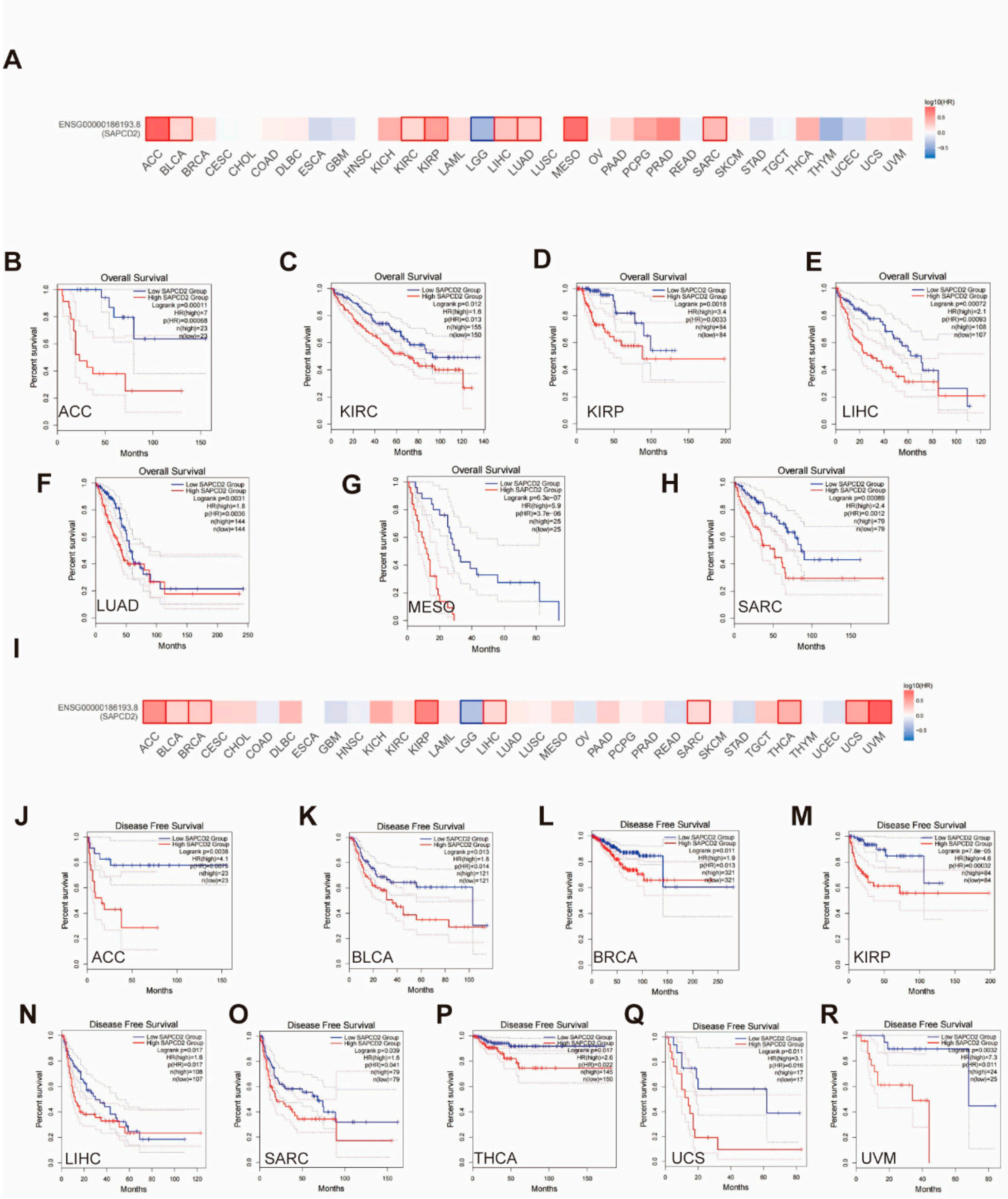

Figure S2. SAPCD2 Expression and Its Impact on Survival in Multiple Cancer Types. (A) Pan-cancer survival analysis showing the correlation between SAPCD2 expression and overall survival (OS) across various tumor types from TCGA. Red indicates poorer OS associated with higher SAPCD2 expression. (B–H) Kaplan-Meier curves showing OS in high and low SAPCD2 expression groups for various cancer types, including ACC (S2B), KIRC (S2C), KIRP (S2D), LIHC (S2E), LUAD (S2F), MESO (S2G), and SARC (S2H), where higher SAPCD2 expression

is linked to worse prognosis. (I) Pan-cancer analysis of SAPCD2 expression and disease-free survival (DFS) across multiple cancer types, with red indicating poorer DFS in patients with higher SAPCD2 expression. (J–R) Kaplan-Meier curves showing DFS in high and low SAPCD2 expression groups for various cancer types, including ACC (S2J), BLCA (S2K), BRCA (S2L), KIRP (S2M), LIHC (S2N), SARC (S2O), THCA (S2P), UCS (S2Q), and UVM (S2R), where high SAPCD2 expression correlates with poorer DFS.

3. Figure S3

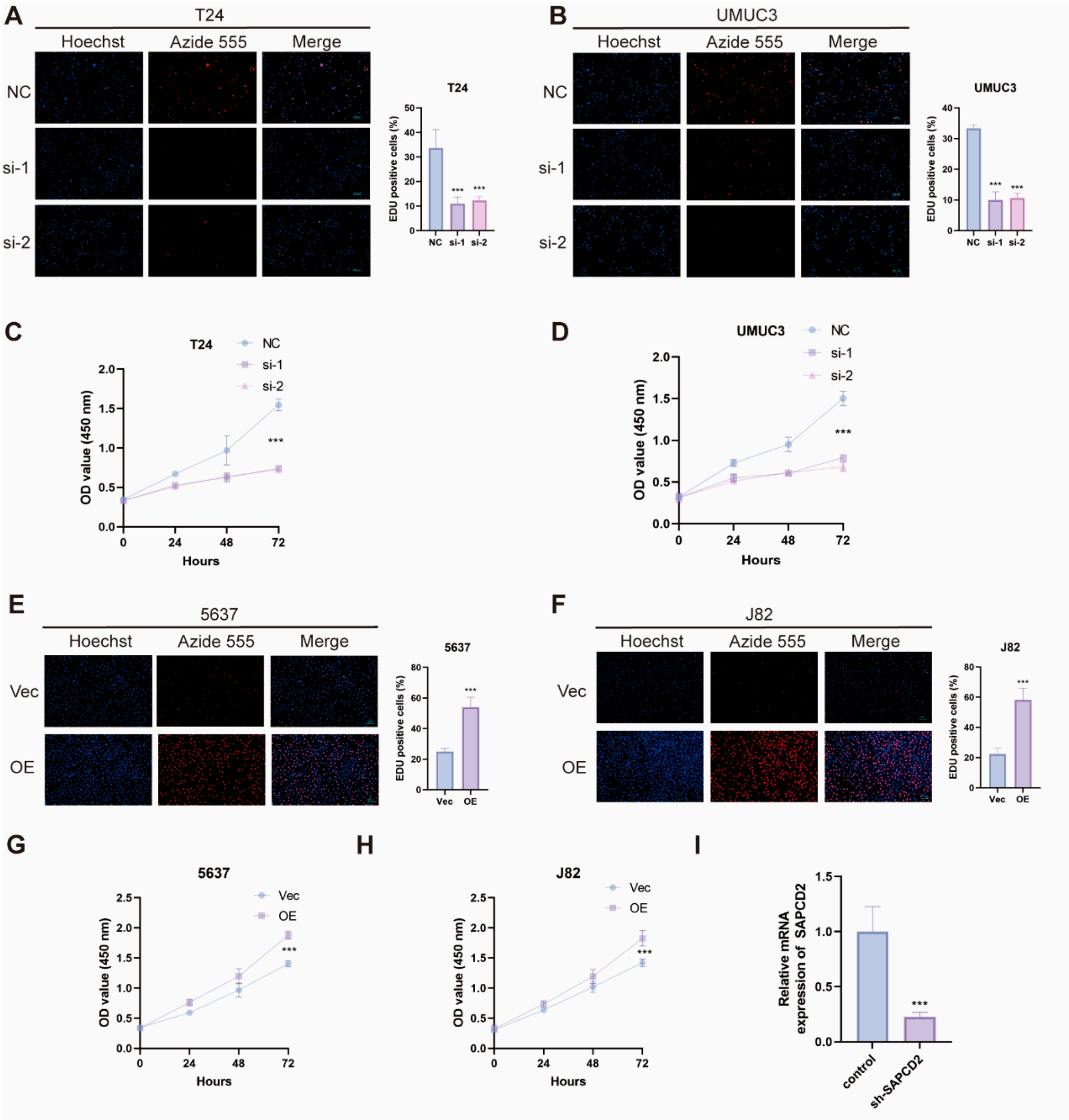

Figure S3. SAPCD2 Regulates Cell Proliferation in Bladder Cancer. (A, B) EdU incorporation assays showing a significant decrease in the proportion of EdU-positive cells upon SAPCD2 silencing in T24 and UMUC3 cells. (C, D) CCK-8 assays confirming reduced proliferation in T24 and UMUC3 cells after SAPCD2 knockdown. (E, F) EdU incorporation

assays demonstrating increased proliferative capacity in J82 and 5637 cells with SAPCD2 overexpression (Original magnification, ×10; scale bar = 100 μm). (G, H) CCK-8 assays supporting enhanced cell proliferation in J82 and 5637 cells following SAPCD2 overexpression. (I) RT-qPCR confirming stable SAPCD2 knockdown in T24 cells using shSAPCD2.

4. Figure S4

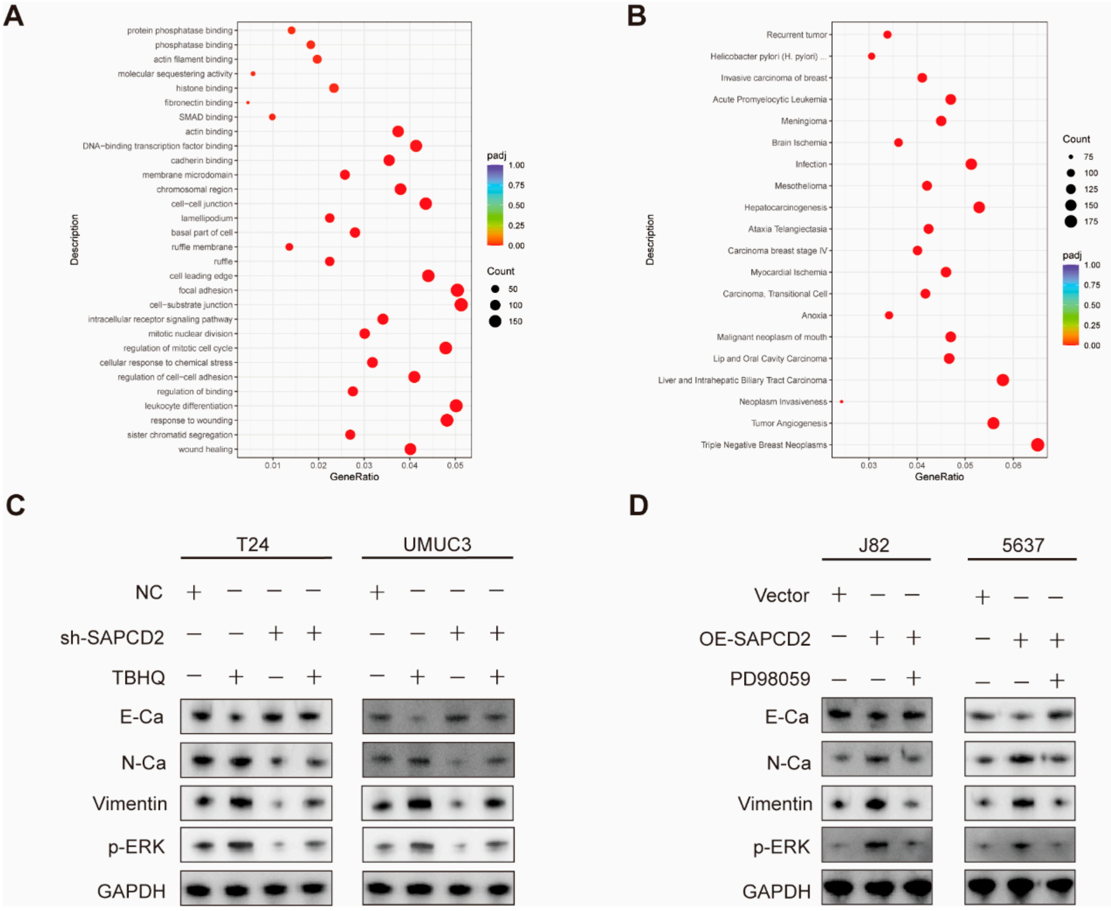

Figure S4. Gene Enrichment Analysis and EMT Regulation by SAPCD2 in Bladder Cancer Cells. (A) Gene Ontology (GO) analysis showing significant enrichment in biological processes. (B) DisGeNET enrichment analysis linking SAPCD2-associated gene signatures to tumor-related terms. (C) TBHQ treatment rescues the suppression of EMT induced by SAPCD2 depletion, as indicated by the re-expression of mesenchymal markers and reduced epithelial markers. (D) PD98059 reverses SAPCD2 overexpression induced EMT marker changes.

5. Figure S5

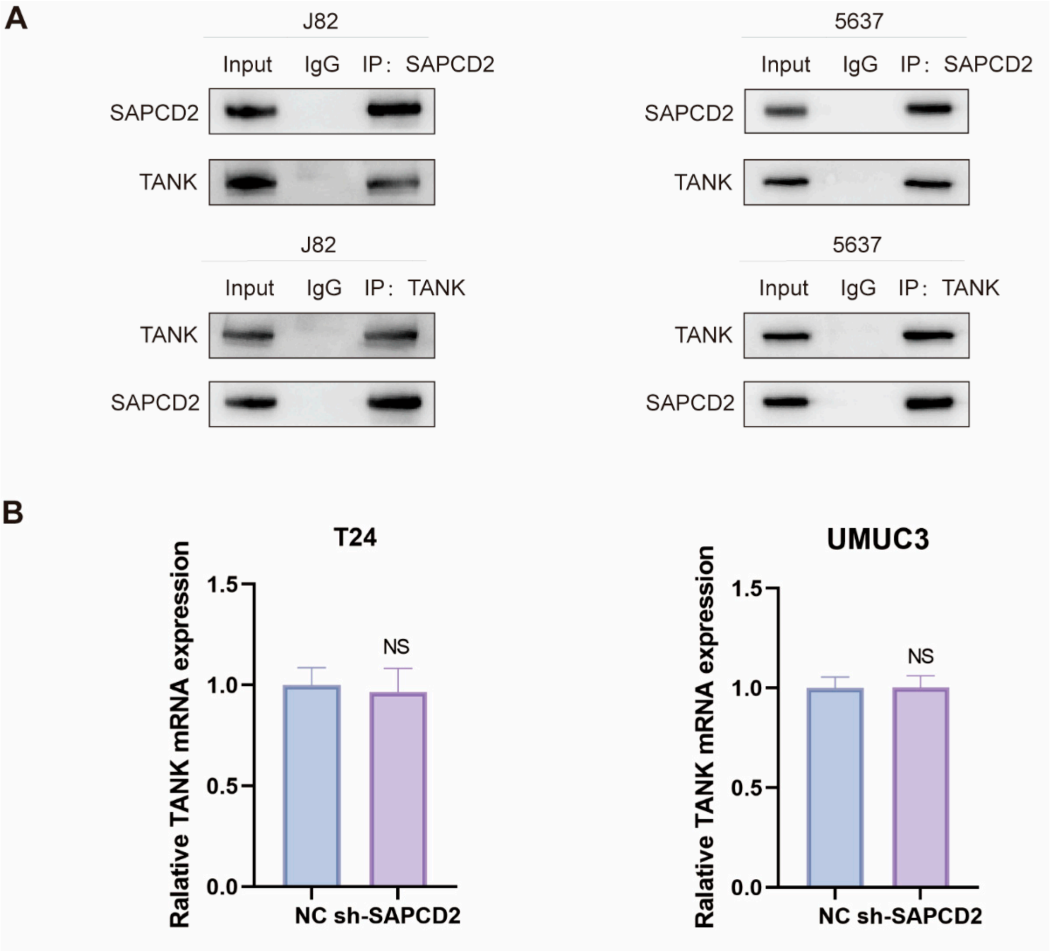

Figure S5. Interaction Between SAPCD2 and TANK, and Impact on TANK mRNA Expression. (A) Co-immunoprecipitation (CO-IP) assays confirming the interaction between SAPCD2 and TANK in both J82 and 5637 bladder cancer cells under endogenous conditions. (B) TANK mRNA expression in T24 and UMUC3 cells after SAPCD2 knockdown.

6. Figure S6

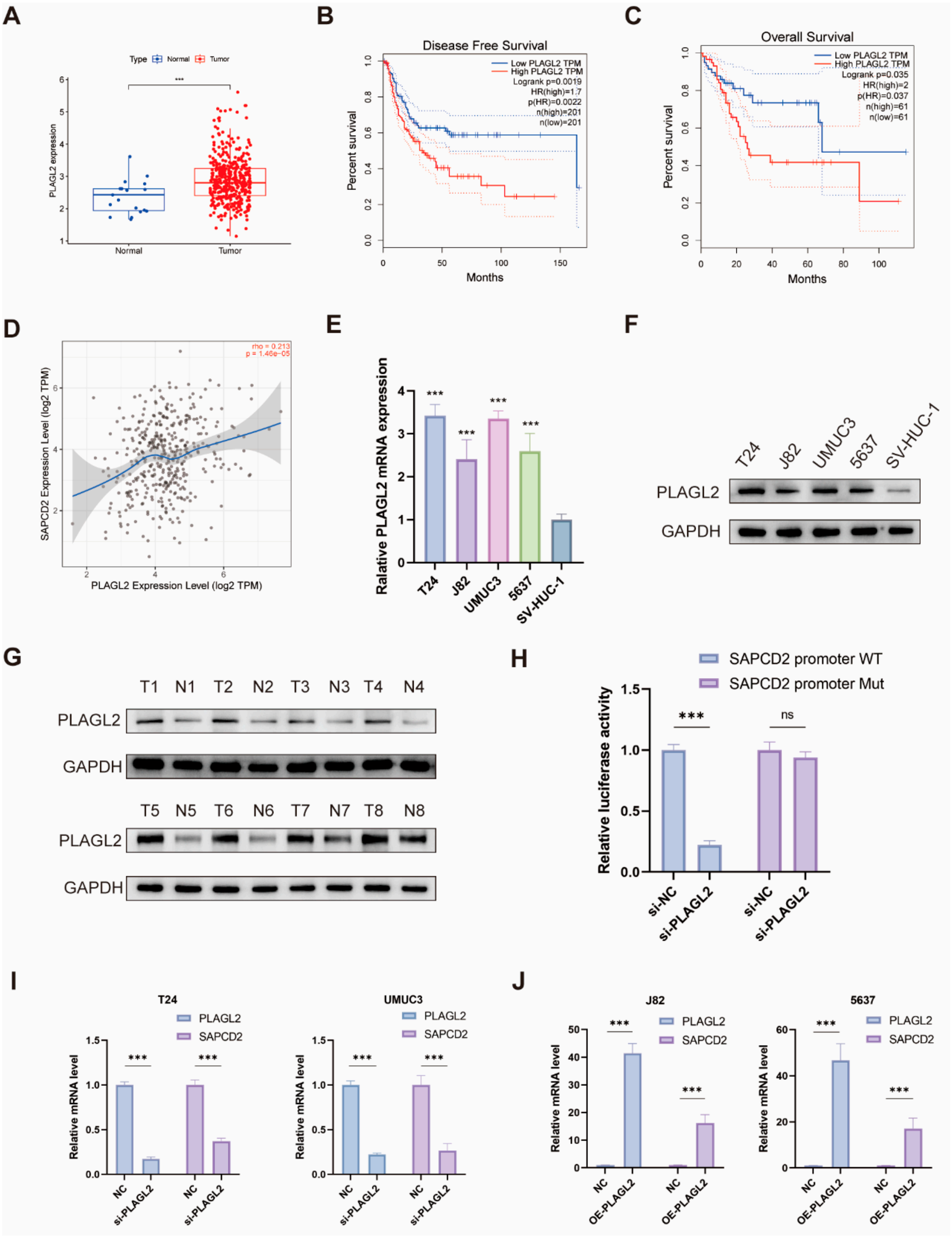

Figure S6. Upstream Regulation of SAPCD2 by PLAGL2 in Bladder Cancer. (A) PLAGL2 expression in bladder cancer tissues and normal bladder tissues, showing significant upregulation in tumors. (B, C) Kaplan-Meier survival analysis indicating that higher PLAGL2 expression correlates with poorer disease-free survival (DFS) and overall survival (OS) in bladder cancer patients. (D) Correlation between PLAGL2 and SAPCD2 expression in bladder cancer. (E) PLAGL2 mRNA expression is elevated in bladder cancer cell lines. (F) PLAGL2

protein levels are increased in bladder cancer cell lines. (G) PLAGL2 expression is elevated in bladder cancer tissues compared to normal bladder tissues. (H) Dual-luciferase reporter assays showing that PLAGL2 depletion decreases luciferase activity in cells with the wild-type SAPCD2 promoter, but has no effect on the mutant promoter. (I) PLAGL2 knockdown reduces mRNA levels of SAPCD2 in T24 and UMUC3 cells. (J) PLAGL2 overexpression increases mRNA levels of SAPCD2 in bladder cancer cells.

7. Figure S7

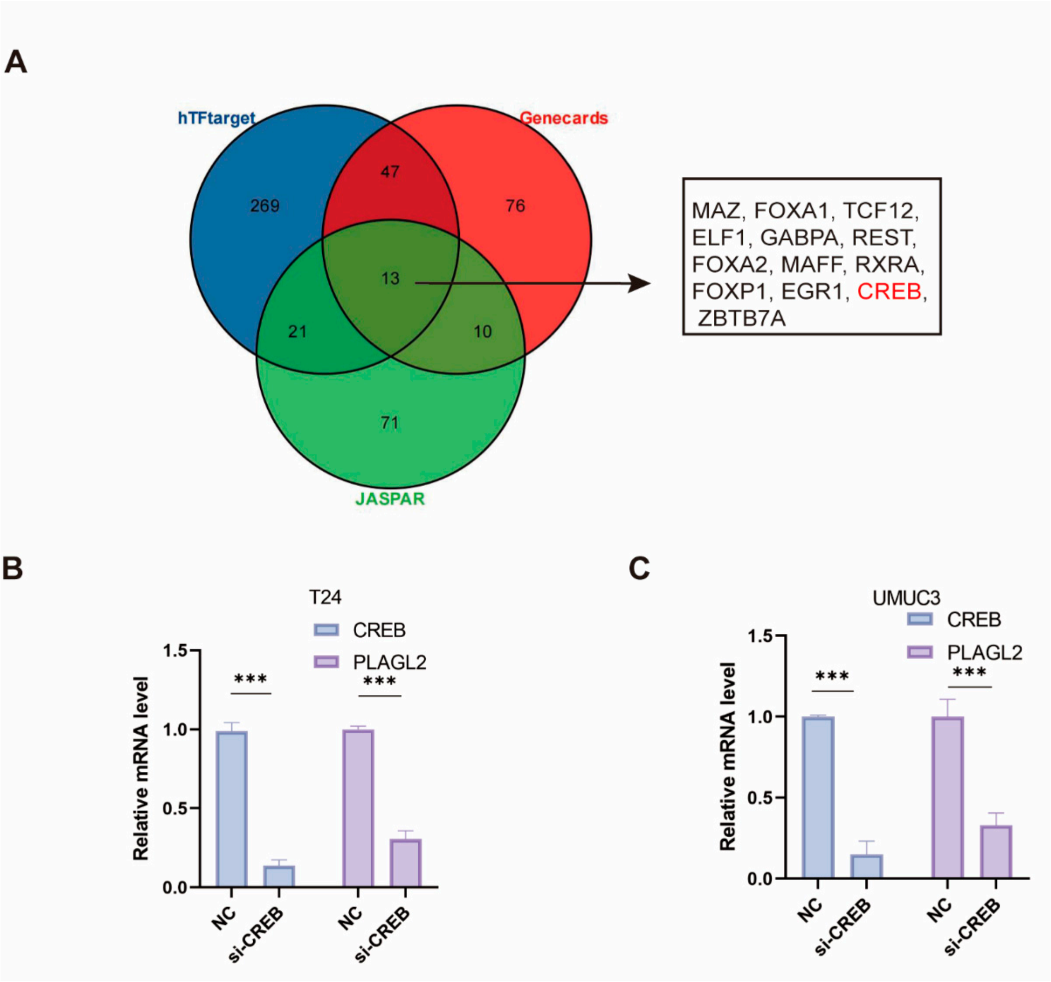

Figure S7. Bioinformatic Prediction and CREB Regulation of PLAGL2 Expression. (A) Venn diagram showing the overlap of predicted upstream transcription factors of PLAGL2 from the hTFtarget, GeneCards, and JASPAR databases, with 13 common factors identified, including CREB. (B, C) qRT-PCR analysis showing that silencing of CREB significantly reduces PLAGL2 mRNA expression in T24 (S7B) and UMUC3 (S7C) cells.

Supplementary tables

1. Table S1: PCR primers sequence

Primer name	Forward (5'-3')	Reverse (5'-3')
SAPCD2	GCAGACCATCCTCATGCTGAA	CGTGATGCGCTCACTCTTCT
TANK	CCACTTCTGGACCCATCTGATG	GCAGTTCTGAGTCTGTGCCACT
PLAGL2	GAGTCAAGTGAAGTGCCAATGT	TGAGGGCAGCTATATGGTCTC
CREB	GAGAAGCGGAGTGTTGGTGA	TCCGTCACTGCTTTCGTTCA
GAPDH	AGGTGAAGGTCGGAGTCAACG	AGGGGTCATTGATGGCAACA

2. Table S2: Antibody catalog numbers and dilutions for WB.

Antibody name	catalog numbers	Dilutions
SAPCD2	Bioss, BS-15314R	1:1000
GAPDH	Proteintech, 60004-1-Ig	1:80000
E-cadherin	Proteintech, 20874-1-AP	1:40000
N-cadherin	Proteintech, 22018-1-AP	1:8000
Vimentin	Proteintech, 10366-1-AP	1:40000
MEK	Proteintech, 11049-1-AP	1:8000
p-MEK	CST, 9154	1:1000
ERK	Proteintech, 11257-1-AP	1:4000
p-ERK	CST, 4370	1:2000
TANK	abcam, ab47632	1:500
His	Proteintech, 66005-1-Ig	1:10000
Flag	Proteintech, 66008-4-Ig	1:10000
Myc	Proteintech, 60003-2-Ig	1:10000
Ubiquitin	CST, 3936	1:1000
CREB	Proteintech, 67927-1-Ig	1:10000
p-CREB	Proteintech, 28792-1-AP	1:6000
HRP-conjugated Goat Anti-Rabbit IgG(H+L)	Proteintech, SA00001-2	1:10000
HRP-conjugated Goat Anti-Mouse IgG(H+L)	Proteintech, SA00001-1	1:10000

3. Table S3: The sequences of the siRNAs

	(5' - 3')
si-SAPCD2-1	GCAGCAGACCAUCCUCAUGTT
si-SAPCD2-2	CCAGCGGCUACCUGACCUUTT
si-PLAGL2	GCUCUGUUAUGGAGCCUUATT
si-CREB	AACCAAGTTGTTGTTCAAGCT

4. Table S4: The sequences of the shRNAs

	(5'-3')
sh-SAPCD2	GCGCTCATTAAGCAGCTGTTT
sh-TANK	GCAGACAACATAAACATCTTT
Negative control	TTCTCCGAACGTGTCACGT

5. Table S5: The clinical information of the patients.

Characteristics	Patients
Sex	
Male	6 (75%)
Female	2 (25%)
Age	
<=60	2 (25%)
>60	6 (75%)
T status	
T1-2	4 (50%)
T3-4	4 (50%)
N status	
N0	7 (77.5%)
N1	1 (12.5%)
Grade	
Low	7 (77.5%)
High	1 (12.5%)

6. Table S6. LC/MS results.

Accession	Gene Name	Description	Coverage [%]	# Peptides	# Unique Peptides	# AAs	MW [kDa]
Q86UD0	SAPCD2	Suppressor APC domain-containing protein 2 OS=Homo sapiens OX=9606 GN=SAPCD2 PE=1 SV=2	43	18	18	394	42.6
P29475	NOS1	Nitric oxide synthase 1 OS=Homo sapiens OX=9606 GN=NOS1 PE=1 SV=2	16	18	18	1434	161
O95153	TSPOAP1	Peripheral-type benzodiazepine receptor-associated protein 1 OS=Homo sapiens OX=9606 GN=TSPOAP1 PE=1 SV=2	3	17	17	1857	200.1
Q6IQ23	PLEKHA7	Pleckstrin homology domain-containing family A member 7 OS=Homo	10	14	14	1121	127.1

		sapiens OX=9606 GN=PLEKHA7 PE=1 SV=2					
Q92844	TANK	TRAF family member-associated NF-kappa-B activator OS=Homo sapiens OX=9606 GN=TANK PE=1 SV=2	30	14	14	425	47.8
Q69YH5	CDCA2	Cell division cycle-associated protein 2 OS=Homo sapiens OX=9606 GN=CDCA2 PE=1 SV=2	14	12	12	1023	112.7
P51797	CLCN6 KIAA0046	H(+)/Cl(-) exchange transporter 6 OS=Homo sapiens OX=9606 GN=CLCN6 PE=1 SV=2	16	14	12	869	92.3
O14544	SOCS6	Suppressor of cytokine signaling 6 OS=Homo sapiens OX=9606	21	11	11	535	59.5

		GN=SOCS 6 PE=1 SV=2					
P15428	HPGD	15-hydroxyprostaglandin dehydrogenase [NAD(+)] OS=Homo sapiens OX=9606 GN=HPGD PE=1 SV=1	32	10	10	266	29
Q6UW63	POGLUT2 EP58 KDELC1 UNQ1910/PRO4357	Protein O-glucosyltransferase 2 OS=Homo sapiens OX=9606 GN=POGLUT2 PE=1 SV=1	29	10	10	502	58
O75319	DUSP11	RNA/RNP complex-1-interacting phosphatase OS=Homo sapiens OX=9606 GN=DUSP11 PE=1 SV=3	25	10	10	330	38.9
Q6ZNC8	MBOAT1	Membrane-bound glycerophospholipid O-acyltransferase 1 OS=Homo sapiens	18	10	10	495	56.6

		OX=9606 GN=MBOAT1 PE=1 SV=1					
P22732	SLC2A5	Solute carrier family 2, facilitated glucose transporter member 5 OS=Homo sapiens OX=9606 GN=SLC2A5 PE=1 SV=1	24	9	9	501	55
O75175	CNOT3	CCR4-NOT transcription complex subunit 3 OS=Homo sapiens OX=9606 GN=CNOT3 PE=1 SV=1	13	9	9	753	81.9
P23921	RRM1	Ribonucleoside-diphosphate reductase large subunit OS=Homo sapiens OX=9606 GN=RRM1 PE=1 SV=1	11	9	9	792	90.1
P62750	RPL23A	60S ribosomal protein L23a	35	8	8	156	17.7

		OS=Homo sapiens OX=9606 GN=RPL23A PE=1 SV=1					
P02649	APOE	Apolipoprotein E OS=Homo sapiens OX=9606 GN=APOE PE=1 SV=1	33	8	8	317	36.2
P14868	DARS1	Aspartate--tRNA ligase, cytoplasmic OS=Homo sapiens OX=9606 GN=DARS1 PE=1 SV=2	18	8	8	501	57.1
Q155Q3	DIXDC1 CCD1 KIAA1735	Dixin OS=Homo sapiens OX=9606 GN=DIXDC1 PE=1 SV=2	12	8	8	683	77.5
P29536	LMOD1	Leiomodin-1 OS=Homo sapiens OX=9606 GN=LMOD1 PE=1 SV=3	9	8	8	600	67
Q14188	TFDP2 DP2	Transcription factor Dp-2 OS=Homo	23	8	7	446	49.2

		sapiens OX=9606 GN=TFDP2 PE=1 SV=2					
P21796	VDAC1	Voltage-dependent anion-selective channel protein 1 OS=Homo sapiens OX=9606 GN=VDAC1 PE=1 SV=2	28	7	7	283	30.8
P29508	SERPINB3 SCCA SCCA1	Serpin B3 OS=Homo sapiens OX=9606 GN=SERPINB3 PE=1 SV=2	17	7	7	390	44.6
A8MUP2	CSKMT	Citrate synthase-lysine N-methyltransferase CSKMT, mitochondrial OS=Homo sapiens OX=9606 GN=CSKMT PE=1 SV=1	25	6	6	240	25.9
P25445	FAS	Tumor necrosis factor receptor superfamily member 6	23	6	6	335	37.7

		OS=Homo sapiens OX=9606 GN=FAS PE=1 SV=1					
P51946	CCNH	Cyclin-H OS=Homo sapiens OX=9606 GN=CCNH PE=1 SV=1	17	6	6	323	37.6
P47900	P2RY1	P2Y purinocepto r 1 OS=Homo sapiens OX=9606 GN=P2RY 1 PE=1 SV=1	14	6	6	373	42.1
Q8N344	MIER2	Mesoderm induction early response protein 2 OS=Homo sapiens OX=9606 GN=MIER 2 PE=1 SV=2	12	6	6	545	59.9
Q5UE93	PIK3R6 C17orf38	Phosphoino sitide 3-kinase regulatory subunit 6 OS=Homo sapiens OX=9606 GN=PIK3R 6 PE=1 SV=1	7	6	6	754	84.3

Q8TB40	ABHD4	(Lyso)-N-acylphosphatidylethanolamine lipase OS=Homo sapiens OX=9606 GN=ABHD4 PE=1 SV=1	3	6	6	342	38.8
Q8NF50	DOCK8	Dedicator of cytokinesis protein 8 OS=Homo sapiens OX=9606 GN=DOCK8 PE=1 SV=3	2	6	6	2099	238.5
P19367	HK1	Hexokinase-1 OS=Homo sapiens OX=9606 GN=HK1 PE=1 SV=3	6	5	5	917	102.5
P24534	EEF1B2	Elongation factor 1-beta OS=Homo sapiens OX=9606 GN=EEF1B2 PE=1 SV=3	38	8	5	225	24.7
O43155	FLRT2	Leucine-rich repeat transmembrane protein FLRT2 OS=Homo	9	7	5	660	74

		sapiens OX=9606 GN=FLRT2 PE=1 SV=1					
P11055	MYH3	Myosin-3 OS=Homo sapiens OX=9606 GN=MYH3 PE=1 SV=3	3	6	5	1940	223.9
P68543	UBXN2A UBXD4	UBX domain-containing protein 2A OS=Homo sapiens OX=9606 GN=UBXN2A PE=1 SV=1	19	5	5	259	29.3
A6NDG6	PGP	Glycerol-3-phosphate phosphatase OS=Homo sapiens OX=9606 GN=PGP PE=1 SV=1	19	5	5	321	34
P09467	FBP1	Fructose-1,6-bisphosphatase 1 OS=Homo sapiens OX=9606 GN=FBP1 PE=1 SV=5	16	5	5	338	36.8
P48735	IDH2	Isocitrate dehydrogen	13	5	5	452	50.9

		ase [NADP], mitochondrial OS=Homo sapiens OX=9606 GN=IDH2 PE=1 SV=2					
P36897	TGFBR1 ALK5 SKR4	TGF-beta receptor type-1 OS=Homo sapiens OX=9606 GN=TGFBR1 PE=1 SV=1	12	5	5	503	56
Q96JF0	ST6GAL2	Beta-galactoside alpha-2,6-sialyltransferase 2 OS=Homo sapiens OX=9606 GN=ST6GAL2 PE=1 SV=2	12	5	5	529	60.2
P09086	POU2F2	POU domain, class 2, transcription factor 2 OS=Homo sapiens OX=9606 GN=POU2F2 PE=1 SV=3	10	5	5	479	51.2
Q8TCC7	SLC22A8 OAT3	Organic anion transporter	10	5	5	542	59.9

		3 OS=Homo sapiens OX=9606 GN=SLC22A8 PE=1 SV=1					
Q10570	CPSF1	Cleavage and polyadenylation specificity factor subunit 1 OS=Homo sapiens OX=9606 GN=CPSF1 PE=1 SV=2	5	5	5	1443	160.8
Q5T3U5	ABCC10 MRP7 SIMRP7	ATP-binding cassette sub-family C member 10 OS=Homo sapiens OX=9606 GN=ABCC10 PE=1 SV=1	3	5	5	1492	161.6
P25929	NPY1R	Neuropeptide Y receptor type 1 OS=Homo sapiens OX=9606 GN=NPY1R PE=1 SV=1	23	10	4	384	44.4
Q9Y265	RUVBL1	RuvB-like 1	23	9	4	456	50.2

		OS=Homo sapiens OX=9606 GN=RUVBL1 PE=1 SV=1					
P46695	IER3 DIF2 IEX1 PRG1	Radiation-inducible immediate-early gene IEX-1 OS=Homo sapiens OX=9606 GN=IER3 PE=1 SV=4	21	4	4	156	16.9
P18510	IL1RN	Interleukin-1 receptor antagonist protein OS=Homo sapiens OX=9606 GN=IL1RN PE=1 SV=1	20	4	4	177	20.1
Q496M5	PLK5 PLK5P FG060302	Inactive serine/threonine-protein kinase PLK5 OS=Homo sapiens OX=9606 GN=PLK5 PE=1 SV=4	17	4	4	336	36.3
Q02548	PAX5	Paired box protein Pax-5 OS=Homo sapiens	17	4	4	391	42.1

		OX=9606 GN=PAX5 PE=1 SV=1					
Q9HBY0	NOX3	NADPH oxidase 3 OS=Homo sapiens OX=9606 GN=NOX3 PE=1 SV=1	17	4	4	568	64.9
Q9C030	TRIM6	Tripartite motif-containing protein 6 OS=Homo sapiens OX=9606 GN=TRIM 6 PE=1 SV=1	15	4	4	488	56.4
Q3ZCM7	TUBB8	Tubulin beta-8 chain OS=Homo sapiens OX=9606 GN=TUBB 8 PE=1 SV=2	11	4	4	444	49.8
P04040	CAT	Catalase OS=Homo sapiens OX=9606 GN=CAT PE=1 SV=3	10	4	4	527	59.7
P21728	DRD1	D(1A) dopamine receptor OS=Homo sapiens OX=9606	9	4	4	446	49.3

		GN=DRD1 PE=1 SV=1					
P42331	ARHGAP25 KIAA0053	Rho GTPase-activating protein 25 OS=Homo sapiens OX=9606 GN=ARHGAP25 PE=1 SV=2	5	4	4	645	73.4
Q2TAC2	CCDC57	Coiled-coil domain-containing protein 57 OS=Homo sapiens OX=9606 GN=CCDC57 PE=1 SV=3	3	4	4	915	103
O95622	ADCY5	Adenylate cyclase type 5 OS=Homo sapiens OX=9606 GN=ADCY5 PE=1 SV=3	2	4	4	1261	138.9
Q8WVM7	STAG1 SA1 SCC3	Cohesin subunit SA-1 OS=Homo sapiens OX=9606 GN=STAG1 PE=1 SV=3	2	4	4	1258	144.4
Q8TBX8	PIP4K2C PIP5K2C	Phosphatidylinositol	12	4	3	421	47.3

		5-phosphate 4-kinase type-2 gamma OS=Homo sapiens OX=9606 GN=PIP4K2C PE=1 SV=3					
Q02083	NAAA ASAHL PLT	N-acylethanolamine-hydrolyzing acid amidase OS=Homo sapiens OX=9606 GN=NAAA PE=1 SV=3	10	4	3	359	40.1
Q53S33	BOLA3	BolA-like protein 3 OS=Homo sapiens OX=9606 GN=BOLA3 PE=1 SV=1	34	3	3	107	12.1
P17096	HMGA1	High mobility group protein HMG-I/HMG-Y OS=Homo sapiens OX=9606 GN=HMGA1 PE=1 SV=3	33	3	3	107	11.7
P55854	SUMO3	Small	30	3	3	103	11.6

	SMT3A SMT3H1	ubiquitin-related modifier 3 OS=Homo sapiens OX=9606 GN=SUMO3 PE=1 SV=2					
Q9Y5I7	CLDN16	Claudin-16 OS=Homo sapiens OX=9606 GN=CLDN16 PE=1 SV=2	25	3	3	235	26.1
P46783	RPS10	Small ribosomal subunit protein eS10 OS=Homo sapiens OX=9606 GN=RPS10 PE=1 SV=1	24	3	3	165	18.9
O00585	CCL21	C-C motif chemokine 21 OS=Homo sapiens OX=9606 GN=CCL21 PE=1 SV=1	23	3	3	134	14.6
P0DI82	TRAPPC2B	Trafficking protein particle complex subunit 2B OS=Homo sapiens OX=9606	19	3	3	140	16.4

		GN=TRAP PC2B PE=1 SV=1					
P10916	MYL2	Myosin regulatory light chain 2, ventricular/ cardiac muscle isoform OS=Homo sapiens OX=9606 GN=MYL2 PE=1 SV=3	15	3	3	166	18.8
Q969U7	PSMG2	Proteasome assembly chaperone 2 OS=Homo sapiens OX=9606 GN=PSMG 2 PE=1 SV=1	14	3	3	264	29.4
P09466	PAEP	Glycodelin OS=Homo sapiens OX=9606 GN=PAEP PE=1 SV=2	10	3	3	180	20.6
A1A519	FAM170 A	Protein FAM170A OS=Homo sapiens OX=9606 GN=FAM1 70A PE=1 SV=1	9	3	3	330	37.2
Q12836	ZP4 ZPB	Zona	8	3	3	540	59.4

		pellucida sperm-binding protein 4 OS=Homo sapiens OX=9606 GN=ZP4 PE=1 SV=1					
P02679	FGG	Fibrinogen gamma chain OS=Homo sapiens OX=9606 GN=FGG PE=1 SV=3	5	3	3	453	51.5
P59827	BPIFB4 C20orf186 LPLUNC4	BPI fold-containing family B member 4 OS=Homo sapiens OX=9606 GN=BPIFB4 PE=1 SV=2	5	3	3	614	65.1
O96005	CLPTM1	Putative lipid scramblase CLPTM1 OS=Homo sapiens OX=9606 GN=CLPTM1 PE=1 SV=1	4	3	3	669	76.1
P42224	STAT1	Signal transducer and activator of transcriptio	4	3	3	750	87.3

		n 1-alpha/beta OS=Homo sapiens OX=9606 GN=STAT1 PE=1 SV=2					
Q14563	SEMA3A SEMAD	Semaphorin-3A OS=Homo sapiens OX=9606 GN=SEMA3A PE=1 SV=1	4	3	3	771	88.9
Q5W041	ARMC3	Armadillo repeat-containing protein 3 OS=Homo sapiens OX=9606 GN=ARMC3 PE=1 SV=2	4	3	3	872	96.4
Q5HYW2	NHSL2	NHSL2_HUMAN NHS-like protein 2 OS=Homo sapiens OX=9606 GN=NHSL2 PE=1 SV=2	3	3	3	1225	133.3
Q86VX9	MON1A	Vacuolar fusion protein MON1 homolog A OS=Homo sapiens OX=9606	3	3	3	652	72.9

		GN=MON 1A PE=1 SV=3					
Q13976	PRKG1 PRKG1B PRKGR1 A PRKGR1 B	cGMP-dependent protein kinase 1 OS=Homo sapiens OX=9606 GN=PRKG 1 PE=1 SV=3	2	3	3	671	76.4
Q9H4Z2	ZNF335	Zinc finger protein 335 OS=Homo sapiens OX=9606 GN=ZNF3 35 PE=1 SV=1	2	3	3	1342	144.9
P04275	VWF	von Willebrand factor OS=Homo sapiens OX=9606 GN=VWF PE=1 SV=4	1	3	3	2813	309.3
O95758	PTBP3	Polypyrimi dine tract-binding protein 3 OS=Homo sapiens OX=9606 GN=PTBP 3 PE=1 SV=2	7	4	2	552	59.7
P11362	FGFR1	Fibroblast growth factor receptor	7	4	2	822	91.9

		1OS=Homo sapiens OX=9606 GN=FGFR1 PE=1 SV=2					
O14625	CXCL11	C-X-C motif chemokine 11 OS=Homo sapiens OX=9606 GN=CXCL11 PE=1 SV=1	26	2	2	94	10.3
Q16718	NDUFA5	NADH dehydrogenase [ubiquinone] 1 alpha subcomplex subunit 5 OS=Homo sapiens OX=9606 GN=NDUFA5 PE=1 SV=3	19	2	2	116	13.5
O96004	HAND1	Heart- and neural crest derivatives-expressed protein 1 OS=Homo sapiens OX=9606 GN=HAND1 PE=1 SV=1	16	2	2	215	23.6
P28360	MSX1 HOX7	Homeobox protein MSX-1 OS=Homo	13	2	2	303	31.5

		sapiens OX=9606 GN=MSX1 PE=1 SV=3					
A0A0K2S4Q6	CD300H	Protein CD300H OS=Homo sapiens OX=9606 GN=CD300H PE=1 SV=1	12	2	2	201	21.8
P0DI83	RAB34	Ras-related protein Rab-34, isoform NARR OS=Homo sapiens OX=9606 GN=RAB34 PE=1 SV=1	11	2	2	198	21.1
Q9H320	VCX	Variable charge X-linked protein 1 OS=Homo sapiens OX=9606 GN=VCX PE=2 SV=1	10	2	2	206	22.3
P62745	RHOB ARH6 ARHB	Rho-related GTP-binding protein RhoB OS=Homo sapiens OX=9606 GN=RHOB PE=1	9	2	2	196	22.1

		SV=1					
P11686	SFTPC	Surfactant protein C OS=Homo sapiens OX=9606 GN=SFTPC PE=1 SV=3	8	2	2	197	21
Q6IN84	MRM1	rRNA methyltransferase 1, mitochondrial OS=Homo sapiens OX=9606 GN=MRM1 PE=1 SV=1	8	2	2	353	38.6
Q9P2A4	ABI3	ABI gene family member 3 OS=Homo sapiens OX=9606 GN=ABI3 PE=1 SV=2	8	2	2	366	39
O15400	STX7	Syntaxin-7 OS=Homo sapiens OX=9606 GN=STX7 PE=1 SV=4	6	2	2	261	29.8
P04201	MAS1	Proto-oncogene Mas OS=Homo sapiens OX=9606 GN=MAS1 PE=1	6	2	2	325	37.5

		SV=1					
Q9NUV9	GIMAP4	GTPase IMAP family member 4 OS=Homo sapiens OX=9606 GN=GIMAP4 PE=1 SV=1	6	2	2	329	37.5
O43813	LANCL1	Glutathione S-transferase LANCL1 OS=Homo sapiens OX=9606 GN=LANCL1 PE=1 SV=1	5	2	2	399	45.3
P27352	CBLIF	Cobalamin binding intrinsic factor OS=Homo sapiens OX=9606 GN=CBLIF PE=1 SV=2	5	2	2	417	45.4
Q8N6Y0	USHBP1 AIEBP MCC2	Harmonin-binding protein USHBP1 OS=Homo sapiens OX=9606 GN=USHBP1 PE=1 SV=1	5	2	2	703	76.1
P14136	GFAP	Glial fibrillary acidic	5	2	2	432	49.9

		protein OS=Homo sapiens OX=9606 GN=GFAP PE=1 SV=1					
Q8WWH4	ASZ1	Ankyrin repeat, SAM and basic leucine zipper domain-containing protein 1 OS=Homo sapiens OX=9606 GN=ASZ1 PE=1 SV=1	5	2	2	475	53.5
P05177	CYP1A2	Cytochrome P450 1A2 OS=Homo sapiens OX=9606 GN=CYP1A2 PE=1 SV=4	4	2	2	516	58.4
O75564	JRK	Jerky protein homolog OS=Homo sapiens OX=9606 GN=JRK PE=1 SV=3	3	2	2	556	61.8
P15391	CD19	B-lymphocyte antigen CD19 OS=Homo	3	2	2	556	61.1

		sapiens OX=9606 GN=CD19 PE=1 SV=6					
Q13275	SEMA3F	Semaphorin-3F OS=Homo sapiens OX=9606 GN=SEMA3F PE=1 SV=2	3	2	2	785	88.4
Q14995	NR1D2	Nuclear receptor subfamily 1 group D member 2 OS=Homo sapiens OX=9606 GN=NR1D2 PE=1 SV=4	3	2	2	579	64.6
Q16584	MAP3K11 MLK3 PTK1 SPRK	Mitogen-activated protein kinase kinase kinase 11 OS=Homo sapiens OX=9606 GN=MAP3K11 PE=1 SV=1	3	2	2	847	92.7
O43548	TGM5	Protein-glutamine gamma-glutamyltransferase 5 OS=Homo sapiens OX=9606	2	2	2	720	80.8

		GN=TGM5 PE=1 SV=4					
P12830	CDH1	Cadherin-1 OS=Homo sapiens OX=9606 GN=CDH1 PE=1 SV=3	2	2	2	882	97.5
P98155	VLDLR	Very low-density lipoprotein receptor OS=Homo sapiens OX=9606 GN=VLDLR PE=1 SV=1	2	2	2	873	96.1
Q7Z4V0	ZNF438	Zinc finger protein 438 OS=Homo sapiens OX=9606 GN=ZNF438 PE=1 SV=1	2	2	2	828	91.8
P08922	ROS1	Proto-oncogene tyrosine-protein kinase ROS OS=Homo sapiens OX=9606 GN=ROS1 PE=1 SV=3	1	2	2	2347	263.9
P05106	ITGB3	Integrin beta-3 OS=Homo sapiens OX=9606	12	8	1	788	87.1

		GN=ITGB 3 PE=1 SV=2					
Q9BQS2	SYT15	Synaptotagmin-15 OS=Homo sapiens OX=9606 GN=SYT15 PE=1 SV=3	9	4	1	421	47.4
Q5T4F7	SFRP5 FRP1B SARP3	Secreted frizzled-related protein 5 OS=Homo sapiens OX=9606 GN=SFRP5 PE=1 SV=3	16	3	1	317	35.6
P55809	OXCT1 OXCT SCOT	Succinyl-CoA:3-ketoacid coenzyme A transferase 1, mitochondrial OS=Homo sapiens OX=9606 GN=OXCT1 PE=1 SV=1	4	2	1	520	56.2
O75928	PIAS2	E3 SUMO-protein ligase PIAS2 OS=Homo sapiens OX=9606 GN=PIAS2	2	2	1	621	68.2

		PE=1 SV=3					
P59666	DEFA3 DEF3	Neutrophil defensin 3 OS=Homo sapiens OX=9606 GN=DEFA3 PE=1 SV=1	15	1	1	94	10.2
Q15329	E2F5	Transcription factor E2F5 OS=Homo sapiens OX=9606 GN=E2F5 PE=1 SV=1	15	1	1	346	37.6
P61088	UBE2N	Ubiquitin-conjugating enzyme E2 N OS=Homo sapiens OX=9606 GN=UBE2N PE=1 SV=1	15	1	1	152	17.1
P61927	RPL37	Large ribosomal subunit protein eL37 OS=Homo sapiens OX=9606 GN=RPL37 PE=1 SV=2	12	1	1	97	11.1
Q9Y6H6	KCNE3	Potassium voltage-gated channel	12	1	1	103	11.7

		subfamily E member 3 OS=Homo sapiens OX=9606 GN=KCNE3 PE=1 SV=1					
P27449	ATP6V0C ATP6C ATP6L ATPL	V-type proton ATPase 16 kDa proteolipid subunit c OS=Homo sapiens OX=9606 GN=ATP6V0C PE=1 SV=1	10	1	1	155	15.7
Q9UKK6	NXT1	NTF2-related export protein 1 OS=Homo sapiens OX=9606 GN=NXT1 PE=1 SV=1	10	1	1	140	15.8
Q9Y3D3	MRPS16	Small ribosomal subunit protein bS16m OS=Homo sapiens OX=9606 GN=MRPS16 PE=1 SV=1	10	1	1	137	15.3
O75443	TECTA	Alpha-tectorin	9	1	1	2155	24

		OS=Homo sapiens OX=9606 GN=TECTA PE=1 SV=3					
Q14686	NCOA6 AIB3 KIAA0181 RAP250 TRBP	Nuclear receptor coactivator 6 OS=Homo sapiens OX=9606 GN=NCOA6 PE=1 SV=3	8	1	1	2063	21.9
O96000	NDUFB10	NADH dehydrogenase [ubiquinone] 1 beta subcomplex subunit 10 OS=Homo sapiens OX=9606 GN=NDUFB10 PE=1 SV=3	6	1	1	172	20.8
P49771	FLT3LG	Fms-related tyrosine kinase 3 ligand OS=Homo sapiens OX=9606 GN=FLT3LG PE=1 SV=1	6	1	1	235	26.4
Q00688	FKBP3	Peptidyl-prolyl cis-trans isomerase	5	1	1	224	25.2

		FKBP3 OS=Homo sapiens OX=9606 GN=FKBP3 PE=1 SV=1					
P82979	SARNP	SAP domain-containing ribonucleoprotein OS=Homo sapiens OX=9606 GN=SARNP PE=1 SV=3	5	1	1	210	23.7
P83916	CBX1 CBX	Chromobox protein homolog 1 OS=Homo sapiens OX=9606 GN=CBX1 PE=1 SV=1	5	1	1	185	21.4
O95881	TXNDC12	Thioredoxin domain-containing protein 12 OS=Homo sapiens OX=9606 GN=TXNDC12 PE=1 SV=1	5	1	1	172	19.2
P11766	ADH5	Alcohol dehydrogenase class-3 OS=Homo sapiens OX=9606 GN=ADH5	5	1	1	374	39.7

		PE=1 SV=4					
Q9HD42	CHMP1 A	Charged multivesicular body protein 1a OS=Homo sapiens OX=9606 GN=CHMP1A PE=1 SV=1	4	1	1	196	21.7
P04062	GBA1	Lysosomal acid glucosylceramidase OS=Homo sapiens OX=9606 GN=GBA1 PE=1 SV=3	4	1	1	536	59.7
P67870	CSNK2B CK2N G5A	Casein kinase II subunit beta OS=Homo sapiens OX=9606 GN=CSNK2B PE=1 SV=1	4	1	1	215	24.9
O14788	TNFSF11	Tumor necrosis factor ligand superfamily member 11 OS=Homo sapiens OX=9606 GN=TNFSF11 PE=1 SV=1	4	1	1	317	35.5

Q9HC36	MRM3	rRNA methyltransferase 3, mitochondrial OS=Homo sapiens OX=9606 GN=MRM3 PE=1 SV=2	3	1	1	420	47
O43521	BCL2L11	Bcl-2-like protein 11 OS=Homo sapiens OX=9606 GN=BCL2L11 PE=1 SV=1	3	1	1	198	22.2
Q8IXA5	SPACA3	Sperm acrosome membrane-associated protein 3 OS=Homo sapiens OX=9606 GN=SPACA3 PE=1 SV=1	3	1	1	215	23.4
Q9HC52	CBX8	Chromobox protein homolog 8 OS=Homo sapiens OX=9606 GN=CBX8 PE=1 SV=3	2	1	1	389	43.4
O15232	MATN3	Matrilin-3 OS=Homo sapiens OX=9606 GN=MAT	2	1	1	486	52.8

		N3 PE=1 SV=2					
P19438	TNFRSF 1A	Tumor necrosis factor receptor superfamily member 1A OS=Homo sapiens OX=9606 GN=TNFR SF1A PE=1 SV=1	2	1	1	455	50.5
P78508	KCNJ10	ATP-sensitive inward rectifier potassium channel 10 OS=Homo sapiens OX=9606 GN=KCNJ 10 PE=1 SV=1	2	1	1	379	42.5
Q00537	CDK17 PCTAIR E2 PCTK2	Cyclin-dependent kinase 17 OS=Homo sapiens OX=9606 GN=CDK1 7 PE=1 SV=2	2	1	1	523	59.6
Q13630	GFUS SDR4E1 TSTA3	GDP-L-fucose synthase OS=Homo sapiens OX=9606 GN=GFUS PE=1 SV=1	2	1	1	321	35.9

Q5VTY9	HHAT MART2 SKI1	Protein-cysteine N-palmitoyltransferase HHAT OS=Homo sapiens OX=9606 GN=HHAT PE=1 SV=1	2	1	1	493	57.3
O15269	SPTLC1	Serine palmitoyltransferase 1 OS=Homo sapiens OX=9606 GN=SPTLC1 PE=1 SV=1	1	1	1	473	52.7
Q14416	GRM2 GPRC1B MGLUR2	Metabotropic glutamate receptor 2 OS=Homo sapiens OX=9606 GN=GRM2 PE=1 SV=2	1	1	1	872	95.6
A8K8P3	SFI1	Protein SFI1 homolog OS=Homo sapiens OX=9606 GN=SFI1 PE=1 SV=2	1	1	1	1242	147.7
P28340	POLD1	DNA polymerase delta catalytic subunit	1	1	1	1107	123.6

		OS=Homo sapiens OX=9606 GN=POLD1 PE=1 SV=2					
P29400	COL4A5	Collagen alpha-5(IV) chain OS=Homo sapiens OX=9606 GN=COL4A5 PE=1 SV=2	1	1	1	1685	161
P40145	ADCY8	Adenylate cyclase type 8 OS=Homo sapiens OX=9606 GN=ADCY8 PE=1 SV=1	1	1	1	1251	140.1
